# From tryptamine to the discovery of efficient multi-target directed ligands against cholinesterase-associated neurodegenerative disorders

**DOI:** 10.3389/fphar.2022.1036030

**Published:** 2022-11-28

**Authors:** Junbo Wu, Honghua Zhang, Yuying Wang, Gaofeng Yin, Qien Li, Linsheng Zhuo, Hongjin Chen, Zhen Wang

**Affiliations:** ^1^ Department of Colorectal Surgery, The Affiliated Hospital, Nanjing University of Chinese Medicine, Nanjing, Jiangsu, China; ^2^ Department of Colorectal Surgery, Hengyang Central Hospital, Hengyang, Hunan, China; ^3^ School of Pharmaceutical Science, Hengyang Medical School, University of South China, Hengyang, Hunan, China; ^4^ State Key Laboratory of Applied Organic Chemistry, College of Chemistry and Chemical Engineering, Lanzhou University, Lanzhou, China; ^5^ School of Pharmacy, Lanzhou University, Lanzhou, China; ^6^ Tibetan Medical College, Qinghai University, Xining, Qinghai, China; ^7^ The First Affiliated Hospital, Hengyang Medical School, University of South China, Hengyang, Hunan, China

**Keywords:** carbamylated tryptamine derivatives, benzylation, promising building blocks, MTDLs, cholinesterase inhibitors, neuroprotection, anti-neuroinflammation, neurodegenerative disorders

## Abstract

A novel class of benzyl-free and benzyl-substituted carbamylated tryptamine derivatives (CDTs) was designed and synthesized to serve as effective building blocks for the development of novel multi-target directed ligands (MTDLs) for the treatment of neurological disorders linked to cholinesterase (ChE) activity. The majority of them endowed butyrylcholinesterase (BuChE) with more substantial inhibition potency than acetylcholinesterase (AChE), according to the full study of ChE inhibition. Particularly, hybrids with dibenzyl groups (**2b-2f**, **2j**, **2o**, and **2q**) showed weak or no neuronal toxicity and hepatotoxicity and single-digit nanomolar inhibitory effects against BuChE. Through molecular docking and kinetic analyses, the potential mechanism of action on BuChE was first investigated. *In vitro* H_2_O_2_-induced HT-22 cells assay demonstrated the favorable neuroprotective potency of **2g**, **2h**, **2j**, **2m**, **2o**, and **2p**. Besides, **2g**, **2h**, **2j**, **2m**, **2o**, and **2p** endowed good antioxidant activities and COX-2 inhibitory effects. This study suggested that this series of hybrids can be applied to treat various ChE-associated neurodegenerative disorders such as Alzheimer’s disease (AD) and Parkinson’s disease (PD), as well as promising building blocks for further structure modification to develop efficient MTDLs.

## Introduction

**SCHEME 1:**
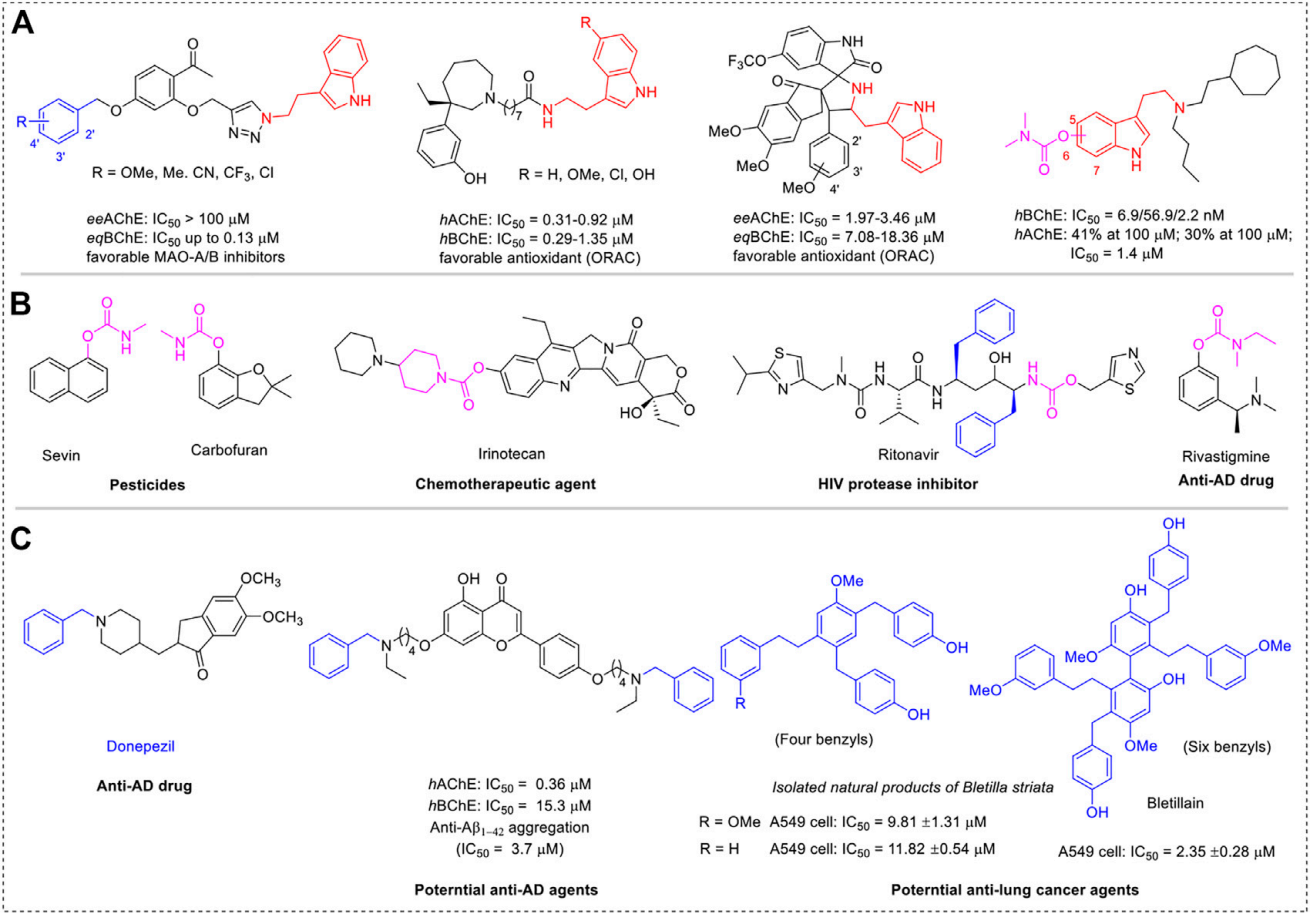
**(A)** Structures of several represented tryptamine derivatives. **(B)** Structures of several represented carbamate-based drugs. **(C)** Structures of several represented benzyl-containing agents with pharmaceutical activities.

Because of the complex pathogenesis, neurodegenerative diseases (NDs), including Alzheimer’s disease (AD), Parkinson’s disease (PD), Huntington’s disease (HD), and amyotrophic lateral sclerosis (ALS), remain unclear and incurable, have seriously endangered human health, and brought huge economic burden to society (Armstrong, 2020). Numerous recent studies have shown that multi-target directed ligands (MTDLs) are the preferred method for developing new drugs for NDs (Cavalli et al., 2008; Geldenhuys and Van der Schyf, 2013; Wichur and Malawska, 2015). Thereinto, molecular hybridization is the main approach to achieving MTDLs. Tryptamine, a monoamine alkaloid that contains indole, can function *in vivo* as a neuromodulator or neurotransmitter (Mousseau, 1993; Wu et al., 2018). Due to their wide range of biological activities and widespread use in medicinal chemistry (Scheme 1A), tryptamine and its analogs have attracted intense interest in industry and academia. Evidence has shown that tryptamine derivatives obtained using tryptamine as a key synthetic block endow significant pharmaceutical activities (Cheng et al., 2015; Kumar et al., 2021; Liu et al., 2022; Meden et al., 2022; Oh et al., 2022). Serotonin, also known as 5-hydroxytryptamine, plays an important biological role as a neurotransmitter in the cerebral cortex and synapses, making it a prominent target for drug development in immunological and neurological illnesses (Zhang et al., 2022a; Fang et al., 2022; Kim et al., 2022; Meden et al., 2022; Shevchenko et al., 2022; Wang et al., 2022). The most common optimization strategies for serotonin alteration are focused on hydroxyl and amino groups because of their intrinsic structural features.

Many neurodegenerative illnesses, including AD, PD, and HD, have been linked to cholinergic system alterations, and the loss of cholinergic transmission, including acetylcholine (ACh), is crucial for cognitive function (Hampel et al., 2018; Petrova et al., 2020; van der Zee et al., 2021; Bohnen et al., 2022). As a preferred pharmacophore of cholinesterase (ChE) inhibitors, carbamate fragments have been extensively exploited to produce extremely potent ChE inhibitors for the treatment of a wide range of ailments, including AD, HIV inhibitors, pesticides, and chemotherapeutic agents against various cancers (Scheme 1B) (Matosevic and Bosak, 2020; Martins et al., 2021; Mishra et al., 2021; Zhang et al., 2022b; Mdeni et al., 2022). This is mostly because of the structural properties of carbamate fragments, which can form bidentate H-bonds to anchor more firmly in the active pocket and act as an H-bond acceptor and donor. Additionally, adding a carbamate fragment can make molecules more drug-like and improve their metabolic stability. Therefore, a promising method to produce multifunctional ChE inhibitors is a molecular hybridization approach incorporating the carbamate fragment and tryptamine skeleton.

**FIGURE 1:**
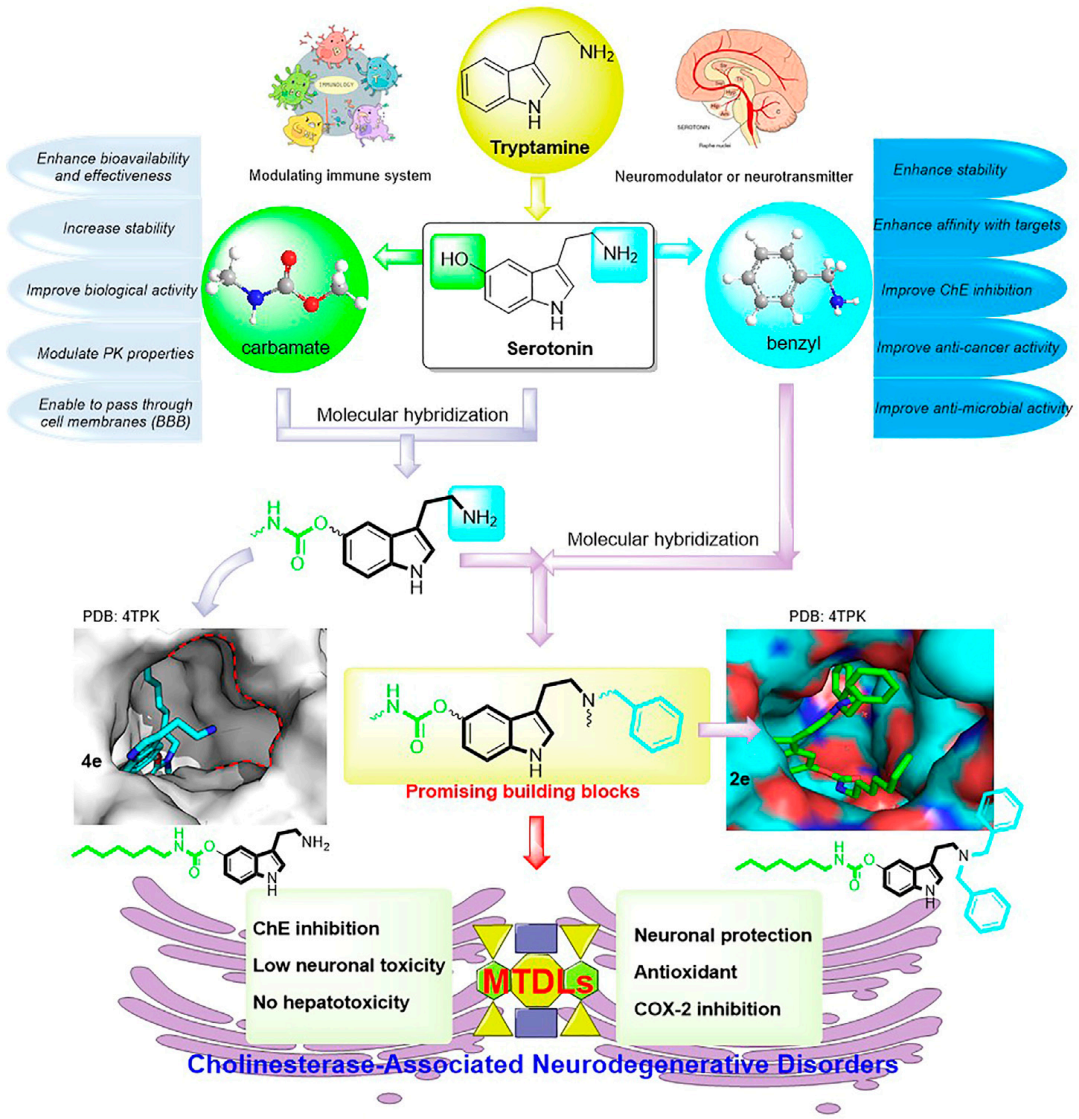
Design strategy for developing promising building blocks for MTDLs against ChE-associated neurodegenerative disorders.

In this context and combined with the previous studies (Darras et al., 2012; Huang et al., 2014; Sawatzky et al., 2016; Scheiner et al., 2022), we first synthesized and evaluated the potential bioactivities of CDTs. Among them, **4e** possessed highly selective BuChE inhibitory activity (Scheme 1C). Further molecular docking assay revealed that the amino group is oriented toward two unacted subpockets (Figure 1). To further improve its BuChE inhibition potency, we decide to further introduce an additional auxiliary fragment. Based on surveying extensive pieces of the literature, we find that the benzyl group is often involved in the design of pharmaceutical molecules to improve the stability and the biological activity of intrinsic compounds, which are also widely distributed in the structure of natural products (Scheme 1C) (Cacabelos, 2007; Guo et al., 2018; Jiang et al., 2019; Sang et al., 2020; Choubey et al., 2021; Saeedi et al., 2021; Zhu et al., 2021; Yang et al., 2022). Enlightened by this, benzyl-substituted CTDs were subsequently obtained, and the molecular docking assay confirmed that the benzyl group could interact with the two active subpockets (Figure 1). To develop potent benzyl-substituted CTDs for the discovery of multifunctional drugs against refractory diseases, herein we designed and synthesized a series of CTDs bearing benzyl groups and focused on their action modes of ChE inhibition and kinetic characteristics of interaction with BuChE (Figure 1). We also evaluated *in vitro* neuronal protection effects, antioxidant activities, and COX-2 inhibitory effects of the selected compounds with little neuronal toxicity and hepatotoxicity.

## Experimental section

### Chemistry

All reagents were used without further purification and bought from common commercial suppliers. Proton (^1^H) and carbon (^13^C) NMR spectra (400 or 300 MHz for ^1^HNMR; 101 or 75 MHz for ^13^CNMR) were recorded on a Bruker spectrometer (Bruker Company, Germany). NMR spectra used DMSO-*d*_*6*_, MeOD, or CDCl_3_ as a solvent. Proton chemical shifts are reported relative to a residual solvent peak (CDCl_3_ at 7.26 ppm, DMSO-*d*_*6*_ at 2.50 ppm, MeOD at 3.31 ppm). Carbon chemical shifts are reported relative to a residual solvent peak (CDCl_3_ at 77.00 ppm, DMSO-*d*_*6*_ at 39.60 ppm, MeOD at 49.00 ppm). The values of the chemical shifts are expressed in ppm and the coupling constants (*J*) in hertz. The following abbreviations were used to designate multiplicities: *s* = singlet, *d* = doublet, *t* = triplet, *q* = quartet, and *m* = multiplet. High-resolution mass spectrometry (HRMS) was obtained on an Agilent UPLC-IM-QTOF spectrometer (Agilent 6560, United States). Mass spectrometry (MS) analysis was performed using a liquid chromatograph-mass spectrometer (LC-MS). The purity was determined by high-performance liquid chromatography (HPLC). The purity of all final compounds was more than 95% (Agilent 1260 Infinity II; United States). The column was Eclipse Plus C18 (4.6 × 150 mm, 4 µm). Chromatographic conditions for all compounds: Mobile phase: 0–7 min, MeOH:H_2_O = 20:80; 7–16 min MeOH:H_2_O = 95:5, 16–25 min MeOH:H_2_O = 20:80; wavelength: 254 nm; column temperature: 25°C; flow rate of 0.5 ml/min. All synthesized compounds are >95% pure by HPLC analysis.

***2a***: 3-(2-(dibenzylamino)ethyl)-1H-indol-5-yl propylcarbamate: white solid (68% yield). ^1^H NMR (400 MHz, CDCl_3_-*d*) *δ* 7.88 (s, 1H, NH), 7.25 (d, *J* = 7.6 Hz, 4H, aromatic protons), 7.20 – 7.14 (m, 4H, aromatic protons), 7.09 (m, 3H, aromatic protons), 6.97 – 6.90 (m, 2H, aromatic protons), 6.72 (m, 1H, aromatic proton), 6.57 (s, 1H, CH), 3.53 (s, 4H, CH_2_), 3.11 (q, *J* = 6.7 Hz, 2H, CH_2_), 2.74 (m, 2H, CH_2_), 2.61 (m, 2H, CH_2_), 1.46 (m, 2H, CH_2_), 0.84 (t, *J* = 7.4 Hz, 3H, CH_3_). ^13^C NMR (75 MHz, CDCl_3_) *δ* 156.15, 144.36, 140.14, 134.15, 129.09, 128.45, 128.04, 127.05, 123.16, 116.37, 111.63, 111.28, 58.53, 54.01, 43.28, 23.43, 23.18, 11.57. MS (ESI^+^) m/z calcd for C_28_H_32_N_3_O_2_^+^ [M+H]^+^ 442.2, found 442.2. Purity = 96.4%.

***2b***: 3-(2-(dibenzylamino)ethyl)-1H-indol-5-yl butylcarbamate: orange solid (70% yield). ^1^H NMR (400 MHz, CDCl_3_-*d*) *δ* 7.89 (s, 1H, NH), 7.36 – 7.30 (m, 4H, aromatic protons), 7.27 – 7.21 (m, 4H, aromatic protons), 7.18 (m, 1H, aromatic proton), 7.16 (d, *J* = 2.3 Hz, 1H, aromatic proton), 7.15 – 7.10 (m, 1H, aromatic proton), 7.02 (d, *J* = 2.3 Hz, 1H, aromatic proton), 6.83 (m, 1H, aromatic proton), 6.75 (d, *J* = 2.3 Hz, 1H, CH), 3.61 (s, 4H, CH_2_), 3.23 (q, *J* = 6.7 Hz, 2H, CH_2_), 2.84 (m, 2H, CH_2_), 2.73 – 2.67 (m, 2H, CH_2_), 1.56 – 1.46 (m, 2H, CH_2_), 1.41 – 1.29 (m, 2H, CH_2_), 0.91 (t, *J* = 7.3 Hz, 3H, CH_3_). ^13^C NMR (101 MHz, CDCl_3_) *δ* 155.73, 144.26, 139.90, 133.89, 128.94, 128.83, 128.24, 128.19, 127.88, 126.79, 122.77, 116.30, 114.73, 111.27, 111.15, 58.31, 53.76, 41.04, 32.04, 22.97, 19.98, 13.80. MS (ESI^+^) m/z calcd for C_29_H_34_N_3_O_2_^+^ [M+H]^+^ 456.3, found 456.3. Purity = 96.2%.

***2c***: 3-(2-(dibenzylamino)ethyl)-1H-indol-5-yl pentylcarbamate: orange solid (63% yield). ^1^H NMR (400 MHz, CDCl_3_-*d*) *δ* 7.99 (s, 1H, NH), 7.36 (d, *J* = 7.1 Hz, 4H, aromatic protons), 7.28 (t, *J* = 7.4 Hz, 4H, aromatic protons), 7.22 (s, 1H, aromatic proton), 7.20 (d, *J* = 7.2 Hz, 2H, aromatic protons), 7.08 (d, *J* = 8.7 Hz, 1H, aromatic proton), 7.05 (d, *J* = 2.1 Hz, 1H, aromatic proton), 6.84 (m, 1H, aromatic proton), 6.69 (s, 1H, CH), 3.65 (s, 4H, CH_2_), 3.25 (q, *J* = 6.8 Hz, 2H, CH_2_), 2.89 – 2.82 (m, 2H, CH_2_), 2.72 (m, 2H, CH_2_), 1.57 (q, *J* = 7.0 Hz, 2H, CH_2_), 1.34 (m, 4H, CH_2_), 0.91 (t, *J* = 6.7 Hz, 3H, CH_3_). ^13^C NMR (101 MHz, CDCl_3_) *δ* 155.82, 144.13, 139.87, 133.88, 128.90, 128.81, 128.21, 128.17, 127.78, 126.76, 122.85, 116.13, 114.41, 111.33, 111.02, 58.26, 53.74, 41.30, 29.59, 28.93, 22.90, 22.36, 14.02. MS (ESI^+^) m/z calcd for C_30_H_36_N_3_O_2_^+^ [M+H]^+^ 470.3, found 470.3. Purity = 97.9%.

***2d***: 3-(2-(dibenzylamino)ethyl)-1H-indol-5-yl hexylcarbamate: brown solid (67% yield). ^1^H NMR (400 MHz, CDCl_3_-*d*) *δ* 7.93 (s, 1H, NH), 7.28 (s, 2H, aromatic protons), 7.19 (t, *J* = 7.4 Hz, 4H, aromatic protons), 7.11 (m, 3H, aromatic protons), 7.00 – 6.93 (m, 2H, aromatic protons), 6.74 (m, 1H, aromatic proton), 6.58 (s, 1H, CH), 3.55 (s, 4H, CH_2_), 3.16 (q, *J* = 6.8 Hz, 2H, CH_2_), 2.76 (m, 2H, CH_2_), 2.63 (m, 2H, CH_2_), 1.46 (t, *J* = 7.3 Hz, 2H, CH_2_), 1.29 – 1.17 (m, 6H, CH_2_), 0.83 – 0.77 (m, 3H, CH_3_). ^13^C NMR (101 MHz, CDCl_3_) *δ* 155.83, 144.11, 139.84, 133.88, 128.90, 128.81, 128.17, 127.77, 126.77, 122.87, 116.10, 114.35, 111.34, 111.00, 58.25, 53.73, 41.33, 31.47, 29.86, 26.45, 22.88, 22.57, 14.04. MS (ESI^+^) m/z calcd for C_31_H_38_N_3_O_2_^+^ [M+H]^+^ 484.3, found 484.3. Purity = 95.4%.

***2e***: 3-(2-(dibenzylamino)ethyl)-1H-indol-5-yl heptylcarbamate: white solid (65% yield). ^1^H NMR (300 MHz, CDCl_3_-*d*) *δ* 7.87 (s, 1H, NH), 7.29 (d, *J* = 2.1 Hz, 2H, aromatic protons), 7.19 (t, *J* = 7.3 Hz, 5H, aromatic protons), 7.12 (m, 2H, aromatic protons), 7.03 (d, *J* = 8.5 Hz, 1H, aromatic proton), 6.96 (d, *J* = 2.4 Hz, 1H, aromatic proton), 6.76 (m, 1H, aromatic proton), 6.65 (d, *J* = 2.3 Hz, 1H, CH), 3.56 (s, 4H, CH_2_), 3.17 (q, *J* = 6.7 Hz, 2H, CH_2_), 2.77 (m, 2H, CH_2_), 2.64 (m, 2H, CH_2_), 1.47 (d, *J* = 8.7 Hz, 2H, CH_2_), 1.34 – 1.11 (m, 8H, CH_2_), 0.87 – 0.72 (m, 3H, CH_3_). ^13^C NMR (101 MHz, CDCl_3_) *δ* 155.78, 144.19, 139.89, 133.89, 128.92, 128.82, 128.23, 128.18, 126.78, 122.82, 116.21, 114.56, 111.31, 111.08, 58.39, 58.29, 53.75, 41.35, 31.78, 29.93, 28.98, 26.77, 22.94, 22.62, 14.11. MS (ESI^+^) m/z calcd for C_32_H_40_N_3_O_2_^+^ [M+H]^+^ 498.3, found 498.3. Purity = 96.4%.

***2f***: 3-(2-(dibenzylamino)ethyl)-1H-indol-5-yl cyclopropylcarbamate: white solid (70% yield). ^1^H NMR (400 MHz, CDCl_3_-*d*) *δ* 7.97 (s, 1H, NH), 7.39 – 7.34 (m, 4H, aromatic protons), 7.32 – 7.25 (m, 4H, aromatic protons), 7.25 – 7.22 (m, 1H, aromatic proton), 7.22 – 7.17 (m, 2H, aromatic protons), 7.13 (d, *J* = 8.7 Hz, 1H, aromatic proton), 7.06 (d, *J* = 2.2 Hz, 1H, aromatic proton), 6.88 – 6.82 (m, 1H, aromatic proton), 6.75 (d, *J* = 2.3 Hz, 1H, CH), 3.66 (s, 4H, CH_2_), 2.87 (m, 2H, CH_2_), 2.79 – 2.66 (m, 3H, CH_2_, CH), 0.85 – 0.71 (m, 2H, CH_2_), 0.69 – 0.53 (m, 2H, CH_2_). ^13^C NMR (101 MHz, CDCl_3_) *δ* 156.38, 144.12, 139.93, 133.94, 128.84, 128.21, 127.85, 126.87, 126.80, 122.87, 116.17, 114.61, 111.33, 111.08, 58.32, 53.79, 23.37, 22.98, 6.93. MS (ESI^+^) m/z calcd for C_28_H_30_N_3_O_2_^+^ [M+H]^+^ 440.2, found 440.2. Purity = 97.4%.

***2g***: 3-(2-(dibenzylamino)ethyl)-1H-indol-5-yl cyclopentylcarbamate: white solid (69% yield). ^1^H NMR (300 MHz, CDCl_3_-*d*) *δ* 7.93 (s, 1H, NH), 7.34 (d, *J* = 7.1 Hz, 4H, aromatic protons), 7.26 (t, *J* = 7.3 Hz, 4H, aromatic protons), 7.19 (d, *J* = 6.7 Hz, 2H, aromatic protons), 7.10 (d, *J* = 8.5 Hz, 1H, aromatic proton), 7.03 (d, *J* = 2.2 Hz, 1H, aromatic proton), 6.87 – 6.79 (m, 1H, aromatic proton), 6.73 (s, 1H, CH), 4.94 (d, *J* = 7.3 Hz, 1H, CH), 3.63 (s, 4H, CH_2_), 2.91 – 2.79 (m, 2H, CH_2_), 2.71 (m, 2H, CH_2_), 2.07 – 1.93 (m, 2H, CH_2_), 1.76 – 1.55 (m, 4H, CH_2_), 1.47 (m, 2H). ^13^C NMR (75 MHz, CDCl_3_) *δ* 144.39, 140.11, 134.10, 129.05, 128.41, 128.06, 127.01, 123.01, 116.49, 114.80, 111.51, 111.34, 58.51, 53.97, 53.24, 33.51, 23.82, 23.17. MS (ESI^+^) m/z calcd for C_30_H_34_N_3_O_2_^+^ [M+H]^+^ 468.3, found 468.3. Purity = 96.4%.

***2h***: 3-(2-(dibenzylamino)ethyl)-1H-indol-5-yl cyclohexylcarbamate: bright solid (61% yield). ^1^H NMR (300 MHz, CDCl_3_-*d*) *δ* 8.05 (s, 1H, NH), 7.43 (d, *J* = 6.5 Hz, 4H, aromatic protons), 7.35 (t, *J* = 7.4 Hz, 4H, aromatic protons), 7.27 (m, 2H, aromatic protons), 7.15 – 7.08 (m, 2H, aromatic protons), 6.90 (m, 1H, aromatic proton), 6.75 (m, 1H, CH), 5.00 (d, *J* = 8.1 Hz, 1H, CH), 3.71 (s, 4H, CH_2_), 2.91 (m, 2H, CH_2_), 2.78 (m, 2H, CH_2_), 2.07 (d, *J* = 10.8 Hz, 2H, CH_2_), 1.72 (m, 3H, CH_2_, CH), 1.50 – 1.35 (m, 2H, CH_2_), 1.26 (q, *J* = 11.6 Hz, 3H, CH_3_). ^13^C NMR (75 MHz, CDCl_3_) *δ* 154.95, 144.05, 139.85, 133.83, 128.88, 128.79, 128.16, 127.73, 126.75, 122.82, 116.13, 114.32, 111.31, 111.01, 58.31, 58.22, 53.70, 50.08, 33.32, 25.46, 24.78, 22.87. MS (ESI^+^) m/z calcd for C_31_H_36_N_3_O_2_^+^ [M+H]^+^ 482.3, found 482.3. Purity = 96.1%.

***2i***: 3-(2-(dibenzylamino)ethyl)-1H-indol-5-yl phenylcarbamate: orange solid (66% yield). ^1^H NMR (400 MHz, CDCl_3_-*d*) *δ* 7.95 (s, 1H, NH), 7.44 (d, *J* = 8.0 Hz, 2H, aromatic protons), 7.35 (d, *J* = 7.5 Hz, 4H, aromatic protons), 7.30 (s, 1H, aromatic proton), 7.25 (d, *J* = 7.4 Hz, 3H, aromatic protons), 7.18 (q, *J* = 8.3, 6.7 Hz, 3H, aromatic protons), 7.13 – 7.08 (m, 1H, aromatic proton), 7.07 – 7.04 (m, 2H, aromatic protons), 7.03 – 7.00 (m, 1H, aromatic proton), 6.89 (m, 1H, aromatic proton), 6.76 (s, 1H, CH), 3.64 (s, 4H, CH_2_), 2.86 (m, 2H, CH_2_), 2.73 (m, 2H, CH_2_). ^13^C NMR (101 MHz, CDCl_3_) *δ* 143.60, 139.79, 137.66, 134.01, 129.10, 128.76, 128.14, 127.81, 126.75, 123.67, 122.93, 118.63, 115.99, 114.60, 111.36, 111.15, 58.25, 53.62, 22.90. MS (ESI^+^) m/z calcd for C_31_H_30_N_3_O_2_^+^ [M+H]^+^ 476.2, found 476.2. Purity = 95.2%.

***2j***: 3-(2-(dibenzylamino)ethyl)-1H-indol-5-yl dimethylcarbamate: white solid (64% yield). ^1^H NMR (400 MHz, CDCl_3_-*d*) *δ* 7.91 (s, 1H, NH), 7.42 – 7.36 (m, 4H, aromatic protons), 7.30 (t, *J* = 7.5 Hz, 4H, aromatic protons), 7.25 – 7.19 (m, 3H, aromatic protons), 7.06 (d, *J* = 2.2 Hz, 1H, aromatic proton), 6.89 (m, 1H, aromatic proton), 6.85 (d, *J* = 2.3 Hz, 1H, CH), 3.68 (s, 4H, CH_2_), 3.15 (s, 3H, CH_3_), 3.05 (s, 3H, CH_3_), 2.91 (m, 2H, CH_2_), 2.78 (m, 2H, CH_2_). ^13^C NMR (75 MHz, CDCl_3_) *δ* 156.44, 144.81, 140.20, 134.13, 129.08, 128.44, 128.03, 127.01, 123.09, 116.49, 114.57, 111.57, 111.32, 58.60, 58.51, 54.02, 37.02, 36.74, 23.20. MS (ESI^+^) m/z calcd for C_27_H_30_N_3_O_2_^+^ [M+H]^+^ 428.2, found 428.2. Purity = 95.8%.

***2k***: 3-(2-(dibenzylamino)ethyl)-1H-indol-5-yl diethylcarbamate: orange solid (66% yield). ^1^H NMR (300 MHz, CDCl_3_-*d*) *δ* 8.03 (s, 1H, NH), 7.40 (d, *J* = 7.1 Hz, 4H, aromatic protons), 7.32 (t, *J* = 7.3 Hz, 4H, aromatic protons), 7.27 – 7.21 (m, 2H, aromatic protons), 7.14 (d, *J* = 8.7 Hz, 1H, aromatic proton), 7.08 (d, *J* = 2.2 Hz, 1H, aromatic proton), 6.87 (m, 1H, aromatic proton), 6.75 (d, *J* = 2.2 Hz, 1H, CH), 3.69 (s, 4H, CH_2_), 3.48 (q, *J* = 8.0 Hz, 4H, CH_2_), 2.91 (m, 2H, CH_2_), 2.77 (m, 2H, CH_2_), 1.35 – 1.22 (m, 6H, CH_3_). ^13^C NMR (75 MHz, CDCl_3_) *δ* 155.34, 144.54, 139.83, 133.75, 128.76, 128.11, 127.75, 126.71, 122.64, 116.30, 114.36, 111.16, 111.01, 58.18, 53.66, 41.80, 22.84, 14.28, 13.42. MS (ESI^+^) m/z calcd for C_29_H_34_N_3_O_2_^+^ [M+H]^+^ 456.3, found 456.3. Purity = 95.5%.

***2l***: 3-(2-(dibenzylamino)ethyl)-1H-indol-5-yl dipropylcarbamate: orange solid (61% yield). ^1^H NMR (300 MHz, CDCl_3_-*d*) *δ* 9.39 (s, 1H, NH), 8.74 (t, *J* = 5.0 Hz, 4H, aromatic protons), 8.66 (m, 4H, aromatic protons), 8.58 (m, 2H, aromatic protons), 8.44 (m, 2H, aromatic protons), 8.19 (q, *J* = 3.6 Hz, 1H, aromatic proton), 8.06 (s, 1H, CH), 5.03 (d, *J* = 3.7 Hz, 4H, CH_2_), 4.70 (m, 4H, CH_2_), 4.32 – 4.18 (m, 2H, CH_2_), 4.11 (m, 2H, CH_2_), 3.06 (s, 4H, CH_2_), 2.35 (m, 6H, CH_3_). ^13^C NMR (101 MHz, CDCl_3_) *δ* 155.94, 144.67, 139.97, 133.85, 128.95, 128.85, 128.21, 127.83, 126.79, 122.75, 116.32, 114.42, 111.27, 111.02, 58.29, 53.80, 49.58, 49.27, 22.93, 22.13, 21.39, 11.40. MS (ESI^+^) m/z calcd for C_31_H_38_N_3_O_2_^+^ [M+H]^+^ 484.3, found 484.3. Purity = 96.5%.

***2m***: 3-(2-(dibenzylamino)ethyl)-1H-indol-5-yl dibutylcarbamate: orange solid (60% yield). ^1^H NMR (300 MHz, CDCl_3_-*d*) *δ* 9.27 (s, 1H, NH), 8.67 (d, *J* = 7.3 Hz, 4H, aromatic protons), 8.58 (t, *J* = 7.3 Hz, 4H, aromatic protons), 8.51 (d, *J* = 7.5 Hz, 2H, aromatic protons), 8.43 (d, *J* = 8.7 Hz, 1H, aromatic proton), 8.35 (d, *J* = 2.3 Hz, 1H, aromatic proton), 8.13 (d, *J* = 8.6 Hz, 1H, aromatic proton), 8.03 (s, 1H, CH), 4.96 (m, 4H, CH_2_), 4.67 (m, 4H, CH_2_), 4.17 (d, *J* = 7.9 Hz, 2H, CH_2_), 4.10 – 3.98 (m, 2H, CH_2_), 2.94 (m, 4H, CH_2_), 2.69 (q, *J* = 9.8, 8.3 Hz, 4H, CH_2_), 2.26 (q, *J* = 7.3 Hz, 6H, CH_3_). ^13^C NMR (101 MHz, CDCl_3_) *δ* 155.80, 144.73, 139.94, 133.81, 128.83, 128.19, 127.86, 126.77, 122.66, 116.38, 114.55, 111.20, 111.07, 58.28, 53.78, 47.57, 47.23, 31.04, 30.28, 22.93, 20.14, 13.96. MS (ESI^+^) m/z calcd for C_33_H_42_N_3_O_2_^+^ [M+H]^+^ 512.3, found 512.3. Purity = 98.1%.

***2n:*** 3-(2-(dibenzylamino)ethyl)-1H-indol-5-yl diphenylcarbamate: orange solid (67% yield). ^1^H NMR (300 MHz, CDCl_3_-*d*) *δ* 7.90 (s, 1H, NH), 7.41 – 7.32 (m, 12H, aromatic protons), 7.29 (d, *J* = 1.3 Hz, 1H, aromatic proton), 7.28 – 7.23 (m, 4H, aromatic protons), 7.23 – 7.15 (m, 3H, aromatic protons), 7.12 (d, *J* = 8.6 Hz, 1H, aromatic proton), 7.09 (d, *J* = 2.3 Hz, 1H, aromatic proton), 6.88 (m, 1H, aromatic proton), 6.73 (d, *J* = 2.3 Hz, 1H, CH), 3.65 (s, 4H, CH_2_), 2.87 (m, 2H, CH_2_), 2.73 (m, 2H, CH_2_). ^13^C NMR (75 MHz, CDCl_3_) *δ* 154.09, 144.23, 142.53, 139.82, 133.84, 128.95, 128.74, 128.12, 127.71, 126.91, 126.73, 126.23, 122.74, 115.87, 114.58, 111.16, 110.87, 58.21, 53.63, 22.88. MS (ESI^+^) m/z calcd for C_37_H_34_N_3_O_2_^+^ [M+H]^+^ 552.3, found 552.3. Purity = 98.9%.

***2o***: 3-(2-(dibenzylamino)ethyl)-1H-indol-5-yl methoxy(methyl)carbamate: brown solid (65% yield). ^1^H NMR (300 MHz, CDCl_3_-*d*) *δ* 7.96 (s, 1H, NH), 7.38 (d, *J* = 7.4 Hz, 4H, aromatic protons), 7.29 (m, 4H, aromatic protons), 7.25 – 7.16 (m, 3H, aromatic protons), 7.09 (s, 1H, aromatic proton), 6.90 (d, *J* = 8.3 Hz, 1H, aromatic proton), 6.82 (s, 1H, CH), 3.84 (d, *J* = 2.4 Hz, 3H, CH_3_), 3.68 (s, 4H, CH_2_), 3.33 (d, *J* = 2.4 Hz, 3H, CH_3_), 2.95 – 2.85 (m, 2H, CH_2_), 2.77 (d, *J* = 7.9 Hz, 2H, CH_2_). ^13^C NMR (101 MHz, CDCl_3_) *δ* 156.32, 144.08, 139.83, 133.98, 128.79, 128.15, 127.82, 126.76, 122.86, 115.99, 114.69, 111.28, 111.03, 61.78, 58.26, 53.68, 35.77, 22.93. HRMS (ESI^+^) m/z calcd for C_27_H_30_N_3_O_3_^+^ [M+H]^+^ 444.2282, found 444.2307. Purity = 96.7%.

***2p***: 3-(2-(dibenzylamino)ethyl)-1H-indol-5-yl methyl(phenyl)carbamate: orange solid (70% yield). ^1^H NMR (400 MHz, CDCl_3_-*d*) *δ* 7.91 (s, 1H, NH), 7.42 (d, *J* = 4.3 Hz, 4H, aromatic protons), 7.38 (d, *J* = 7.5 Hz, 4H, aromatic protons), 7.29 (t, *J* = 7.4 Hz, 5H, aromatic protons), 7.21 (m, 3H, aromatic protons), 7.07 (s, 1H, aromatic proton), 6.89 (d, *J* = 8.7 Hz, 1H, aromatic proton), 6.84 (s, 1H, CH), 3.68 (s, 4H, CH_2_), 3.48 (m, 3H, CH_3_), 2.91 (m, 2H, CH_2_), 2.77 (m, 2H, CH_2_). ^13^C NMR (101 MHz, CDCl_3_) *δ* 154.97, 144.54, 143.25, 133.85, 128.94, 128.82, 128.16, 127.80, 126.79, 125.78, 122.69, 116.22, 111.13, 111.06, 58.23, 53.63, 38.13, 22.89. HRMS (ESI^+^) m/z calcd for C_32_H_32_N_3_O_2_^+^ [M+H]^+^ 490.2489, found 490.2508. Purity = 96.9%.

***2q***: 3-(2-(dibenzylamino)ethyl)-1H-indol-5-yl azetidine-1-carboxylate: brown solid (50% yield). ^1^H NMR (400 MHz, CDCl_3_-*d*) *δ* 7.96 (s, 1H, NH), 7.37 (d, *J* = 7.0 Hz, 4H, aromatic protons), 7.29 (t, *J* = 7.5 Hz, 4H, aromatic protons), 7.24 – 7.19 (m, 2H, aromatic protons), 7.14 (m, 1H, aromatic proton), 7.06 (d, *J* = 2.3 Hz, 1H, aromatic proton), 6.86 (m, 1H, aromatic proton), 6.77 (d, *J* = 2.7 Hz, 1H, CH), 4.35 – 4.03 (m, 4H, CH_2_), 3.66 (s, 4H, CH_2_), 2.88 (m, 2H, CH_2_), 2.79 – 2.71 (m, 2H, CH_2_), 2.40 – 2.27 (m, 2H, CH_2_). ^13^C NMR (101 MHz, CDCl_3_) *δ* 155.53, 144.15, 139.86, 133.83, 128.78, 128.13, 127.80, 126.71, 122.70, 116.19, 114.52, 111.19, 111.02, 58.22, 53.74, 22.92, 15.79. HRMS (ESI^+^) m/z calcd for C_28_H_30_N_3_O_2_^+^ [M+H]^+^ 440.2333, found 440.2332. Purity = 95.3%.

***2r***: 3-(2-(dibenzylamino)ethyl)-1H-indol-5-yl pyrrolidine-1-carboxylate: brown solid (53% yield). ^1^H NMR (300 MHz, CDCl_3_-*d*) *δ* 7.96 (s, 1H, NH), 7.42 – 7.34 (m, 4H, aromatic protons), 7.33 – 7.26 (m, 4H, aromatic protons), 7.23 (m, 1H, aromatic proton), 7.20 (d, *J* = 2.7 Hz, 1H, aromatic proton), 7.18 – 7.12 (m, 1H, aromatic proton), 7.07 (d, *J* = 2.2 Hz, 1H, aromatic proton), 6.88 (m, 1H, aromatic proton), 6.77 (d, *J* = 2.3 Hz, 1H, CH), 3.66 (s, 4H, CH_2_), 3.60 (t, *J* = 6.5 Hz, 2H, CH_2_), 3.51 (t, *J* = 6.5 Hz, 2H, CH_2_), 2.89 (dd, *J* = 9.4, 5.8 Hz, 2H, CH_2_), 2.81 – 2.70 (m, 2H, CH_2_), 2.05 – 1.87 (m, 4H, CH_2_). ^13^C NMR (75 MHz, CDCl_3_) *δ* 154.32, 144.49, 139.86, 133.77, 128.76, 128.11, 127.78, 126.68, 122.61, 116.39, 114.50, 111.13, 58.19, 53.71, 46.41, 46.30, 25.87, 25.03, 22.91. MS (ESI^+^) m/z calcd for C_29_H_32_N_3_O_2_^+^ [M+H]^+^ 454.2, found 454.2. Purity = 96.0%.

***2s***: 3-(2-(dibenzylamino)ethyl)-1H-indol-5-yl piperidine-1-carboxylate: brown solid (57% yield). ^1^H NMR (300 MHz, CDCl_3_-*d*) *δ* 8.02 (s, 1H, NH), 7.37 (d, *J* = 7.1 Hz, 4H, aromatic protons), 7.33 – 7.25 (m, 4H, aromatic protons), 7.20 (m, *J* = 6.1, 1.7 Hz, 2H, aromatic protons), 7.08 (d, *J* = 8.8 Hz, 1H, aromatic proton), 7.03 (d, *J* = 2.3 Hz, 1H, aromatic proton), 6.83 (dd, *J* = 8.6, 2.3 Hz, 1H, aromatic proton), 6.68 (d, *J* = 2.3 Hz, 1H, CH), 3.65 (s, 6H, CH_2_), 3.55 (s, 2H, CH_2_), 2.86 (dd, *J* = 9.6, 5.7 Hz, 2H, CH_2_), 2.72 (dd, *J* = 10.2, 5.9 Hz, 2H, CH_2_), 1.65 (s, 6H, CH_2_). ^13^C NMR (75 MHz, CDCl_3_) *δ* 154.91, 144.50, 139.79, 133.75, 128.75, 128.11, 127.70, 126.70, 122.69, 116.21, 114.22, 111.20, 110.97, 58.14, 53.65, 45.34, 45.02, 25.86, 25.58, 24.34, 22.82. MS (ESI^+^) m/z calcd for C_30_H_34_N_3_O_2_^+^ [M+H]^+^ 468.3, found 468.3. Purity = 96.1%.

***2t***: 3-(2-(dibenzylamino)ethyl)-1H-indol-5-yl azepane-1-carboxylate: orange solid (58% yield). ^1^H NMR (400 MHz, CDCl_3_-*d*) *δ* 7.97 – 7.90 (m, 1H, NH), 7.32 (d, *J* = 7.1 Hz, 4H, aromatic protons), 7.24 (m, 4H, aromatic protons), 7.18 (t, *J* = 1.8 Hz, 1H, aromatic protons), 7.16 – 7.13 (m, 1H, aromatic protons), 7.07 (d, *J* = 8.7 Hz, 1H, aromatic proton), 7.01 (d, *J* = 2.2 Hz, 1H, aromatic protons), 6.80 (dd, *J* = 8.7, 2.3 Hz, 1H, aromatic protons), 6.68 (d, *J* = 2.3 Hz, 1H, CH), 3.61 (s, 4H, CH_2_), 3.59 – 3.54 (m, 2H, CH_2_), 3.50 (t, *J* = 6.1 Hz, 2H, CH_2_), 2.83 (m, 2H, CH_2_), 2.70 (m, 2H, CH_2_), 1.84 – 1.68 (m, 4H, CH_2_), 1.60 (m, 4H, CH_2_). ^13^C NMR (101 MHz, CDCl_3_) *δ* 155.82, 144.72, 139.94, 133.84, 128.84, 128.19, 127.86, 126.78, 122.70, 116.42, 114.51, 111.24, 111.09, 58.28, 53.79, 47.39, 47.15, 28.82, 28.24, 27.59, 27.03, 22.95. MS (ESI^+^) m/z calcd for C_31_H_36_N_3_O_2_^+^ [M+H]^+^ 482.3, found 482.3. Purity = 96.3%.

***2u***: 3-(2-(dibenzylamino)ethyl)-1H-indol-5-yl morpholine-4-carboxylate: white solid (59% yield). ^1^H NMR (400 MHz, CDCl_3_-*d*) *δ* 7.98 (s, 1H, NH), 7.36 (d, *J* = 7.0 Hz, 4H, aromatic protons), 7.28 (t, *J* = 7.5 Hz, 4H, aromatic protons), 7.22 (m, 1H, aromatic proton), 7.21 – 7.18 (m, 1H, aromatic proton), 7.16 – 7.11 (m, 2H, aromatic proton), 7.05 (d, *J* = 2.3 Hz, 1H, aromatic proton), 6.85 (dd, *J* = 8.7, 2.3 Hz, 1H, aromatic proton), 6.73 (d, *J* = 2.2 Hz, 1H, CH), 3.76 (m, 4H, CH_2_), 3.72 – 3.54 (m, 8H, CH_2_), 2.87 (m, 2H, CH_2_), 2.74 (m, 2H, CH_2_). ^13^C NMR (101 MHz, CDCl_3_) *δ* 154.95, 144.37, 139.91, 133.97, 128.85, 128.57, 128.22, 127.90, 126.80, 122.94, 116.14, 114.57, 111.37, 111.08, 66.73, 58.29, 53.79, 44.88, 44.23, 22.98. MS (ESI^+^) m/z calcd for C_29_H_30_N_3_O_3_^+^ [M+H]^+^ 470.2, found 470.2. Purity = 95.3%.

***3a:*** 3-(2-(benzylamino)ethyl)-1H-indol-5-yl propylcarbamate: white solid (85% yield). ^1^H NMR (400 MHz, MeOD) *δ* 7.42 – 7.34 (m, 5H, aromatic protons), 7.32 (d, *J* = 8.7 Hz, 1H, NH), 7.25 (d, *J* = 2.2 Hz, 1H, aromatic proton), 7.14 (s, 1H, aromatic proton), 6.86 (dd, *J* = 8.7, 2.3 Hz, 1H, CH), 4.00 (s, 2H, CH_2_), 3.15 (t, *J* = 7.1 Hz, 2H, CH_2_), 3.11 (m, 2H, CH_2_), 3.07 – 3.00 (m, 2H, CH_2_), 1.59 (m, 2H, CH_2_), 0.97 (t, *J* = 7.4 Hz, 3H, CH_3_). ^13^C NMR (101 MHz, MeOD) *δ* 158.31, 145.49, 135.63, 135.02, 130.27, 129.78, 129.59, 128.24, 125.29, 117.05, 112.55, 111.37, 52.67, 49.06, 43.60, 23.90, 23.85, 11.42. MS (ESI^+^) m/z calcd for C_21_H_26_N_3_O_2_^+^ [M+H]^+^ 352.2, found 352.2. Purity = 95.1%.

***3b***: 3-(2-(benzylamino)ethyl)-1H-indol-5-yl butylcarbamate: white solid (88% yield). ^1^H NMR (400 MHz, MeOD) *δ* 7.46 (m, *J* = 10.9, 3.9, 2.1 Hz, 5H, aromatic protons), 7.34 (d, *J* = 8.7 Hz, 1H, NH), 7.27 (d, *J* = 2.2 Hz, 1H, aromatic proton), 7.21 (s, 1H, aromatic proton), 6.87 (dd, *J* = 8.7, 2.2 Hz, 1H, CH), 4.16 (s, 2H, CH_2_), 3.27 (t, *J* = 7.7 Hz, 2H, CH_2_), 3.20 (t, *J* = 7.0 Hz, 2H, CH_2_), 3.15 – 3.08 (m, 2H, CH_2_), 1.61 – 1.50 (m, 2H, CH_2_), 1.42 (m, 2H, CH_2_), 0.97 (t, *J* = 7.3 Hz, 3H, CH_3_). ^13^C NMR (101 MHz, MeOD) *δ* 158.28, 145.59, 135.66, 132.47, 130.79, 130.41, 130.05, 128.08, 125.58, 117.20, 112.67, 111.32, 110.30, 52.04, 48.62, 41.54, 32.81, 22.99, 20.79, 13.93. MS (ESI^+^) m/z calcd for C_22_H_28_N_3_O_2_^+^ [M+H]^+^ 366.2, found 366.2. Purity = 95.7%.

***3c***: 3-(2-(benzylamino)ethyl)-1H-indol-5-yl pentylcarbamate: white solid (90% yield). ^1^H NMR (400 MHz, MeOD) *δ* 7.50 (m, *J* = 7.2, 3.6 Hz, 5H, aromatic protons), 7.38 (d, *J* = 8.6 Hz, 1H, NH), 7.30 (d, *J* = 2.3 Hz, 1H, aromatic proton), 7.24 (s, 1H, aromatic proton), 6.91 (dd, *J* = 8.7, 2.3 Hz, 1H, CH), 4.21 (s, 2H, CH_2_), 3.34 (s, 2H, CH_2_), 3.22 (t, *J* = 7.1 Hz, 2H, CH_2_), 3.16 (t, *J* = 7.7 Hz, 2H, CH_2_), 1.61 (t, *J* = 7.1 Hz, 2H, CH _2_), 1.52 – 1.32 (m, 4H, CH_2_), 0.98 (t, *J* = 6.5 Hz, 3H, CH_3_). ^13^C NMR (101 MHz, MeOD) *δ* 158.27, 145.61, 135.67, 132.38, 130.79, 130.45, 130.07, 128.08, 125.59, 117.21, 112.67, 111.31, 110.27, 52.03, 49.23, 41.84, 30.37, 29.92, 23.24, 22.97, 14.17. MS (ESI^+^) m/z calcd for C_23_H_30_N_3_O_2_^+^ [M+H]^+^ 380.2, found 380.2. Purity = 95.6%.

***3d***: 3-(2-(benzylamino)ethyl)-1H-indol-5-yl hexylcarbamate: white solid (93% yield). ^1^H NMR (300 MHz, MeOD) *δ* 7.35 – 7.19 (m, 7H, aromatic protons), 7.06 (s, 1H, NH), 6.84 (dd, *J* = 8.8, 2.2 Hz, 1H, CH), 3.77 (s, 2H, CH_2_), 3.18 (t, *J* = 7.0 Hz, 2H, CH_2_), 2.91 (q, *J* = 4.4 Hz, 4H, CH_2_), 1.63 – 1.49 (m, 2H, CH_2_), 1.37 (m, 6H, CH_2_), 0.92 (t, *J* = 6.5 Hz, 3H, CH_3_). ^13^C NMR (75 MHz, CD_3_OD) *δ* 157.30, 144.36, 138.41, 134.62, 128.49, 128.38, 127.57, 127.27, 123.84, 115.87, 112.21, 111.37, 110.52, 52.84, 48.89, 40.89, 31.55, 29.70, 26.44, 24.52, 22.52, 13.27. MS (ESI^+^) m/z calcd for C_24_H_32_N_3_O_2_^+^ [M+H]^+^ 394.2, found 394.2. Purity = 98.8%.

***3e***: 3-(2-(benzylamino)ethyl)-1H-indol-5-yl heptylcarbamate: white solid (95% yield). ^1^H NMR (400 MHz, MeOD) *δ* 7.47 (m, *J* = 6.9, 2.8 Hz, 6H, aromatic protons), 7.35 (d, *J* = 8.7 Hz, 1H, NH), 7.27 (d, *J* = 2.2 Hz, 1H, aromatic proton), 7.22 (s, 1H, aromatic proton), 6.87 (dd, *J* = 8.7, 2.3 Hz, 1H, CH), 4.19 (d, *J* = 3.4 Hz, 2H, CH_2_), 3.28 (d, *J* = 7.8 Hz, 2H, CH_2_), 3.19 (t, *J* = 7.1 Hz, 2H, CH_2_), 3.13 (t, *J* = 8.0 Hz, 2H, CH_2_), 1.57 (t, *J* = 7.1 Hz, 2H, CH_2_), 1.46 – 1.28 (m, 8H, CH_2_), 0.96 – 0.88 (m, 3H, CH_3_). ^13^C NMR (101 MHz, MeOD) *δ* 158.27, 145.61, 135.68, 132.34, 130.80, 130.47, 130.08, 128.07, 125.59, 117.21, 112.67, 111.31, 110.24, 52.03, 49.23, 41.87, 32.77, 30.69, 29.94, 27.69, 23.48, 22.97, 14.22. HRMS (ESI^+^) m/z calcd for C_25_H_34_N_3_O_2_^+^ [M+H]^+^ 408.2646, found 408.2642. Purity = 98.0%.

***3f***: 3-(2-(benzylamino)ethyl)-1H-indol-5-yl cyclopropylcarbamate: white solid (88% yield). ^1^H NMR (400 MHz, MeOD) *δ* 7.38 (d, *J* = 3.3 Hz, 6H, aromatic protons), 7.32 (d, *J* = 8.7 Hz, 1H, NH), 7.25 (d, *J* = 2.2 Hz, 1H, aromatic proton), 7.13 (s, 1H, aromatic proton), 6.85 (dd, *J* = 8.7, 2.2 Hz, 1H, CH), 3.98 (s, 2H, CH_2_), 3.09 (m, 2H, CH_2_), 3.01 (m, 2H, CH_2_), 2.62 (t, *J* = 3.7 Hz, 1H, CH), 0.71 (m, 2H, CH_2_), 0.59 (q, *J* = 3.7 Hz, 2H, CH_2_). ^13^C NMR (101 MHz, MeOD) *δ* 159.08, 145.37, 135.62, 134.85, 130.29, 129.77, 129.60, 128.22, 125.34, 117.01, 112.58, 111.38, 111.33, 52.60, 49.44, 23.82, 6.50. MS (ESI^+^) m/z calcd for C_21_H_24_N_3_O_2_^+^ [M+H]^+^ 350.2, found 350.2. Purity = 97.0%.

***3g***: 3-(2-(benzylamino)ethyl)-1H-indol-5-yl cyclopentylcarbamate: white solid (89% yield). ^1^H NMR (400 MHz, MeOD) *δ* 7.50 – 7.42 (m, 5H, aromatic protons), 7.34 (d, *J* = 8.7 Hz, 1H, NH), 7.27 (d, *J* = 2.2 Hz, 1H, aromatic proton), 7.21 (s, 1H, aromatic proton), 6.87 (dd, *J* = 8.7, 2.2 Hz, 1H, CH), 4.18 (s, 2H, CH_2_), 4.04 – 3.91 (m, 1H, CH), 3.30 – 3.25 (m, 2H, CH_2_), 3.17 – 3.08 (m, 2H, CH_2_), 1.95 (m, 2H, CH_2_), 1.76 (m, 2H, CH_2_), 1.59 (m, 4H, CH_2_). ^13^C NMR (101 MHz, MeOD) *δ* 156.58, 144.40, 134.48, 131.18, 129.61, 129.27, 128.89, 126.88, 124.38, 116.05, 111.47, 110.15, 109.07, 52.76, 50.84, 32.28, 23.24, 21.79. HRMS (ESI^+^) m/z calcd for C_23_H_28_N_3_O_2_^+^ [M + H]^+^ 378.2176, found 378.2189. Purity = 97.6%.

***3h***: 3-(2-(benzylamino)ethyl)-1H-indol-5-yl cyclohexylcarbamate: white solid (86% yield). ^1^H NMR (400 MHz, MeOD) *δ* 7.50 – 7.40 (m, 5H, aromatic protons), 7.34 (d, *J* = 8.7 Hz, 1H, NH), 7.27 (d, *J* = 2.2 Hz, 1H, aromatic proton), 7.19 (s, 1H, aromatic proton), 6.87 (dd, *J* = 8.7, 2.3 Hz, 1H, CH), 4.14 (s, 2H, CH_2_), 3.45 (m, 1H, CH), 3.24 (m, 2H, CH_2_), 3.15 – 3.05 (m, 2H, CH_2_), 2.01 – 1.89 (m, 2H, CH_2_), 1.78 (m, 2H, CH_2_), 1.70 – 1.58 (m, 1H, CH_2_), 1.42 – 1.16 (m, 5H, CH_2_). ^13^C NMR (101 MHz, MeOD) *δ* 157.44, 145.56, 135.62, 132.50, 130.77, 130.36, 130.02, 128.08, 125.56, 117.22, 112.65, 111.36, 110.33, 52.03, 51.43, 33.87, 26.40, 25.98, 22.99. MS (ESI^+^) m/z calcd for C_24_H_30_N_3_O_2_^+^ [M+H]^+^ 392.2, found 392.2. Purity = 96.0%.

***3i***: 3-(2-(benzylamino)ethyl)-1H-indol-5-yl phenylcarbamate: white solid (93% yield). ^1^H NMR (400 MHz, MeOD) *δ* 7.55 – 7.49 (m, 2H, aromatic protons), 7.47 – 7.36 (m, 6H, aromatic protons), 7.35 (d, *J* = 2.2 Hz, 1H, NH), 7.33 – 7.27 (m, 2H, aromatic protons), 7.21 (s, 1H, aromatic proton), 7.09 – 7.03 (m, 1H, aromatic proton), 6.96 (dd, *J* = 8.7, 2.2 Hz, 1H, CH), 4.11 (d, *J* = 3.5 Hz, 2H, CH_2_), 3.22 (t, *J* = 7.2 Hz, 2H, CH_2_), 3.15 – 3.05 (m, 2H, CH_2_). ^13^C NMR (101 MHz, MeOD) *δ* 155.30, 145.15, 139.77, 135.80, 133.45, 130.58, 130.08, 129.94, 129.71, 128.19, 125.59, 124.21, 119.78, 117.08, 112.73, 111.46, 110.82, 52.26, 40.22, 23.36. MS (ESI^+^) m/z calcd for C_24_H_24_N_3_O_2_^+^ [M+H]^+^ 386.2, found 386.2. Purity = 96.3%.

***3j***: 3-(2-(benzylamino)ethyl)-1H-indol-5-yl dimethylcarbamate: white solid (92% yield). ^1^H NMR (400 MHz, CDCl_3_) *δ* 8.35 (s, 1H, NH), 7.30 (d, *J* = 4.4 Hz, 4H, aromatic protons), 7.25 – 7.23 (m, 2H, aromatic protons), 7.22 (s, 1H, aromatic proton), 6.92 – 6.86 (m, 2H, aromatic protons), 3.82 (s, 2H, CH_2_), 3.20 (s, 1H, CH), 3.14 (s, 3H, CH_3_), 3.03 (s, 3H, CH_3_), 2.91 (s, 4H, CH_2_). ^13^C NMR (101 MHz, MeOD) *δ* 155.75, 145.91, 136.03, 133.12, 130.66, 130.19, 129.98, 128.17, 125.97, 116.10, 112.91, 110.92, 110.79, 54.59, 52.24, 30.52, 23.24. MS (ESI^+^) m/z calcd for C_20_H_24_N_3_O_2_^+^ [M+H]^+^ 338.2, found 338.2. Purity = 95.3%.

***3k***: 3-(2-(benzylamino)ethyl)-1H-indol-5-yl diethylcarbamate: white solid (90% yield). ^1^H NMR (400 MHz, CDCl_3_) *δ* 8.50 (s, 1H, NH), 7.31 – 7.19 (m, 6H, aromatic protons), 7.15 (d, *J* = 8.7 Hz, 1H, aromatic proton), 6.87 (dd, *J* = 8.7, 2.3 Hz, 1H, aromatic proton), 6.79 (d, *J* = 1.8 Hz, 1H, CH), 3.78 (s, 2H, CH_2_), 3.44 (m 4H, CH_2_), 2.88 (s, 4H, CH_2_), 1.26 (m, 6H, CH_3_). ^13^C NMR (101 MHz, CDCl_3_) *δ* 155.40, 144.63, 139.58, 133.99, 128.35, 128.24, 127.56, 126.96, 123.37, 116.38, 113.35, 111.42, 110.94, 53.52, 49.01, 43.15, 42.17, 25.32, 14.22, 13.44. MS (ESI^+^) m/z calcd for C_22_H_28_N_3_O_2_^+^ [M+H]^+^ 366.2, found 366.2. Purity = 98.2%.

***3l***: 3-(2-(benzylamino)ethyl)-1H-indol-5-yl dipropylcarbamate: white solid (87% yield). ^1^H NMR (400 MHz, CDCl_3_) *δ* 8.42 (s, 1H, NH), 7.27 (d, *J* = 5.7 Hz, 4H, aromatic protons), 7.22 (m, *J* = 6.1, 1.4 Hz, 2H, aromatic protons), 7.14 (d, *J* = 8.7 Hz, 1H, aromatic proton), 6.85 (dd, *J* = 8.7, 2.3 Hz, 1H, aromatic proton), 6.79 (d, *J* = 1.9 Hz, 1H, CH), 3.77 (s, 2H, CH_2_), 3.36 (t, *J* = 7.6 Hz, 2H, CH_2_), 3.29 (t, *J* = 7.6 Hz, 2H, CH_2_), 2.87 (s, 4H, CH_2_), 1.67 (m, 4H, CH_2_), 0.94 (m, 6H, CH_3_). ^13^C NMR (101 MHz, CDCl_3_) *δ* 155.85, 144.71, 139.63, 133.97, 128.36, 128.23, 127.57, 126.96, 123.32, 116.40, 113.44, 111.39, 110.93, 53.55, 49.48, 49.16, 49.04, 25.35, 22.02, 21.27, 11.29. MS (ESI^+^) m/z calcd for C_24_H_32_N_3_O_2_^+^ [M+H]^+^ 394.2, found 394.2. Purity = 97.7%.

***3m***: 3-(2-(benzylamino)ethyl)-1H-indol-5-yl dibutylcarbamate: white solid (91% yield). ^1^H NMR (300 MHz, CDCl_3_) *δ* 9.44 (s, 1H, NH), 7.52 – 7.39 (m, 2H, aromatic protons), 7.37 – 7.18 (m, 5H, aromatic protons), 7.08 (d, *J* = 2.2 Hz, 1H, aromatic proton), 6.76 (dd, *J* = 8.7, 2.2 Hz, 1H, aromatic proton), 6.49 (s, 1H, CH), 3.89 (s, 2H, CH_2_), 3.39 (t, *J* = 7.5 Hz, 2H, CH_2_), 3.28 (t, *J* = 7.5 Hz, 2H, CH_2_), 2.95 – 2.80 (m, 2H, CH_2_), 2.71 (t, *J* = 7.8 Hz, 2H, CH_2_), 1.74 – 1.52 (m, 4H, CH_2_), 1.36 (m, 4H, CH_2_), 0.96 (m, 6H, CH_3_). ^13^C NMR (75 MHz, CDCl_3_) *δ* 156.05, 144.35, 133.86, 132.75, 129.68, 128.75, 128.61, 126.98, 124.26, 115.98, 111.92, 110.51, 110.11, 51.27, 47.46, 47.17, 46.92, 30.84, 30.12, 22.40, 20.01, 13.85. MS (ESI^+^) m/z calcd for C_26_H_36_N_3_O_2_^+^ [M+H]^+^ 422.3, found 422.3. Purity = 96.7%.

***3n***: 3-(2-(benzylamino)ethyl)-1H-indol-5-yl diphenylcarbamate: white solid (93% yield). ^1^H NMR (400 MHz, CDCl_3_) *δ* 8.29 (s, 1H, NH), 7.33 – 7.25 (m, 8H, aromatic protons), 7.22 (d, *J* = 2.3 Hz, 1H, aromatic proton), 7.20 – 7.10 (m, 7H, aromatic protons), 7.06 (d, *J* = 8.7 Hz, 1H, aromatic proton), 6.83 (dd, *J* = 8.7, 2.3 Hz, 1H, aromatic proton), 6.73 (d, *J* = 1.9 Hz, 1H, CH), 3.69 (s, 2H, CH_2_), 2.81 (s, 4H, CH_2_), 2.41 – 2.32 (m, 1H, NH). ^13^C NMR (101 MHz, CDCl_3_) *δ* 154.22, 144.33, 142.46, 139.44, 134.07, 128.98, 128.37, 128.23, 127.48, 127.01, 126.30, 123.49, 115.99, 113.49, 111.43, 110.80, 53.50, 48.96, 25.31. MS (ESI^+^) m/z calcd for C_30_H_28_N_3_O_2_^+^ [M+H]^+^ 462.2, found 462.2. Purity = 95.6%.

***3o***: 3-(2-(benzylamino)ethyl)-1H-indol-5-yl methoxy(methyl)carbamate: brown solid (85% yield). ^1^H NMR (300 MHz, CDCl_3_) *δ* 9.09 (s, 1H, NH), 7.44 – 7.37 (m, 2H, aromatic protons), 7.32 – 7.21 (m, 4H, aromatic protons), 7.22 – 7.16 (m, 2H, aromatic protons), 6.84 (dd, *J* = 8.6, 2.2 Hz, 1H, aromatic proton), 6.69 (s, 1H, CH), 5.86 (s, 1H, NH), 3.88 (s, 2H, CH_2_), 3.80 (s, 3H, CH_3_), 3.28 (s, 3H, CH_3_), 2.94 (t, *J* = 7.5 Hz, 2H, CH_2_), 2.85 (t, *J* = 6.8 Hz, 2H, CH_2_). ^13^C NMR (75 MHz, CDCl_3_) *δ* 156.40, 143.97, 134.12, 133.74, 129.47, 128.74, 128.42, 127.11, 124.36, 115.87, 111.95, 110.94, 110.59, 61.72, 51.76, 47.32, 35.61, 23.04. MS (ESI^+^) m/z calcd for C_20_H_24_N_3_O_2_^+^ [M+H]^+^ 462.2, found 462.2. Purity = 95.1%.

***3p***: 3-(2-(benzylamino)ethyl)-1H-indol-5-yl methyl(phenyl)carbamate: white solid (90% yield). ^1^H NMR (400 MHz, MeOD) *δ* 7.42 (d, *J* = 4.2 Hz, 3H, aromatic protons), 7.40 – 7.35 (m, 5H, aromatic protons), 7.33 (d, *J* = 8.7 Hz, 1H, aromatic proton), 7.28 (m, 2H, aromatic protons), 7.14 (s, 1H, aromatic proton), 6.88 (d, *J* = 8.7 Hz, 1H, CH), 3.98 (s, 2H, CH_2_), 3.42 (s, 3H, CH_3_), 3.10 (m, 2H, CH_2_), 3.03 (t, *J* = 7.4 Hz, 2H, CH_2_). ^13^C NMR (101 MHz, MeOD) *δ* 156.79, 145.57, 144.26, 135.73, 134.52, 130.35, 129.98, 129.80, 129.71, 128.20, 127.62, 127.02, 125.47, 116.85, 112.66, 111.28, 111.24, 52.50, 48.91, 38.46, 23.71. MS (ESI^+^) m/z calcd for C_25_H_26_N_3_O_2_^+^ [M+H]^+^ 400.2, found 400.2. Purity = 96.9%.

***3q***: 3-(2-(benzylamino)ethyl)-1H-indol-5-yl azetidine-1-carboxylate: brown solid (85% yield). ^1^H NMR (300 MHz, CDCl_3_) *δ* 9.23 (d, *J* = 11.6 Hz, 1H, NH), 7.62 (d, *J* = 23.6 Hz, 3H, aromatic protons), 7.34 (q, *J* = 3.1 Hz, 2H, aromatic protons), 7.31 – 7.28 (m, 1H, aromatic proton), 7.19 – 7.10 (m, 2H, aromatic protons), 6.82 (m, 1H, aromatic proton), 6.60 (d, *J* = 3.1 Hz, 1H, CH), 4.15 (d, *J* = 32.9 Hz, 4H, CH_2_), 3.85 (d, *J* = 2.9 Hz, 2H, CH_2_), 2.95 – 2.75 (m, 4H, CH_2_), 2.31 (m, 2H, CH_2_). ^13^C NMR (75 MHz, CDCl_3_) *δ* 155.63, 144.04, 134.05, 129.40, 128.69, 128.32, 127.08, 124.13, 116.11, 111.78, 110.86, 110.60, 51.45, 50.10, 49.19, 47.11, 22.92, 15.72. HRMS (ESI^+^) m/z calcd for C_21_H_24_N_3_O_2_^+^ [M+H]^+^ 350.1863, found 350.1860. Purity = 97.6%.

***3r***: 3-(2-(dibenzylamino)ethyl)-1H-indol-5-yl pyrrolidine-1-carboxylate: brown solid (88% yield). ^1^H NMR (400 MHz, MeOD) *δ* 7.32 (d, *J* = 3.2 Hz, 4H, aromatic protons), 7.31 – 7.25 (m, 2H, aromatic protons), 7.23 (d, *J* = 2.2 Hz, 1H, aromatic proton), 7.12 (s, 1H, CH), 6.86 (dd, *J* = 8.7, 2.3 Hz, 1H, aromatic proton), 3.87 (s, 2H, CH_2_), 3.62 (t, *J* = 6.6 Hz, 2H, CH_2_), 3.45 (t, *J* = 6.6 Hz, 2H, CH_2_), 2.99 (s, 4H, CH_2_), 1.99 (m, 4H, CH_2_). ^13^C NMR (101 MHz, MeOD) *δ* 154.94, 144.25, 137.20, 134.52, 128.47, 128.27, 127.37, 127.28, 123.78, 115.70, 111.59, 111.18, 110.29, 52.37, 48.47, 46.13, 46.07, 25.38, 24.56, 23.94. HRMS (ESI^+^) m/z calcd for C_22_H_26_N_3_O_2_^+^ [M+H]^+^ 364.2020, found 364.2025. Purity = 96.7%.

***3s***: 3-(2-(benzylamino)ethyl)-1H-indol-5-yl piperidine-1-carboxylate: brown solid (87% yield). ^1^H NMR (300 MHz, CDCl_3_) *δ* 9.14 (s, 1H, NH), 7.41 (d, *J* = 7.2 Hz, 2H, aromatic protons), 7.29 (m, 3H, aromatic protons), 7.21 (dd, *J* = 8.8, 1.4 Hz, 1H, aromatic proton), 7.11 (d, *J* = 1.8 Hz, 1H, aromatic proton), 5.96 (s, 1H, CH), 3.87 (s, 2H, CH_2_), 3.62 (s, 2H, CH_2_), 3.51 – 3.39 (m, 2H, CH_2_), 2.85 (t, *J* = 7.4 Hz, 2H, CH_2_), 2.75 (d, *J* = 7.2 Hz, 2H, CH_2_), 1.64 (s, 6H, CH_2_). ^13^C NMR (75 MHz, CDCl_3_) *δ* 155.12, 144.46, 133.92, 129.46, 128.72, 128.34, 127.13, 124.07, 116.16, 111.85, 110.81, 110.69, 51.78, 47.42, 45.15, 25.53, 24.27, 22.94. MS (ESI^+^) m/z calcd for C_23_H_28_N_3_O_2_^+^ [M+H]^+^ 378.2, found 378.2. Purity = 95.2%.

***3t***: 3-(2-(benzylamino)ethyl)-1H-indol-5-yl azepane-1-carboxylate: white solid (93% yield). ^1^H NMR (300 MHz, CDCl_3_) δ 8.96 (s, 1H, NH), 7.41 – 7.34 (m, 2H, aromatic protons), 7.34 – 7.25 (m, 3H, aromatic protons), 7.21 (d, *J* = 8.7 Hz, 1H, aromatic proton), 7.15 (d, *J* = 2.2 Hz, 1H, aromatic proton), 6.82 (dd, *J* = 8.6, 2.2 Hz, 1H, aromatic proton), 6.65 (s, 1H, CH), 4.86 (s, 1H, NH), 3.84 (s, 2H, CH_2_), 3.65 – 3.56 (m, 2H, CH_2_), 3.49 (t, *J* = 6.0 Hz, 2H, CH_2_), 2.82 (m, 4H, CH_2_), 1.87 – 1.72 (m, 4H, CH_2_), 1.72 – 1.53 (m, 4H, CH_2_). ^13^C NMR (75 MHz, CDCl_3_) δ 155.97, 144.54, 135.58, 133.94, 129.12, 128.62, 127.97, 127.26, 123.89, 116.26, 111.72, 111.53, 110.77, 47.85, 47.35, 47.10, 28.65, 28.10, 27.46, 26.94, 23.59. MS (ESI^+^) m/z calcd for C_24_H_30_N_3_O_2_^+^ [M+H]^+^ 392.2, found 392.2. Purity = 97.4%.

***3u*:** 3-(2-(benzylamino)ethyl)-1H-indol-5-yl morpholine-4-carboxylate: white solid (92% yield). ^1^H NMR (400 MHz, CDCl_3_) *δ* 8.97 (s, 1H, NH), 7.44 – 7.36 (m, 2H, aromatic protons), 7.29 (t, *J* = 7.0 Hz, 2H, aromatic protons), 7.26 – 7.19 (m, 2H, aromatic protons), 7.17 (d, *J* = 2.3 Hz, 1H, aromatic proton), 6.82 (dd, *J* = 8.7, 2.2 Hz, 1H, aromatic proton), 6.66 (s, 1H, CH), 5.96 (s, 1H, NH), 3.86 (s, 2H, CH_2_), 3.79 – 3.71 (m, 4H, CH_2_), 3.68 (s, 2H, CH_2_), 3.52 (s, 2H, CH_2_), 2.91 (t, *J* = 6.9 Hz, 2H, CH_2_), 2.83 (t, *J* = 6.9 Hz, 2H, CH_2_). ^13^C NMR (101 MHz, CDCl_3_) *δ* 155.00, 144.30, 134.62, 134.04, 129.30, 128.70, 128.23, 127.22, 124.09, 116.09, 111.82, 111.31, 110.72, 66.55, 52.02, 47.63, 44.80, 44.11, 23.32. MS (ESI^+^) m/z calcd for C_22_H_26_N_3_O_3_^+^ [M+H]^+^ 380.2, found 380.2. Purity = 97.4%.

***4c*:** 3-(2-aminoethyl)-1H-indol-5-yl pentylcarbamate: white solid (90% yield). ^1^H NMR (400 MHz, CDCl_3_) *δ* 8.82 (s, 1H, NH), 7.28 – 7.21 (m, 1H, aromatic proton), 7.12 (d, *J* = 8.7 Hz, 1H, aromatic proton), 6.88 – 6.78 (m, 2H, aromatic protons), 5.27 (t, *J* = 5.8 Hz, 1H, CH), 3.22 (q, *J* = 6.7 Hz, 2H, CH_2_), 2.89 (t, *J* = 6.6 Hz, 2H, CH_2_), 2.75 (t, *J* = 6.6 Hz, 2H, CH_2_), 1.63 – 1.46 (m, 4H, CH_2_), 1.36 – 1.24 (m, 4H, CH_2_), 0.92 – 0.82 (m, 3H, CH_3_). ^13^C NMR (101 MHz, CDCl_3_) *δ* 155.95, 144.25, 134.21, 127.68, 123.65, 116.21, 113.34, 111.56, 110.96, 42.12, 41.31, 29.57, 29.18, 28.95, 22.36, 14.01. MS (ESI^+^) m/z calcd for C_16_H_24_N_3_O_2_^+^ [M+H]^+^ 290.2, found 290.2. Purity = 95.0%.

***4d***: 3-(2-aminoethyl)-1H-indol-5-yl hexylcarbamate: white solid (93% yield). ^1^H NMR (400 MHz, CDCl_3_) *δ* 8.80 (s, 1H, NH), 7.27 – 7.20 (m, 1H, aromatic proton), 7.12 (d, *J* = 8.7 Hz, 1H, aromatic proton), 6.88 – 6.77 (m, 2H, aromatic protons), 5.25 (t, *J* = 5.9 Hz, 1H, CH), 3.21 (q, *J* = 6.7 Hz, 2H, CH_2_), 2.89 (t, *J* = 6.6 Hz, 2H, CH_2_), 2.75 (t, *J* = 6.6 Hz, 2H, CH_2_), 1.53 (m, 8.4 Hz, 4H, CH_2_), 1.34 – 1.19 (m, 6H, CH_2_), 0.86 (t, *J* = 6.7 Hz, 3H, CH_3_). ^13^C NMR (101 MHz, CDCl_3_) *δ* 155.94, 144.26, 134.21, 127.69, 123.64, 116.22, 113.37, 111.56, 110.96, 42.12, 41.34, 31.48, 29.85, 29.20, 26.47, 22.58, 14.03. MS (ESI^+^) m/z calcd for C_17_H_26_N_3_O_2_^+^ [M+H]^+^ 304.2, found 304.2. Purity = 95.8%.

***4e***: 3-(2-aminoethyl)-1H-indol-5-yl heptylcarbamate: white solid (93% yield). ^1^H NMR (400 MHz, MeOD) *δ* 7.33 (d, *J* = 8.7 Hz, 1H, aromatic proton), 7.27 (d, *J* = 2.3 Hz, 1H, aromatic proton), 7.16 (s, 1H, CH), 6.85 (dd, *J* = 8.7, 2.2 Hz, 1H, aromatic proton), 3.18 (t, *J* = 7.1 Hz, 2H, CH_2_), 3.06 (t, *J* = 6.9 Hz, 2H, CH_2_), 2.97 (t, *J* = 7.0 Hz, 2H, CH_2_), 1.56 (q, *J* = 7.0 Hz, 2H, CH_2_), 1.39 – 1.30 (m, 8H, CH_2_), 0.95 – 0.89 (m, 3H, CH_3_). ^13^C NMR (101 MHz, MeOD) δ 157.13, 144.28, 134.52, 127.19, 124.14, 115.78, 111.30, 110.53, 110.17, 40.66, 40.62, 31.58, 29.48, 28.74, 26.48, 25.18, 22.28, 13.03. MS (ESI^+^) m/z calcd for C_18_H_28_N_3_O_2_^+^ [M+H]^+^ 318.2, found 318.2. Purity = 95.5%.

### Inhibition assay on AChE and BuChE

The ChE inhibition activity of the synthesized CTDs was performed according to modified Ellman’s method. Donepezil and rivastigmine were used as reference standards. Butyrylcholinesterase (BuChE, E.C. 3.1.1.8, from equine serum), acetylcholinesterase (AChE, E.C. 3.1.1.7, Type V-S, lyophilized powder, from electric eel, 1000 unit), butyrylthiocholine iodide (BTCI), acetylthiocholine iodide (ATCI), and 5,5-dithiobis-(2-nitrobenzoic acid) (DTNB) were purchased from Sigma-Aldrich. The tested compounds were dissolved in DMSO (1%, analytical grade) and then diluted in phosphate buffer (75 mM, pH 7.4) to obtain the terminal concentrations required. At least ten concentration gradients were set for each compound in triplicate to detect the inhibition rate for BuChE, and five concentration gradients were set for each compound in triplicate to detect the inhibition rate for AChE. Specific experiment processes were conducted as in previous works (Zhang et al., 2022a). Specifically, 10 µl prepared AChE or BuChE solution (1.0 U/ml), 25 µl prepared tested compounds solution, and 65 µl phosphate buffer (pH 8.0, 0.1 mol/L) were mixed in each well of 96-well plates. Then, the 96-well plates were preincubated for 20 min at 37°C in a constant temperature incubator. The wells in the control group were operated under the same conditions without inhibitors, and the wells in the blank group were operated under the same conditions without enzyme solutions and inhibitors. Subsequently, 100 µl prepared DTNB solution (0.35 mM) and 50 µl prepared BTCI or ATCI solution (1.0 mM) were quickly added to each well, and the mixture reacted 5 min at room temperature. The OD values were tested at 412 nm using a microplate reader (Bio-Rad Laboratories, CA, United States). The IC_50_ values were calculated using IBM SPSS Statistics 25.0 software. All experiments were performed in triplicate, and the results were shown as the mean ± SD.

### Kinetic study

In the kinetic study, the preincubated time of the mixture (BuChE, inhibitors solution, and phosphate buffer) were 2, 5, 10, 15, 20, 30, 40, 50, and 60 min, respectively. Other than that, the rest operation was the same as mentioned above. The obtained activities of BuChE were plotted against time and fitted to equation (A = A_0_ • e^-kobst^ + A_∞_) to determine rate constant k_obs_ by GraphPad Prism 8.0 software. Thereinto, A stands for the activity of BuChE at each time t, A_0_ stands for the activity of BuChE at time *t* = 0, and A_∞_ stands for the activity of BuChE at infinite time. Then, the created reciprocal k_obs_^−1^ values needed to be plotted against the reciprocal concentration C^−1^. According to the plot, k_3_ is calculated from the *Y*-intercept, and k_c_ was calculated from the slope of the resulting linearization according to equation (k_obs_^−1^ = k_c_ • k_3_^−1^ • C^−1^ + k_3_^−1^) by GraphPad Prism 8.0 software.

### Molecular docking study

Schrodinger software (Release 2019-2, Schrodinger, LLC, New York, NY, 2019) is used to conduct a molecular docking study. The structures of the *h*BuChE (PDB code: 4TPK) and *h*AChE (PDB code: 4M0F) are retrieved from the RCSB Protein Data Bank (http://www.rcsb.org/pdb/). According to the previous method (Liu et al., 2022), the crystal structure profiles of BuChE and AChE are prepared by the Maestro Protein Preparation Wizard model by adding hydrogen atoms and straining minimization using the OPLS3 force field. And the ionization state is set at pH 7.0 ± 2.0. Then, the structures of the tested compounds are added to the hydrogen atoms at a neutralized environment, minimized by MMFFs forcefield, generating 3D coordinates. The binding sites of N-((1-(2,3-dihydro-1H-inden-2-yl) piperidin-3-yl) methyl)-N-(2-methoxyethyl)-2-naphthamide and territrem B in BuChE and AChE, respectively, are selected as the active sites for docking. The receptor grid is created at the selected residues using the grid box at the size of 20Å. Ultimately, molecular docking is run using the standard precision (SP). Other than above mentioned, the other docking parameters are set as default.

### Evaluation of cell viability

The cell lines present in this study were purchased from the China Center for Type Collection (CCTCC, China). For cytotoxicity assay, HT-22, BV2, SH-SY5Y, HepG2, and LO2 cells were incubated in 96-well plates at the density of 5 × 10³ per well. After incubation for 12 h at 37°C, the tested compounds with the required concentrations were added to the corresponding wells and continuously incubated for 24 h at 37°C. Then, the prepared MTT (3-[4,5-dimethyl-2-thiazolyl]-2,5-diphenyl-2H-tetrazolium bromide) solution was added to each well and co-incubated for another 4 h at 37°C. Then, the supernatant was removed, and 100 μl DMSO was added to each well. The OD value of each well was detected at 570 nm using a microplate reader (Bio-Rad Laboratories, CA, United States). The IC_50_ values were calculated using IBM SPSS Statistics 25.0 software. All experiments were performed in triplicate, and the results were shown as the mean ± SD.

For the neuroprotection assay, HT-22 cells were incubated in 96-well plates at the density of 5 × 10³ per well. After incubation for 12 h at 37°C, the tested compounds with required concentrations and H_2_O_2_ (500 μM) were added to the corresponding wells and continuously incubated for 24 h at 37°C. Then, the prepared MTT (3-[4,5-dimethyl-2-thiazolyl]-2,5-diphenyl-2H-tetrazolium bromide) solution was added to each well and co-incubated for another 4 h at 37°C. Then, the supernatant was removed, and 100 μl DMSO was added to each well. The OD value of each well was detected at 570 nm using a microplate reader (Bio-Rad Laboratories, CA, United States). The cell viability values were calculated following the following equation: (OD_570_ (tested compounds) –OD_570_ (blank))/(OD_570_ (control) – OD_570_ (blank)) × 100%. All experiments were performed in triplicate, and the results were depicted as the mean ± SD.

### ORAC assay

Trolox was considered as standard, and its ORAC value was set as 1. Tested compounds in required concentrations (20 μl) and fluorescein solution (120 μl) were first co-incubated in a 96-well plate for 15 min at 37°C. Next, AAPH solution (60 µl) was added to each well. Then, the plate was immediately placed into a microplate reader, and the fluorescence of each well was detected every 2 min for 90 min with excitation at 485 nm and emission at 535 nm. After that, the fluorescence intensity was plotted on the vertical axis, and time was plotted on the horizontal axis. The antioxidant curves were normalized to the curve of the blank in the same assay. The AUC (area under the fluorescence decay curve) was obtained using GraphPad Prism 8.0 software. Net AUC = AUC_antioxidant_ – AUC_blank_. Subsequently, the net AUC value was plotted on the vertical axis, and antioxidant concentration was plotted on the horizontal axis. ORAC value = slope_antioxidant_/slope_Trolox._ All experiments were conducted in triplicate, and the terminal ORAC values were depicted as the mean ± SD.

### Inhibition assay on COX-2

Inhibition assay on COX-2 was performed using a commercially available COX-2 screening assay kit (Beyotime, China, lot: S0168). At the initial screening, the tested concentrations of the selected compounds were 40, 20, 10, 5, and 2.5 μM. The reference concentrations (celecoxib) were 1.28, 0.64, 0.32, 0.16, and 0.08 μM. The specific experimental operations were conducted following the instruction of the assay kit. All experiments were conducted in triplicate, and the terminal inhibitory results were depicted as the mean ± SD.

### Statistical analysis

All results were determined by ANOVA (analysis of variance) following Fisher’s PLSD procedure for *post hoc* comparison to verify the significance between the two means. *p* values less than 0.05 were considered statistically significant. The data were handled by SPSS 25.0 software.

## Results and Discussion

### Synthesis of target compounds

**SCHEME 2:**
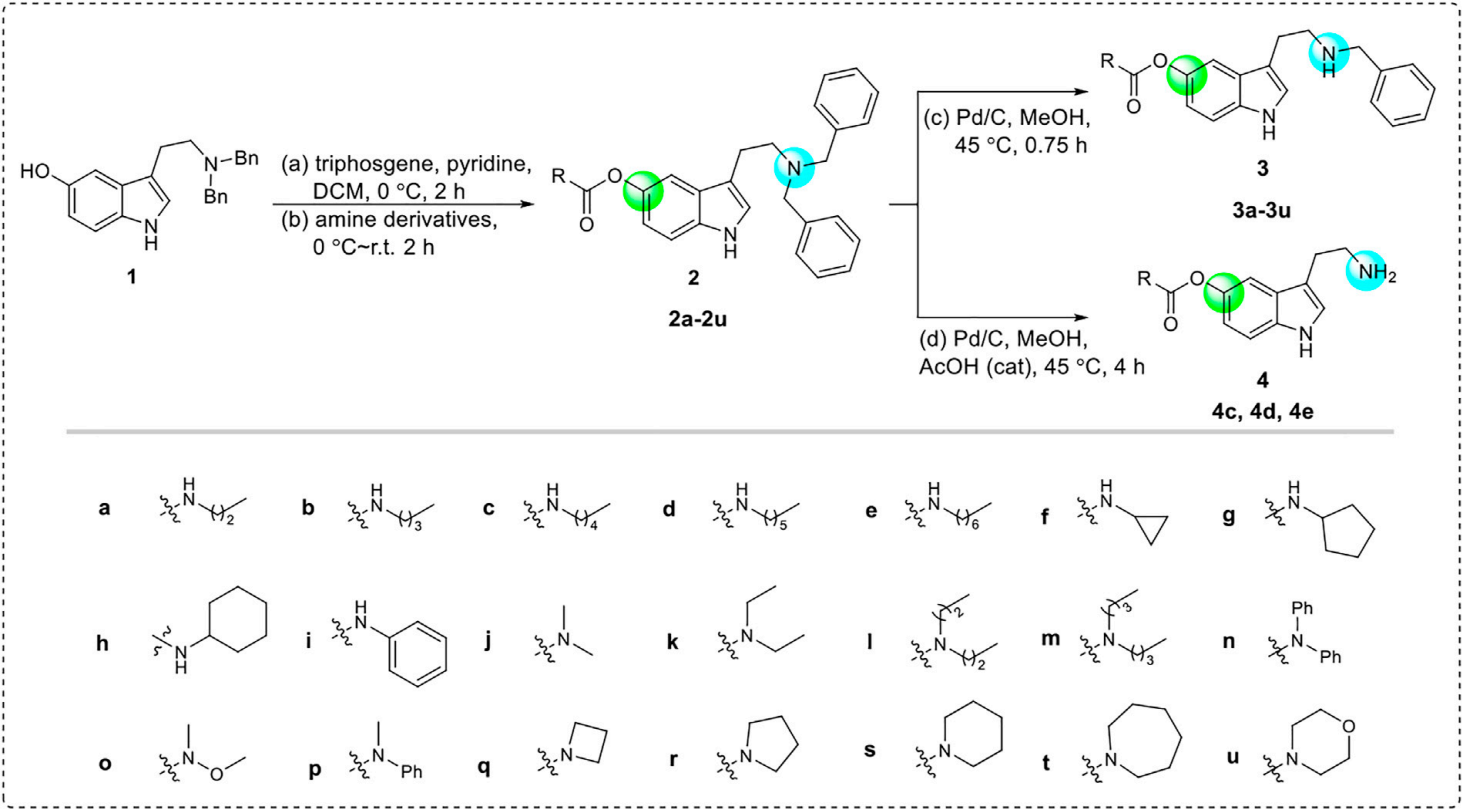
Synthetic method for the targeted carbamylated tryptamine derivatives.

The starting material **1** was synthesized from commercially available 5-methoxytryptamine *via* the process of amino protection and demethylation (Zhang et al., 2022a). Subsequently, the benzyl-protected tryptamine analog **1** was acylated under the condition of triphosgene and then reacted with diverse amines to give the CTDs **2a**-**2u**. Then, selectively removing one or two benzyl groups by controlling the reaction conditions, the CTDs **3a-3u** or **4c-4e** were obtained, respectively (Scheme 2). The structures of these CTDs were characterized by HRMS and nuclear magnetic resonance (NMR). All of them were produced in mild-to-good yields (53%–95%). The purity of these products was detected by high-performance liquid chromatography (HPLC) analysis and reached more than 95%.

### Inhibition assay on AChE and BuChE

**FIGURE 2:**
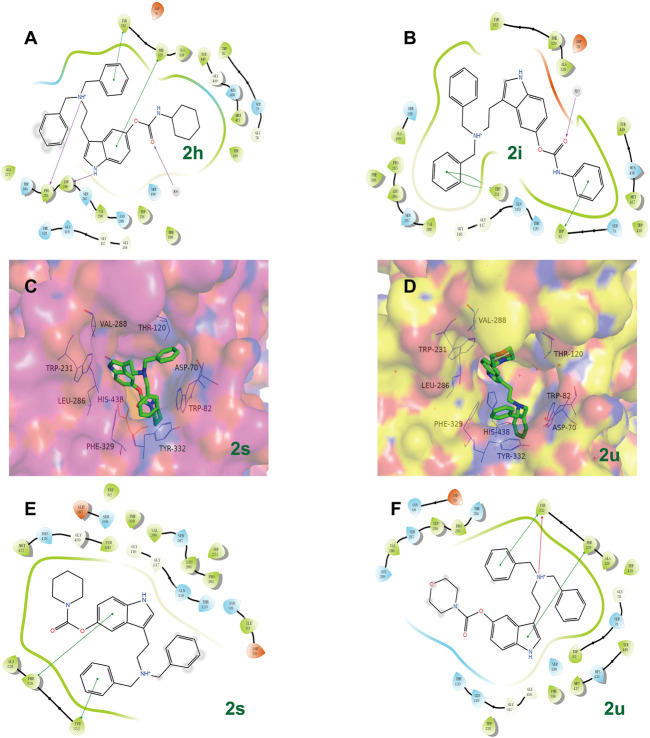
The possible binding mode for the compounds **2h**, **2i**, **2s**, **2u** in the BuChE (PDB code: 4TPK) binding sites. **(A,B,E,F)** The 2D images of **2h**, **2i**, **2s**, **2u**, respectively, binding to BuChE predicted by Schrodinger software (Release 2019-2, Schrodinger, LLC, New York, NY, 2019). The purple arrow indicates hydrogen bond, the red arrow indicates π–cation interaction, and the green line indicates π–π interaction. **(C,D)** The potential distribution surface diagrams of **2s** and **2u**, respectively, created by Pymol (http://www.pymol.org).

**FIGURE 3:**
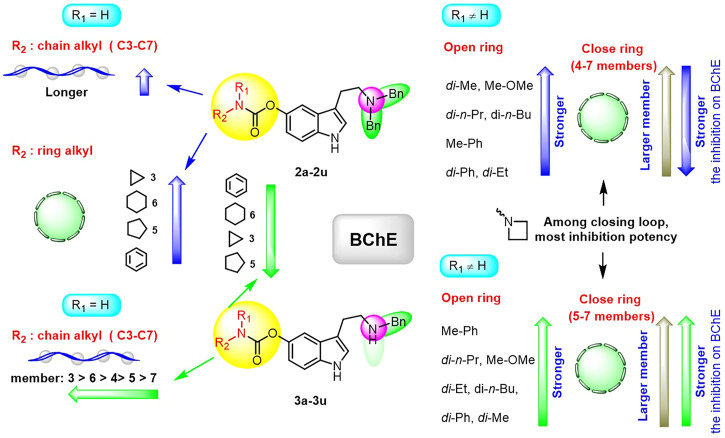
Outline of SARs of compounds **2a-2u** and **3a-3u** on BuChE.

Ellman’s colorimetric assay was performed to evaluate the inhibitory activity of synthesized compounds on acetylcholinesterase (AChE) and BuChE, and donepezil and rivastigmine were selected as the reference ChE inhibitors (Hoffmann et al., 2019). The dibenzyl-substituted carbamylated compounds (**2a-2u**) were first selected to study the structure-activity relationships (SARs) of different carbamate moieties for ChE inhibition (Table 1; Figure 3). The results showed that most of these compounds had a poor inhibitory effect on AChE, except for N-methoxymethylamine (**2o**) and azetidine (**2p**) substituted compounds. However, all these compounds possessed good-to-excellent inhibition potency on BuChE. In detail, for the compounds bearing terminal secondary amine carbamate group were chain alkyl amine, compounds **2a-2e** bearing three to seven carbon chains showed great inhibitory activity. Among them, the inhibition efficacy of **2a** with three-carbon chain was slightly weaker than that of **2c-2e** with longer carbon chains. When the terminal residues were ring carbon chains, the activity of ternary ring (**2f**) was significantly stronger than that of the six-membered ring (**2h**) and five-membered ring (**2g**). When the alkyl ring was replaced with a benzene ring, the activity of **2i** was obviously decreased, indicating that the existence of π electrons for this scaffold probably had a negative influence on the inhibition potency of BuChE. In order to preliminarily verify this hypothesis, the molecular docking study was performed. The result showed that **2h** could form a hydrogen bond interaction with Pro285 and Leu286, which was the key residue in the acyl-binding pocket of BuChE (PDB: 4TPK). Meanwhile, it could also form π–π stacking interactions with Tyr332 and Phe329, which were the residues in the peripheral anion site (PAS) (Figures 2A,B). Differently, **2i** could only form π–π stacking interactions with Trp231 and Trp82, which were the key residues in the acyl-binding pocket and choline-binding pocket of BuChE, respectively. This observation indicated that the subtle difference might be the reason for the lower activity of **2i** than **2h**. Moreover, when the terminal residue was replaced with tertiary amine carbamate moieties, the different sizes and steric hindrance of substituents had different effects on the activity. For the ring-opening amino segment, dimethylamine (**2j**) and methoxymethylamine (**2o**) substituted compounds possessed great inhibition potency. Compounds with dipropylamine (**2l**), dibutylamine (**2m**), and N-methylaniline (**2p**) also exhibited favorable inhibition potency. However, the inhibition potency of compounds with diethylamine (**2k**) and diphenylamine (**2n**) was relatively weak. All these results showed that the inhibition potency on BuChE was not only regulated by the steric hindrance, but also adjusted by the size of the carbamate residue. When the carbamate residues were relatively rigid alkylamine fragments (**2q-2t**), the inhibition efficacy was decreased with the expanding ring. Among them, **2p** with azetidine residue exhibited quite inhibitory activity against BuChE. Besides, when the cyclohexane was replaced with morpholine (**2u**), the inhibition efficacy was obviously decreased. According to the phenotypic result of the molecular docking study, the introduction of morpholine changed the lowest energy conformation of the compound, and the subtle variation weakened the affinity of the indole ring to the acyl-binding pocket and the whole compound to the active sites of BuChE (Figures 2C–F). As for the subtle changes in the study of SAR, further in-depth research is ongoing to clarify the observation.

**TABLE 1:** AChE and BuChE inhibitory activity (IC_50_) of compounds **2a-2u**.

Comp.	R	IC_50_ ± SD		SI^b^ (BuChE)	Comp.	R	IC_50_ ± SD		SI^b^ (BuChE)
		BuChE^a^ (nM)	AChE^a^ (nM)				BuChE^a^ (nM)	AChE^a^ (nM)	
**Don.**		3702.48 + 28.43	2450.14 ± 80.29	0.66	**2k**	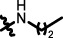	320.88 ± 2.06	>100 μM	>312
**Riv.**		231.84 ± 20.09	371.23 ± 10.46	1.60	**2l**	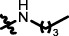	15.90 ± 0.55	>100 μM	>6,289
**2a**	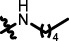	33.18 ± 4.99	>100 μM	>3,013	**2m**	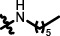	13.82 ± 0.67	>100 μM	>7,236
**2b**	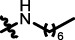	3.00 ± 0.21	>100 μM	>33,333	**2n**	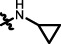	262.27 ± 2.48	>100 μM	>381
**2c**	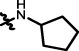	2.10 ± 0.14	>100 μM	>47,619	**2o**	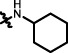	8.65 ± 0.34	2,931.26 ± 17.98	339
**2d**	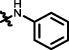	1.47 ± 0.15	>100 μM	>68,027	**2p**	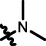	18.85 ± 1.34	>100 μM	>5,305
**2e**	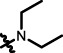	1.42 ± 0.004	>100 μM	>70,422	**2q**	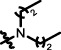	1.65 ± 0.03	742.16 ± 9.87	450
**2f**	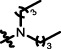	8.75 ± 0.23	>100 μM	>11,428	**2r**	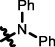	20.47 ± 1.70	>100 μM	>4,885
**2g**	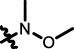	33.69 ± 1.62	>100 μM	>2,968	**2s**	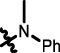	33.35 ± 5.21	>100 μM	>2,998
**2h**	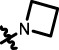	15.98 ± 0.32	>100 μM	>6,258	**2t**	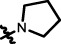	67.97 ± 8.42	>100 μM	>1,471
**2i**	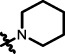	55.78 ± 3.14	>100 μM	>1,793	**2u**	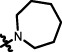	114.49 ± 1.45	>100 μM	>873
**2j**	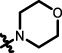	6.77 ± 0.27	>100 μM	>14,771					

^a^ AChE from electric eel and BuChE from equine serum.

^b^ SI (BuChE) = IC_50_(AChE)/IC_50_(BuChE). SI: selection index. Enzyme activity was examined by Ellman’s colorimetric assay. IC_50_ values were calculated as the mean ± SD of triplicate in three independent experiments. Don, donepezil. Riv, rivastigmine.

By considering the influence of the benzyl group on the affinity of compounds to the enzyme, further SAR studies on **3a-3u** with monobenzyl substituent and **4c-4e** without benzyl group were evaluated, and the results are depicted in Table 2 and Figure 3. Overall, the reduction or elimination of benzyl groups decreased the BuChE inhibitory activity of the compounds. Noteworthily, most monobenzyl-substituted CTDs possessed mild-to-good inhibition efficacy on AChE, significantly different from dibenzyl-substituted carbamylated tryptamine derivatives.

**TABLE 2:** AChE and BuChE inhibitory activity (IC_50_) of compounds **3a-3u**, **4c-4e**.

Comp.	R	IC_50_ ± SD^a^		SI^b^ (BuChE)	Comp.	R	IC_50_ ± SD^a^		SI^b^ (BuChE)
		BuChE^a^ (nM)	AChE^a^ (nM)				BuChE^a^ (nM)	AChE^a^ (nM)	
**Don.**		3,702.48 + 28.43	2,450.14 ± 80.29	0.66	**3l**	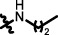	43.97 ± 1.84	>100 μM	>2,274
**Riv.**		231.84 ± 20.09	371.23 ± 10.46	1.60	**3m**	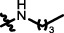	97.43 ± 8.53	19,782.51 ± 32.69	203
**3a**	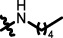	18.17 ± 0.68	1,952.31 ± 14.96	107	**3n**	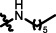	155.57 ± 3.58	>100 μM	>643
**3b**	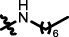	23.69 ± 1.18	1,692.45 ± 12.69	71	**3o**	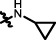	58.83 ± 2.92	2,613.24 ± 7.69	44
**3c**	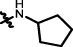	23.89 ± 2.90	1,133.26 ± 11.54	47	**3p**	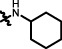	25.43 ± 0.27	7,053.68 ± 41.62	277
**3d**	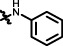	19.01 ± 0.29	2,443.63 ± 16.95	128	**3q**	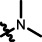	2.58 ± 0.13	482.16 ± 5.99	187
**3e**	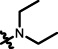	33.57 ± 1.64	1,132.45 ± 9.89	34	**3r**	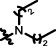	40.49 ± 1.79	1,815.47 ± 14.11	45
**3f**	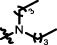	15.74 ± 1.30	3,174.86 ± 14.57	202	**3s**	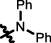	33.86 ± 2.14	9,076.38 ± 62.31	268
**3g**	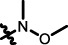	13.59 ± 0.70	27,183.67 ± 18.74	2,000	**3t**	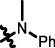	23.24 ± 0.94	>100 μM	>4,303
**3h**	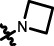	43.55 ± 3.35	>100 μM	>2,296	**3u**	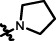	112.15 ± 7.37	>100 μM	>892
**3i**	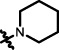	110.44 ± 5.22	23,291.64 ± 43.12	211	**4c**	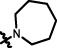	120.91 ± 8.38	>100 μM	>827
**3j**	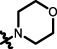	131.52 ± 3.59	1,913.07 ± 15.74	15	**4d**	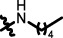	27.69 ± 2.04	>100 μM	>3,611
**3k**	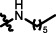	72.46 ± 6.55	>100 μM	>1,380	**4e**	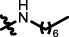	10.05 ± 0.83	>100 μM	>9,950

^a^ AChE from electric eel and BuChE from equine serum.

^b^ SI (BuChE) = IC_50_(AChE)/IC_50_(BuChE). SI: selection index. Enzyme activity was examined by Ellman’s colorimetric assay. IC_50_ values were calculated as the mean ± SD of triplicate in three independent experiments. Don, donepezil. Riv, rivastigmine.

For more details, the BuChE inhibitory activity of these compounds exhibited different SAR trends from the dibenzyl-substituted compounds, which might be due to the variation of the carrier scaffold. When the carbamate residue was the terminal secondary alkyl amine, the length of carbon chains or the size of ring carbon chains exhibited a slight influence on the inhibitory activity (**3a-3h**). Similar to the dibenzyl-substituted tryptamine derivatives, the replacement of cyclohexane with the benzene ring, the inhibitory activity of **3i** obviously decreased. When the terminal residue was the ring-opening tertiary amine carbamate moiety, the trend of the inhibitory activity of **3j-3p** against BuChE was significantly different from the results of **2j-2p**. Combining the SARs results of dibenzyl-substituted tryptamine derivatives, the variation further suggested that the inhibition potency was regulated by both the carbamate moiety and the size of the carrier scaffold. When the terminal residue was replaced with the relatively rigid alkylamine fragments, the compound with azetidine residue (**3q**) exhibited significant inhibition potency on BuChE, and the morpholine substituted compound **3u** exhibited weak inhibition potency, similar to the observed phenomenon in **2q** and **2u**. However, the inhibitory activity was increased with the expanding ring with five-to-seven members (**3r-3t**), inconsistent with the observed trend of disubstituted compounds.

**FIGURE 4:**
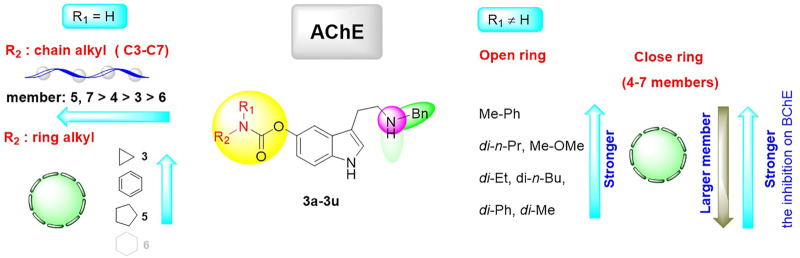
Outline of SARs of compounds **3a-3u** on AChE.

Besides, according to the IC_50_ values of the AChE inhibition assay, the length of the carbon chain of secondary amine in the carbamate group had a weak influence on AChE inhibition (**3a-3e**). When the unbranched alkyl group of **3a-3e** was replaced with cycloalkyl (**3f-3h**), the resulting compounds suffered a 1.3–88-fold decrease in the AChE inhibitory activity. When aniline replaced cyclohexylamine, the inhibitory activity on AChE of **3i** was markedly enhanced. The AChE inhibitory activity of different tertiary amine derivatives (**3j-3n** and **3p**) was also investigated. Among them, compound **3i** with a small bulky tertiary amine had the optimal potency on AChE. The methoxamine analog **3o** displayed comparable activity against AChE to that of **3i**. In addition, we explored the effect of different nitrogen-containing heterocycles (**3q-3u**) on the inhibitory activity against AChE. Interestingly, compound **3q** bearing an azetidinyl group showed comparable AChE inhibitory activity to that of rivastigmine, providing valuable guidance for further research. The summary of the SARs study is depicted in Figure 4.

In addition, the CTDs **4c-4e** without benzyl groups also possessed good inhibitory activity on BuChE (Table 2). The longer length of carbon chains had positive effects on the BuChE inhibition efficacy. However, the overall activity of the non-benzyl group was weaker than that of benzyl-substituted compounds. At the same time, considering the stability of this series of compounds, the study only evaluated the inhibition potency of **4c-4e,** and the results further confirmed the importance of the benzyl group for the affinity of tryptamine derivatives to the enzyme.

### Molecular docking

**FIGURE 5:**
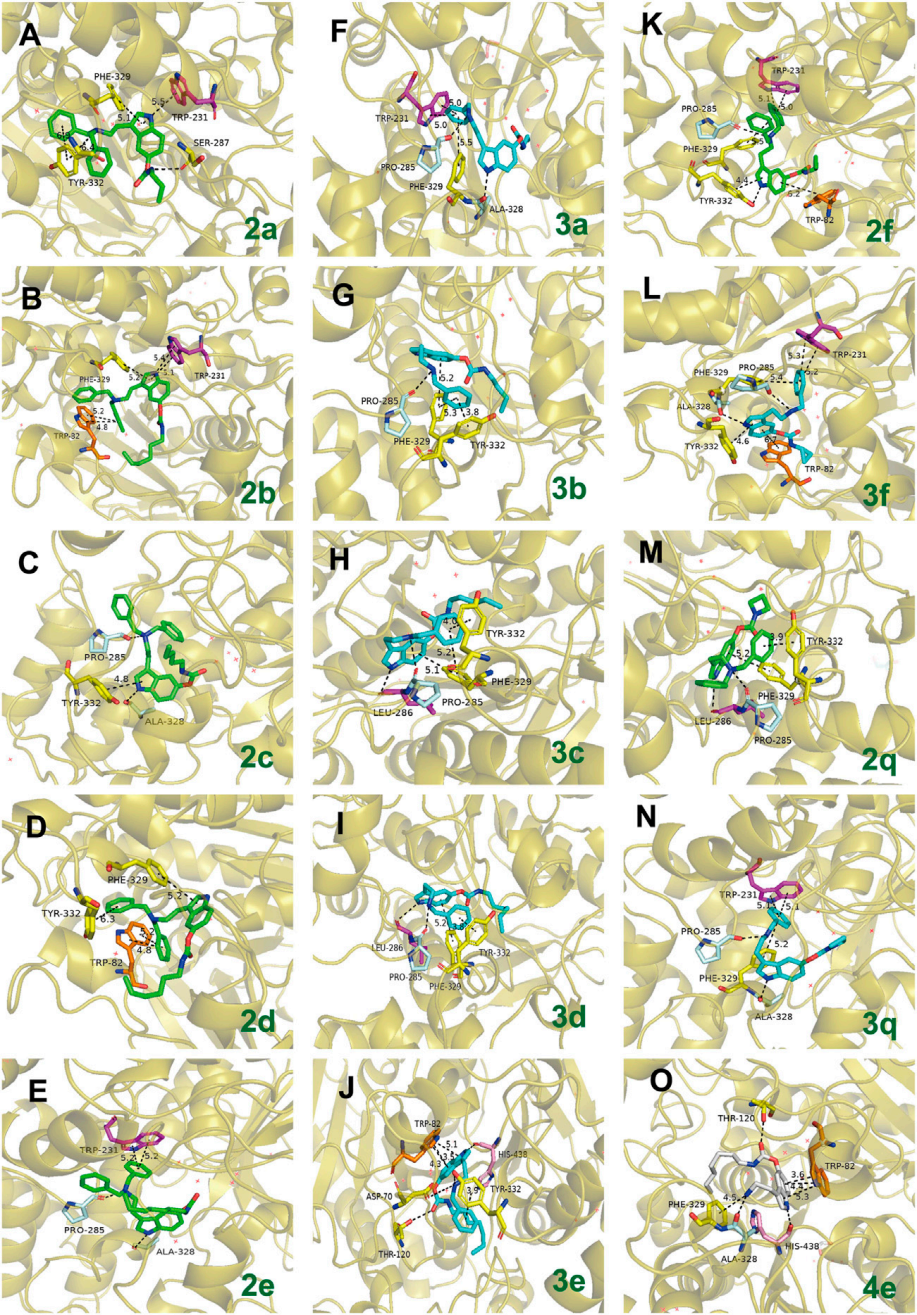
The possible binding mode for the selected compounds in the BuChE (PDB code: 4TPK) binding sites **(A–O)**. The 3D images of selected compounds bind to BuChE polished by Pymol (http://www.pymol.org). Residues of the acyl-binding pocket (Leu286, Trp231) are shown in magenta, the CAS (His438) is shown in pink, the choline-binding site (Trp82) is shown in orange, the residues of PAS (Asp70, Thr120, Tyr332, and Phe329) are shown in yellow, and parts of the side cavity (Pro285, Ala328) are in pale cyan. The dashed lines indicate hydrogen bond interaction, π–π stacking interactions, or π–cation interactions.

A molecular docking study was performed to predict the binding mode of the synthesized CTDs to *h*BuChE (PDB code: 4TPK) and *h*AChE (PDB code: 4M0F) (Brus et al., 2014; Liu et al., 2022). Given that the majority of synthesized tryptamine derivatives have significant inhibition efficacy on BuChE, we conducted a preliminary investigation into the action mode of various series of compounds (Figure 5). Docking poses indole fragments in compounds with 3- and 4-carbon chains (**2a** and **2b**, respectively) can form π–π stacking interactions and/or π–cation interactions with residues in the acyl-binding pocket (Trp231) and PAS (Phe329). Only stacking interactions with PAS (Tyr332, Phe329) or H-bond interactions with Ala328 can form the indole ring as increasing the alky carbon chain lengthens (**2c-2e**). The **2e**-bearing 7-carbon chain can form two stacking interactions with benzyl benzene and Trp231, but the indole fragment cannot form any interactions with the acyl-binding pocket or PAS in its structure. Furthermore, one of the benzyls in **2a** and **2d** can interact with PAS *via* an π–π stacking interaction (Tyr332). The slight difference is that **2b** and **3d** can form π–π stacking interaction with the choline-binding site (Trp82). However, the benzyl group in **2c** cannot form any interaction with active residues. Moreover, the tertiary amine nitrogen atom tends to form ions in the body and form H-bond interaction with part of the site cavity (Pro285), which can be observed in **2c**, **2e**, **2f**, and **2q**. By the way, due to the initial characteristic of the nitrogen atom, a similar phenomenon can also be observed in **3a-3d**, **3f**, and **3q**. All these results indicate that the variation of chain length has a certain effect on the action mode of compounds with BuChE. Notably, when one benzyl group is eliminated, the action mode is also changed. In contrast to **2a**, **3a**’s benzyl benzene can form stacking interactions with an acyl-binding pocket (Trp231) and a PAS (Phe329) rather than an indole fragment. Differently, **3c** bearing 5-carbon chain and **3d** bearing 6-carbon chain can form H-bond with acyl-binding pocket (Leu286) and π–π stacking interaction with PAS (Phe329) in their indole fragments, and their benzyl group can also form π–π stacking interactions with PAS (Tyr332 and Phe329). In addition, **3b** bearing 4-carbon chain and **3e** bearing 7-carbon chain can only form interactions with PAS. Thereinto, **3e** can also form H-bond with the key residue (His438) of the catalytic active site (CAS) and π–π stacking interactions with the choline-binding site (Trp82). When the terminal residue is a 3-carbon ring chain, the **2f** bearing dibenzyl and **3f** bearing monobenzyl groups all form π–π stacking interactions between the benzyl group and the acyl-binding pocket (Trp231) and PAS (Phe329), and the indole fragment with PAS (Tyr332). Furthermore, their indole fragment also can form π–π stacking interactions with the choline-binding site (Trp82). When the amino residue is relatively rigid alkyl amine, compounds bearing azetidine-based carbamate residue showed different modes of action. Among them, **2q** with dibenzyl groups can form H-bond interactions with acyl-binding pockets (Leu286) and π–π stacking interactions with PAS (Phe329), and benzyl groups can also form π–π stacking interactions with PAS (Tyr332). **3q** with a monobenzyl group, on the contrary, can only form π–π stacking interactions with acyl-binding pockets (Trp231) and PAS (Phe329) in its benzyl group. When the benzyl group is completely removed, **4e** in its indole fragment can form stacking interactions with the choline-binding site (Trp82) and H-bond interactions with CAS (His328). At the same time, it can form π–cation interaction and H-bond interaction with PAS (Phe329 and Thr120, respectively). All these results of the predicted action mode further indicate that the inhibition efficacy of this kind of compounds on BuChE is modulated by both the carbamate residues and carrier scaffolds. The subtle difference may be the key reason for their different inhibition potency because their different conformations have been varied.

**FIGURE 6:**
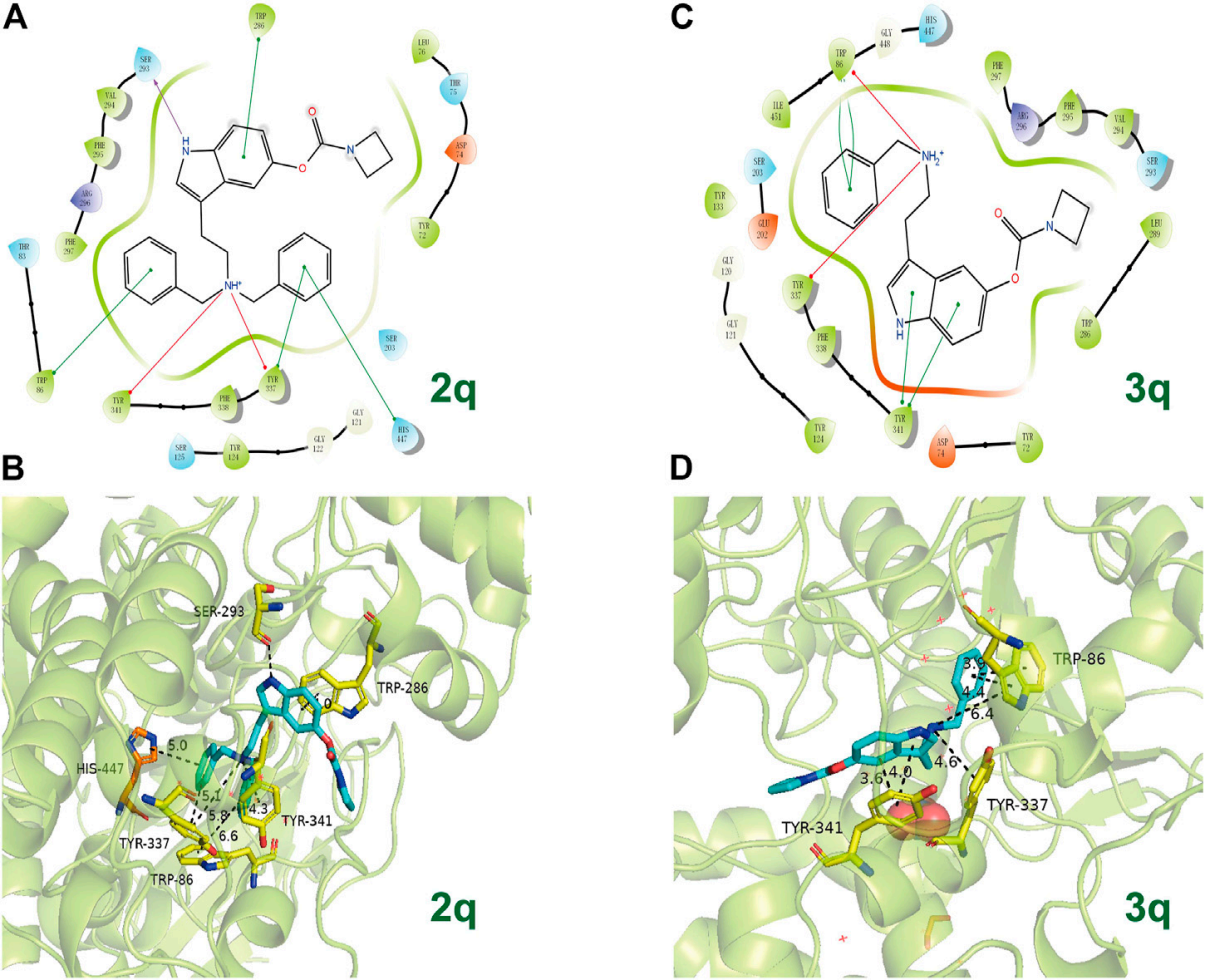
The possible binding mode for the compounds **2q** and **3q** in the AChE (PDB code: 4M0F) binding sites. **(A,C)** The 2D images of **2q** and **3q** binding to AChE predicted by Schrodinger software (Release 2019-2, Schrodinger, LLC, New York, NY, 2019). The purple arrow indicates hydrogen bond, the red arrow indicates π–cation interaction, and the green line indicates π–π stacking interaction. **(B,D)** The 3D images of **2q** and **3q** binding to AChE polished by Pymol (http://www.pymol.org). Residues of the CAS (His447) are shown in orange, and the residues of PAS (Ser293, Trp286, Tyr337, Trp86, and Tyr341) are shown in yellow. The dashed lines indicate hydrogen bond interaction, π–π stacking interactions, or π cation interactions.

In view of **2q** and **3q** bearing azetidine-based carbamate residue possessing better inhibition efficacy on AChE than other synthesized compounds, the action modes of **2q** and **3q** with AChE are preliminarily predicted by molecular docking (Figure 6). Docking poses of **2q** and **3q** with *h*AChE (PDB code: 4M0F) reveal that the compounds can form π–π stacking interactions with PAS (Trp86, Tyr341) in their benzyl group and indole fragment, as well as cation interactions with PAS (Tyr337) in their tertiary or secondary amine. Because the number of the containing benzyl group is different, one of the benzyl groups in **2q** can also form π–π stacking interaction with CAS (His447). However, the obtained IC_50_ value of **2q** is larger than that of **3q**. Given that the distances of π–π stacking interactions between compounds and PAS differ, we assume that more benzyl groups reduce the affinity of 2q to AChE, and research into the specific action mode of compounds on AChE is ongoing.

**FIGURE 7:**
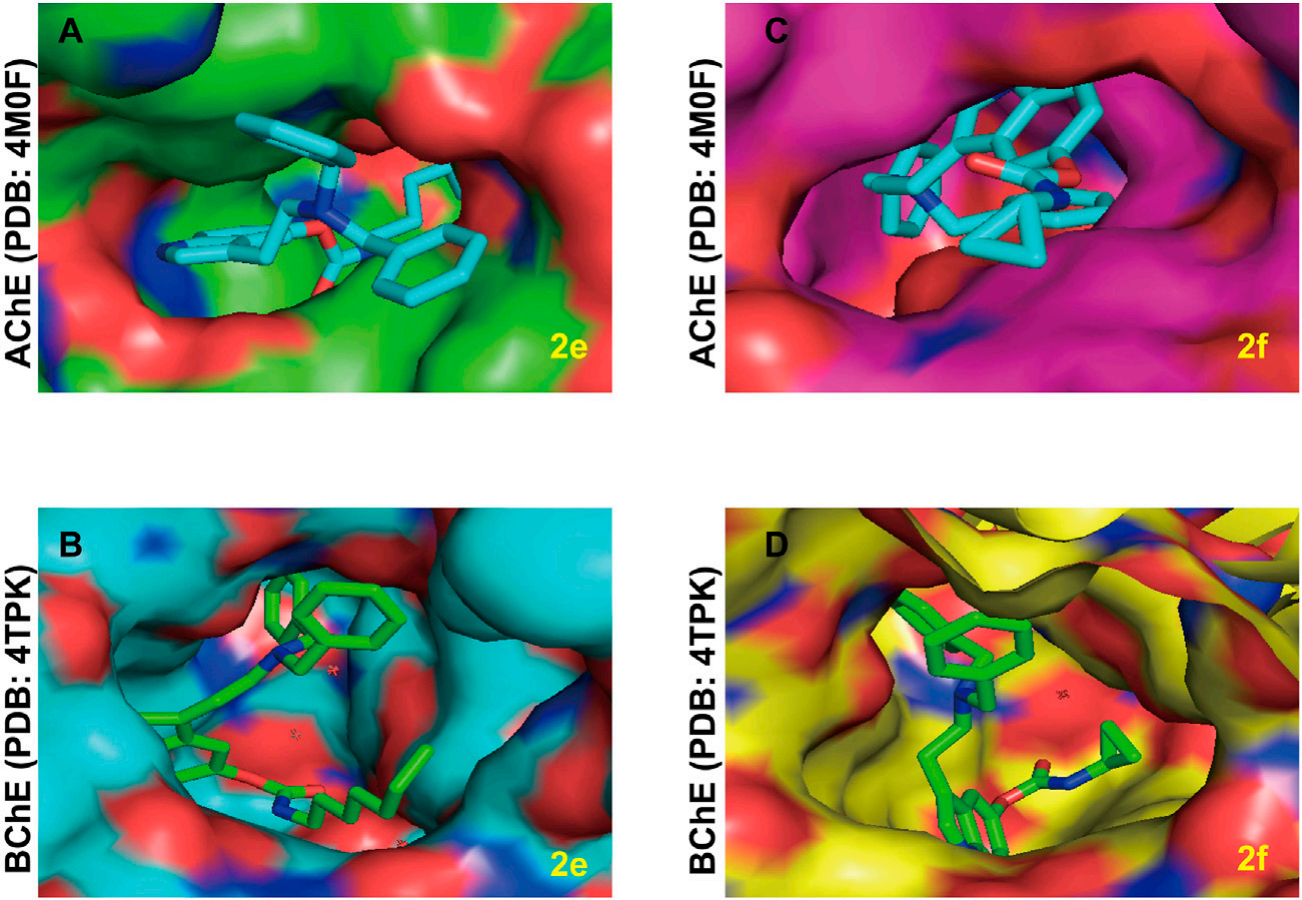
The possible binding mode for the compounds **2e** and **2f** in the AChE (PDB code: 4M0F) and BuChE (PDB code: 4TPK) binding sites. **(A,B)** The potential distribution surface diagrams of **2e** in AChE and BuChE binding sites**,** respectively, created by Pymol (http://www.pymol.org). **(C,D)** The potential distribution surface diagrams of **2f** in AChE and BuChE binding sites**,** respectively, created by Pymol (http://www.pymol.org).

According to previous studies, compounds with larger molecular shapes are preferred to bind to BuChE because of the larger size of the BuChE binding pocket compared to AChE (Sawatzky et al., 2016). In view of the curiosity about the selective BuChE inhibitory activity of the series of bisbenzyl-substituted CTDs, we explored the possible mode of action of **2e** and **2f** for AChE and BuChE (Figure 7). As expected, the bisbenzyl-substituted CTDs **2e** and **2f** cannot completely bind to the AChE binding pocket. Thereinto, the benzyl groups of **2e** and the carbamate residue of **2f** are distributed outside the active pocket due to the relatively small binding pocket of AChE and the relatively large molecular scaffold. Differently, due to the relatively large binding pocket of BuChE, the whole skeletons of **2e** and **2f** exhibit favorable affinity with BuChE active sites, which explains the possible mechanism of the selectivity obtained in the enzyme inhibition assay.

### Kinetic characteristic of BuChE inhibition

**FIGURE 8:**
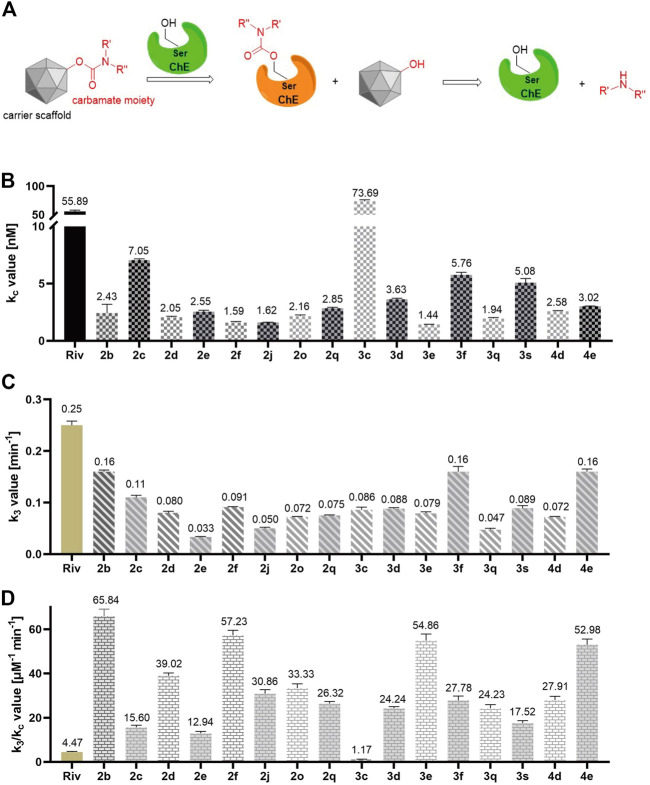
**(A)** The reported pseudo-irreversible inhibition mode of carbamylated derivatives on ChE. **(B)** The equilibrium constant (k_c_) values of tested compounds. **(C)** The carbamylation rate constant (k_3_) values of tested compounds. **(D)** The k_3_/k_c_ values of the tested compounds. All these data are the mean ± SD of triplicate in three independent experiments.

Convincing evidence has demonstrated that carbamylated derivatives with ChE inhibition efficacy exhibit a pseudo-irreversible inhibition mode (Figure 8A) (Hoffmann et al., 2019; Zhang et al., 2022b). Enlightened by this, we further conducted a kinetic study to explore the inhibition type of the synthesized CTDs, which possessed excellent inhibitory activity on BuChE. According to the results of the equilibrium constant (k_c_) for reversible combinations (Figure 8B), the carrier skeleton has a pronounced influence on the affinity between compounds and BuChE. Overall, compounds with monobenzyl substituents exhibit larger k_c_ values than those with dibenzyl groups, suggesting that the expansion of the carrier skeleton structure can decrease the affinity of compounds for BuChE. Combining the predicted action mode of these compounds with BuChE by molecular docking, the tryptamine skeleton is a key fragment that binds effectively to the enzyme. Besides, among the compounds with dibenzyl groups and compounds without benzyl group, the alkyl carbon chains in carbamate residues exhibit subtle influence for the affinity of compounds with enzyme, except for **2c** bearing a 5-carbon chain, which possesses stronger affinity than others in this series of compounds. Particularly, **3c** with the monobenzyl group shows a stronger affinity than that of rivastigmine. Furthermore, **3f** bearing 3-ring alkyl carbon chain and **3s** bearing piperidine-based carbamate residue have high affinity for BuChE. In addition, the results of the carbamylation rate constant (k_3_, Figure 8C) showed that the increasing carbon chain has a negative effect on carbamate transfer from the carrier scaffold to the enzyme in dibenzyl-substituted compounds **2b-2e**. However, there is no obvious effect of the carbamate residues in monobenzyl-substituted compounds **3c-3e** on k_3_. Compared with **2e** and **3e**, the benzyl-free compound **4e** bearing a 7-carbon side chain shows a significantly larger carbamate transfer rate, suggesting that the carrier scaffold and side residue of carbamate moiety synergistically determined the carbamoylation rate. Besides, the cyclopropanamine residues of carbamate moiety have a positive effect on the carbamoylation rate (**2f** and **3f**). Noteworthily, most of the tested compounds performed better affinity to BuChE and better carbamoylation efficacy because the k_3_/k_c_ value of compounds was greater than that of rivastigmine (Figure 8D). Therefore, this series of compounds is worthy of further study to perform structure optimization and excavate novel tryptamine-based MTDLs.

### Neuronal cytotoxicity assay

In order to explore the applicability of the synthesized CTDs in neurological diseases, the neuronal cytotoxicity of the synthesized compounds was evaluated on mouse hippocampal neuronal cell line HT-22. Table 3 shows that the neuronal cytotoxicity of compounds (**2a-2u**) bearing dibenzyl groups on HT-22 is lower than that of compounds (**3a-3u**) bearing monobenzyl group in general. The cytotoxicity of benzyl-free compounds (**4c-4e**) exhibited a large difference that the **4c** bearing 5-carbon alky chain residue showed little toxicity, but **4d** and **4e** bearing increasing carbon alkyl chain residues showed certain toxicity. In view of these CTDs endowed strong inhibition efficacy on BuChE, we preliminarily evaluated their safety range by comparing their IC_50_ values on cytotoxicity and BuChE inhibitory activity. The results showed that most of the tested compounds possessed considerable safety range, and only a few compounds (**3m** and **3n**) showed obvious toxicity. Therefore, the series of compounds with dibenzyl groups, especially compounds bearing ring carbon alkyl chains (**2f-2h**) and tertiary amine residues (**2j-2u**) with little cytotoxicity on HT-22, can be considered as potential lead building blocks for further structure optimization to develop novel effective agents against neurological diseases.

**TABLE 3:** Cytotoxicity of the synthesized compounds on HT-22 cell line.

Comp.	R	IC_50_ ± SD	Safety vs*.* BuChE^b^	Comp.	R	IC_50_ ± SD	Safety vs*.* BuChE^b^
		HT-22^a^ (μM)				HT-22^a^ (μM)	
**Don.**		56.42 ± 0.25	15	**3a**	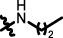	31.29 ± 2.32	1722
**Riv.**		>100	>431	**3b**	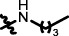	18.52 ± 0.81	782
**2a**	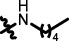	51.58 ± 0.05	1,554	**3c**	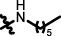	10.64 ± 0.24	445
**2b**	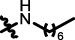	40.59 ± 1.12	13,530	**3d**	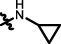	16.28 ± 1.19	856
**2c**	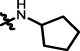	37.47 ± 0.23	17,842	**3e**	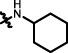	6.49 ± 0.34	193
**2d**	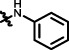	37.63 ± 1.92	25,598	**3f**	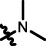	15.00 ± 1.17	953
**2e**	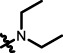	35.65 ± 2.45	25,105	**3g**	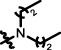	18.21 ± 0.95	1,340
**2f**	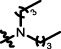	60.25 ± 3.85	6,886	**3h**	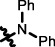	11.61 ± 0.34	266
**2g**	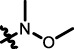	57.27 ± 4.30	1670	**3i**	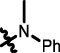	18.35 ± 0.43	166
**2h**	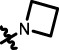	74.18 ± 1.50	4,642	**3j**	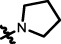	21.67 ± 0.72	165
**2i**	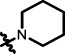	41.78 ± 2.31	749	**3k**	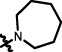	15.95 ± 0.39	220
**2j**	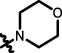	>100	>14,771	**3l**	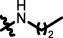	6.31 ± 0.39	144
**2k**	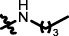	54.68 ± 0.42	170	**3m**	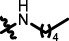	3.87 ± 0.35	40
**2l**	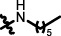	38.58 ± 0.90	2,426	**3n**	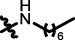	0.54 ± 0.03	3.5
**2m**	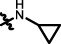	65.28 ± 0.09	4,723	**3o**	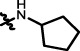	50.42 ± 1.07	857
**2n**	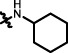	>100	381	**3p**	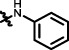	11.02 ± 1.12	433
**2o**	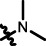	72.08 ± 2.23	8,333	**3q**	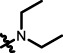	57.79 ± 2.88	22,399
**2p**	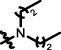	>100	5,305	**3r**	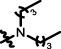	31.72 ± 1.64	783
**2q**	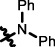	86.54 ± 1.82	52,448	**3s**	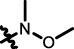	13.83 ± 0.91	408
**2r**	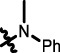	79.77 ± 5.36	3,897	**3t**	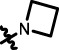	11.90 ± 0.39	512
**2s**	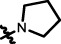	42.73 ± 2.09	1,281	**3u**	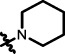	51.29 ± 2.55	457
**2t**	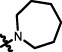	>100	1471	**4c**	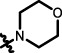	>100	>827
**2u**	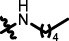	78.72 ± 3.79	688	**4d**	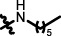	25.25 ± 1.37	912
				**4e**	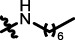	26.06 ± 2.29	2,593

Cell viability was examined by MTT assay.

^a^ IC_50_ values were calculated as the mean ± SD of triplicate in three independent experiments.

^b^ Safety vs*.* BuChE: IC_50_ (HT-22)/IC_50_ (BuChE). Don, donepezil. Riv, rivastigmine.

In order to further evaluate the applicability of the compounds with dibenzyl groups for neurological diseases, we also tested the cytotoxicity of the selected compounds (**2f**, **2h**, **2j**, **2m**, **2o-2r**, **2t**, and **2u**) bearing little toxicity on HT-22 cells, microglia cell line BV2, and human neuroblastoma cell line SH-SY5Y, which are widely used in the study of neurological diseases. Table 4 shows that all these tested compounds exhibited little or no toxicity on BV2 and SH-SY5Y cells, except for **2f** and **2q**, which exhibited certain toxicity. In view of some carbamate-based ChE inhibitors reported to be hepatotoxic, we further performed the preliminary evaluation of the hepatotoxicity of the selected compounds on human normal liver cell line LO2 and human hepatocellular carcinoma cell line HepG2. The results showed that all the tested compounds exhibited no hepatoxicity, suggesting that these CTDs can be safely used in drug discovery for further study.

**TABLE 4:** Cytotoxicity of the tested compounds on BV2, SH-SY5Y, LO2, and HepG2 cell lines.

Comp.	R	^a^IC_50_ ± SD (μM)			
		BV2	SH-SY5Y	LO2	HepG2
**Riv.**		>100	>100	>100	>100
**2f**	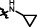	60.25 + 3.85	42.86 + 3.27	91.27 + 9.01	53.781 + 2.40
**2h**	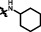	74.18 + 1.50	>100	>100	89.37 + 5.77
**2j**	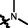	>100	>100	>100	>100
**2m**	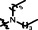	65.28 + 0.09	>100	>100	>100
**2o**	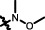	72.08 + 2.23	>100	74.44 + 4.26	95.23 + 0.22
**2p**	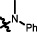	>100	>100	>100	>100
**2q**	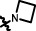	86.54 + 1.82	42.51 + 3.11	72.13 + 0.84	>100
**2r**	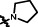	79.77 + 5.36	>100	>100	>100
**2t**	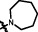	>100	98.68 + 4.82	>100	>100
**2u**	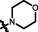	78.72 + 3.79	>100	>100	>100

Cell viability was examined by MTT assay.

^a^ IC_50_ values were calculated as the mean ± SD of triplicate in three independent experiments. Riv, rivastigmine.

### Neuroprotective effects of the selected compounds

**FIGURE 9:**
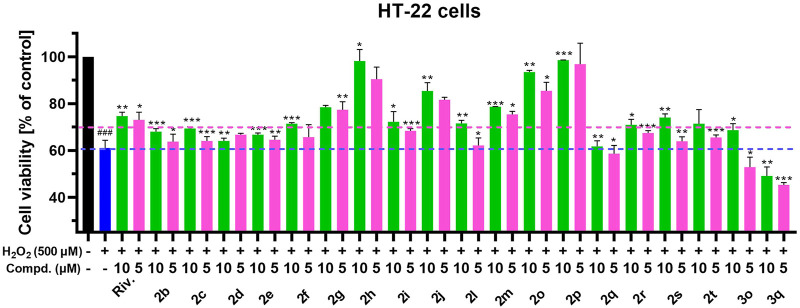
Cell viability was examined by MTT assay. Neuroprotective effects of tested compounds against the toxicity elicited by 500 μM H_2_O_2_. HT-22 cells were co-incubated with H_2_O_2_ and the tested compounds (5 and 10 μM) for 24 h. The values were expressed as mean ± SD of three independent experiments. ^*###*^ *p* < 0.001 compared to the control group. ***p < 0.001, **p < 0.01, or *p < 0.05 compared to the H_2_O_2_ induced model group. Riv, rivastigmine.

Neuronal damage caused by oxidative stress has been widely recognized in various neurological diseases. It is of great significance to protect neurons damaged by oxidative stress for the treatment of neurological diseases (Bai et al., 2022; Wakhloo et al., 2022). To date, many tryptamine derivatives have been reported to endow favorable neuronal protective functions (Hanikoglu et al., 2020; Forman and Zhang, 2021). Given this, we performed an elementary evaluation of neuronal protection against neuronal death elicited by H_2_O_2_ of the selected CTDs, which possessed little neuronal toxicity. Figure 9 shows that most of the tested compounds exhibited certain neuronal protection efficacy compared with the model group, in which the cell viability of the model group significantly decreased to 61.07% after treatment with H_2_O_2_ (500 μM). Among them, **2g**, **2h**, **2j**, **2m**, **2o**, and **2p** showed relatively favorable neuroprotective effects (the cell viability was more than 70% at 5 and 10 μM), so further structure optimization based on these compounds is of great value for the development of neuronal protective agents. By the way, the compounds bearing azetidine-based carbamate residues (**2q** and **3q**) exhibited no protective effects on H_2_O_2_-induced neuronal death. However, considering their extraordinary inhibitory activity on AChE and BuChE, this kind of compounds could be considered for drug development in peripheral cholinergic-related disease, which is ongoing in our current study.

### Evaluation of ORAC values of the selected compounds

**FIGURE 10:**
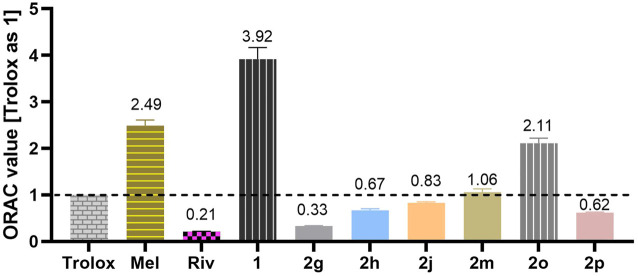
Results are expressed in the Trolox equivalents (TE) unit. The values were expressed as mean ± SD of three independent experiments.

Antioxidative effects are crucial in the treatment of many diseases (Ansari et al., 2020; Dumanovic et al., 2021). The Oxygen Radical Absorbance Capacity-Fluorescein (ORAC-FL) assay was used to preliminarily evaluate the antioxidant activities of the selected compounds (**2g**, **2h**, **2j**, **2m**, **2o**, and **2p**), which endowed favorable BuChE inhibition efficacy, little neuronal toxicity, and neuroprotective effects. The vitamin E analog Trolox was used as a standard, and its ORAC value was set as 1. Melatonin was used as a positive control with an ORAC value equal to 2.5^51^. At the same time, the non-carbamoylated compound **1** of the tested CTDs was also evaluated as a reference. As shown in Figure 10, non-carbamoylated compound **1** was endowed with favorable oxygen radical scavenging property with a Trolox equivalent value equal to 3.92. However, the carbamylated derivatives showed decreasing antioxidant activities, suggesting that the hydroxyl group played an important role in radical scavenging. Among them, **2o** bearing N-methoxymethylamine-based carbamate moiety exhibited a good antioxidant activity with Trolox equivalents value equal to 2.11, indicating that the special characteristic of methyl-methoxylamine fragment played certain modulation for the antioxidant activity. Besides, we also detected the inhibition efficacy of these compounds on NO production using the Griess reagent method. However, the results showed that most of this series of compounds had a weak inhibitory activity or even no effect, indicating that this series of compounds were insensitive to nitrogen radical.

### Inhibition assay on COX-2 of the selected compounds

**FIGURE 11:**
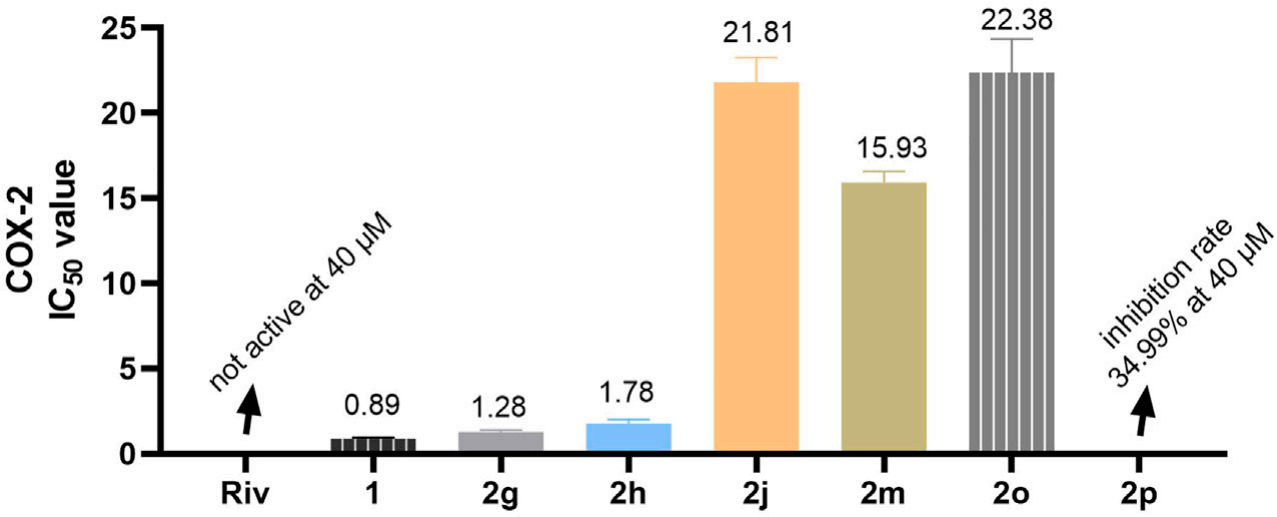
IC_50_ is the concentration (μМ) that causes 50% inhibition of COX-2 enzymatic activity. The values were expressed as mean ± SD of three independent experiments.

Glia cells play a significant role in neurological diseases (Zhang et al., 2021; Cai et al., 2022; Rauf et al., 2022). Microglia can release large amounts of COX-2 after being stimulated by inflammatory substances. Similarly, the expression of COX-1 in astrocytes was largely unchanged after stimulation, but the expression of COX-2 was significantly increased (Fan et al., 2021; Ghazanfari et al., 2021; Iwata et al., 2021; Nagano et al., 2021; Kuo et al., 2022). Therefore, inhibition of COX-2 expression in glia cells is of great value in the treatment of neurological diseases. The COX-2 inhibitory activity of the selected compounds was preliminarily evaluated using a commercial assay kit. As shown in Figure 11, **2g** and **2h** bearing cyclopentanamine-based carbamate residue and cyclohexanamine-based carbamate residue, respectively, had good COX-2 inhibition efficacy compared with the non-carbamylated compound **1**. However, other compounds bearing tertiary amine-based carbamate residues (**2j**, **2m**, **2o**, and **2p**) showed mild-to-weak inhibition efficacy on COX-2. By analyzing the structures of the tested compounds, we assumed that hydrogen bond interaction plays a certain role in the inhibitory activity of COX-2.

### Drug-like prediction of synthesized compounds

According to the drug discovery, “the rule of 5” predicts that the poor druggability of the compound is more likely to appear when the molecular weight (MWT) is greater than 500, calculated Log P (MLog P) is greater than 4.15, H-bond acceptors are greater than 10, H-bond donors are greater than 5, and the number of rotatable bonds is greater than 10 (Lipinski et al., 2022). Given this, the predicted druggability of synthesized compounds was calculated by the SwissADME. Table 5 shows that the MWT values of all the synthesized compounds were less than 500, except for **2m** and **2n**. The number of H-bond acceptors and H-bond donors of all these compounds was in accordance with Lipinski’s rule. Most compounds possessed favorable MLog P and BBB permeation properties. All these predicted results provide guidance for further study and suggest the potential value of the tryptamine derivatives.

**TABLE 5:** The predicted druggability of synthesized compounds.

Comp.	MWT	MLog P	HA	HD	BBB	Comp.	MWT	MLog P	HA	HD	BBB
**2a**	441.56	3.89	3	2	No	**3c**	379.50	3.08	3	3	Yes
**2b**	455.59	4.08	3	2	No	**3d**	393.52	3.30	3	3	Yes
**2c**	469.62	4.28	3	2	No	**3e**	407.55	3.50	3	3	No
**2d**	483.64	4.47	3	2	No	**3f**	349.43	2.65	3	3	Yes
**2e**	497.67	4.66	3	2	No	**3g**	377.48	3.08	3	3	Yes
**2f**	439.55	3.89	3	2	Yes	**3h**	391.51	3.30	3	3	Yes
**2g**	467.60	4.28	3	2	No	**3i**	385.46	3.35	3	3	Yes
**2h**	481.63	4.47	3	2	No	**3j**	337.42	2.43	3	2	Yes
**2i**	475.58	4.53	3	2	No	**3k**	365.47	2.87	3	2	Yes
**2j**	427.54	3.69	3	1	Yes	**3l**	393.52	3.30	3	2	Yes
**2k**	455.59	4.08	3	1	No	**3m**	421.58	3.71	3	2	No
**2l**	483.64	4.47	3	1	No	**3n**	461.55	4.61	3	2	No
**2m**	511.70	4.84	3	1	No	**3o**	353.41	2.43	4	2	Yes
**2n**	551.68	5.69	3	1	No	**3p**	399.48	3.55	3	2	Yes
**2o**	443.54	3.69	4	1	Yes	**3q**	349.43	2.65	3	2	Yes
**2p**	489.61	4.72	3	1	No	**3r**	363.45	2.87	3	2	Yes
**2q**	439.55	3.89	3	1	Yes	**3s**	377.48	3.08	3	2	Yes
**2r**	453.58	4.08	3	1	Yes	**3t**	391.51	3.30	3	2	Yes
**2s**	467.60	4.28	3	1	Yes	**3u**	379.45	2.06	4	2	Yes
**2t**	481.63	4.47	3	1	No	**4c**	289.37	1.72	3	2	No
**2u**	469.57	3.27	4	1	Yes	**4d**	303.40	1.96	3	3	No
**3a**	351.44	2.65	3	3	Yes	**4e**	317.43	2.20	3	3	No
**3b**	365.47	2.87	3	3	Yes						

Lipinski’s rules: MWT < 500; MW: MLog *p* < 4.15; HA < 10; HD < 5. MWT, molecular weight; MLog P, Log *P*_*o/w*_; HA, H-bond acceptors; HD, H-bond donors; BBB, BBB permeant. All these parameters were predicted by http://www.swissadme.ch/.

## Conclusion

In this study, we designed and synthesized a novel series of benzyl-free, monobenzyl-, and bisbenzyl-substituted tryptamine derivatives bearing functional carbamate groups to explore promising building blocks for the discovery of efficient MTDLs against ChE-associated neurological disorders. The ChE inhibition assay revealed that the majority of these hybrids had good-to-excellent BuChE inhibitory activities. In particular, several dibenzyl-substituted CTDs (**2b-2f**, **2j**, **2o**, and **2q**) possessed excellent BuChE inhibition efficacy with IC_50_ values in a single-digit nanomolar level. A molecular docking study on BuChE showed that the bisbenzyl-substituted CTDs could interact with the key residues of active binding sites, and the kinetic study indicated that the tested compounds performed pseudo-irreversible inhibition models. The *in vitro* neuronal cytotoxicity assay suggested that **2g**, **2h**, **2j**, **2m**, **2o**, and **2p** showed favorable neuroprotective potency on H_2_O_2_-induced HT-22 cells. The ORAC assay and COX-2 inhibition screening assay indicated that **2g**, **2h**, **2j**, **2m**, **2o**, and **2p** were also endowed with good antioxidant activities and COX-2 inhibitory effects. In view of the overall promising findings mentioned above, this kind of CTDs is used as the lead scaffold for further structural modification to develop efficient MTDLs agents against ChE-associated neurodegenerative disorders, such as AD and PD.

### Associated content

The spectral data and additional experimental information, except for those stated above, are presented in the Supplementary Material.

## Data Availability

The original contributions presented in the study are included in the article/Supplementary Material. Further inquiries can be directed to the corresponding authors.
