# Supplementary material for: From tryptamine to the discovery of efficient multi-target directed ligands against cholinesterase-associated neurodegenerative disorders
Source: Front Pharmacol. 2022 Nov 28;13:1036030. doi: 10.3389/fphar.2022.1036030 (PMC9742383; doi:10.3389/fphar.2022.1036030)
Supplement: Supplementary file 1 [file DataSheet1.PDF]

Supporting Information

From Tryptamine to Discovery Efficient Multi-Target Directed Ligands Against Cholinesterase-Associated Neurodegenerative Disorders

Junbo Wu[1,6§], Honghua Zhang[2§], Yuying Wang[4], Gaofeng Yin[5], Qien Li[7], Linsheng Zhuo[2*], Hongjin Chen[1*], Zhen Wang[2, 3*]

[1]Department of Colorectal Surgery, The Affiliated Hospital, Nanjing University of Chinese Medicine, Nanjing, Jiangsu, 210029, China.

[2]School of Pharmaceutical Science, Hengyang Medical School, University of South China, Hengyang, Hunan, 421001, China

[3]The First Affiliated Hospital, Hengyang Medical School, University of South China, Hengyang, Hunan 421001, China

[4]State Key Laboratory of Applied Organic Chemistry, College of Chemistry and Chemical Engineering, Lanzhou University, Lanzhou 730000, China

[5]School of Pharmacy, Lanzhou University, Lanzhou, 730000, China

[6]Department of Colorectal Surgery, Hengyang Central Hospital, Hengyang, Hunan, 410000, China

[7]Tibetan Medical College, Qinghai University, Xining 810016, Qinghai, China

[§]Wu JB and Zhang HH contributed equally to this work.

***Correspondence:**

Zhen Wang, Linsheng Zhuo, Hongjin Chen

zhenw@lzu.edu.cn; lszhuo@mail.ccnu.edu.cn; 260789@njucm.edu.cn

Table of Contents

^{1}H NMR and ^{13}C NMR of synthesized compounds

HPLC results of synthesized compounds

HRMS spectras of synthesized compounds

Molecular Formula Strings of synthesized compounds

^{1}H NMR and ^{13}C NMR of synthesized compounds

2a:

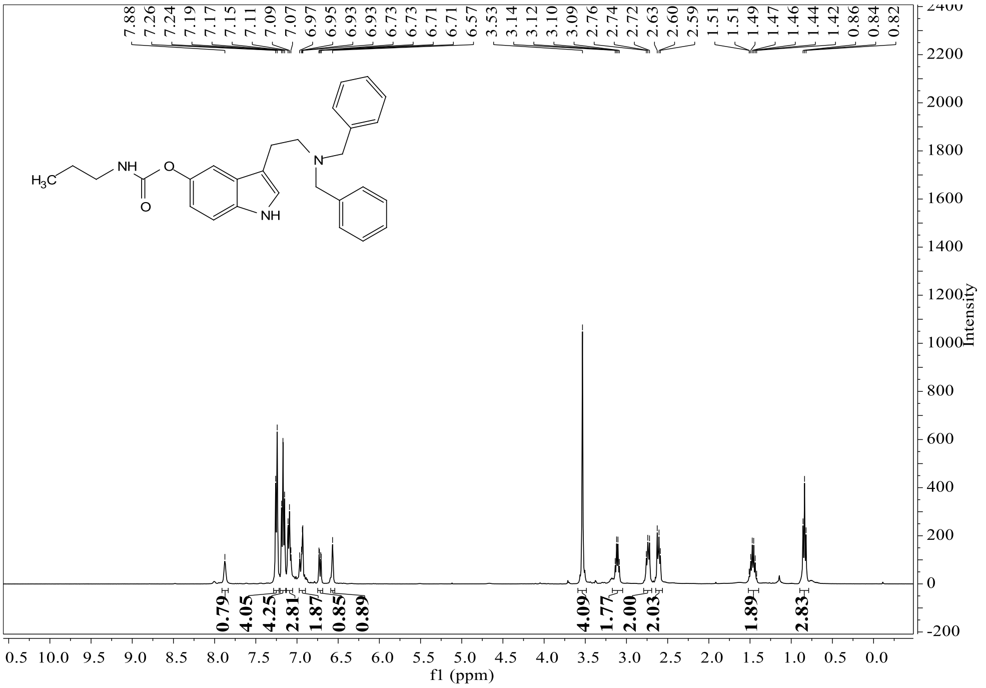

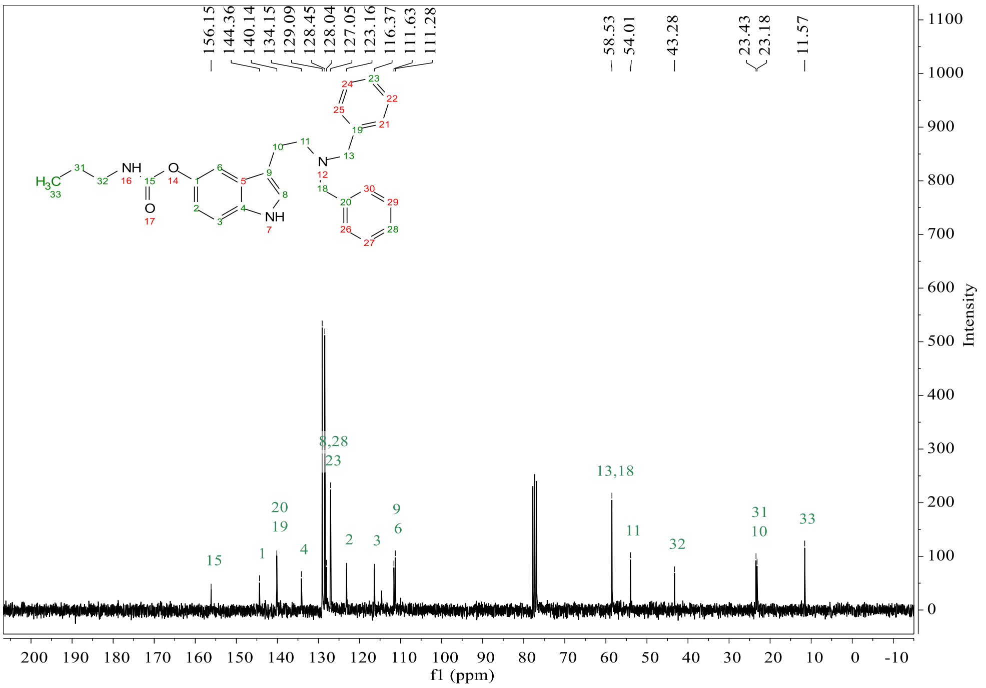

2b:

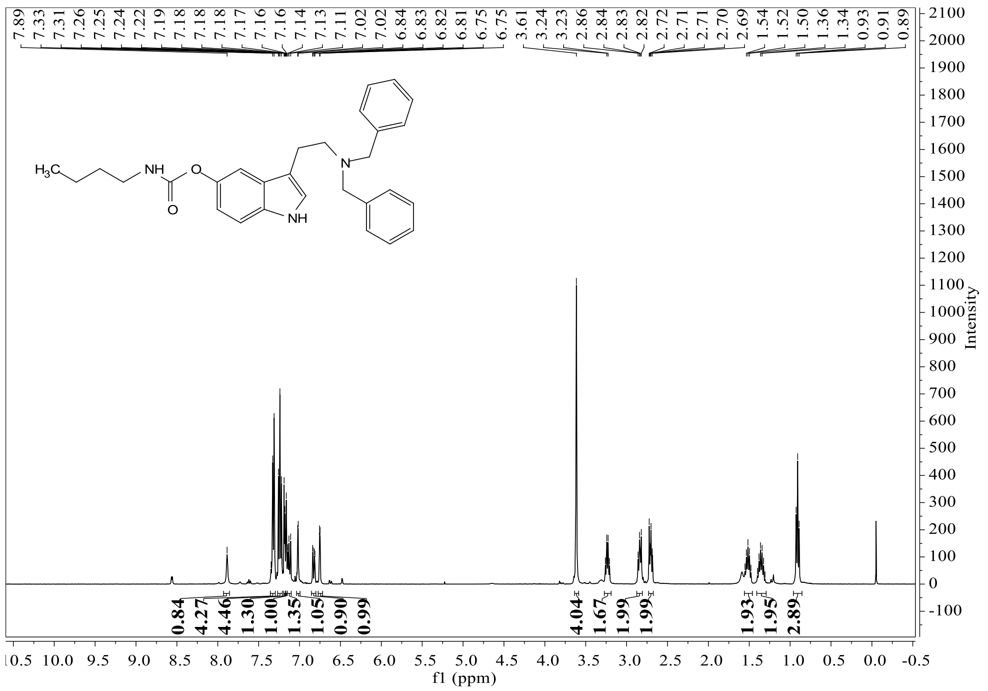

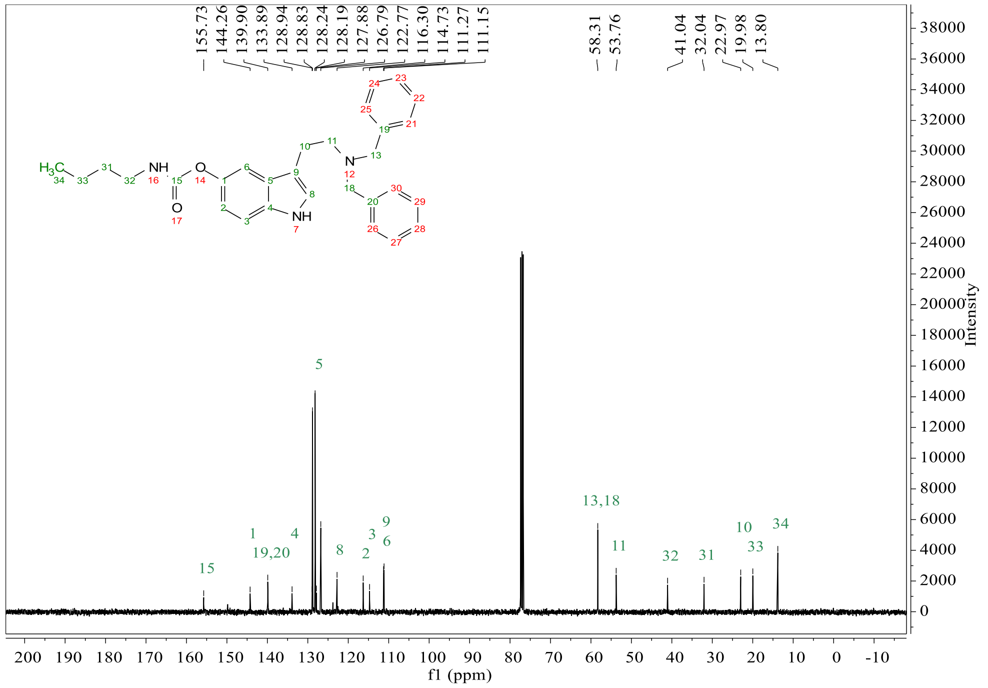

2c:

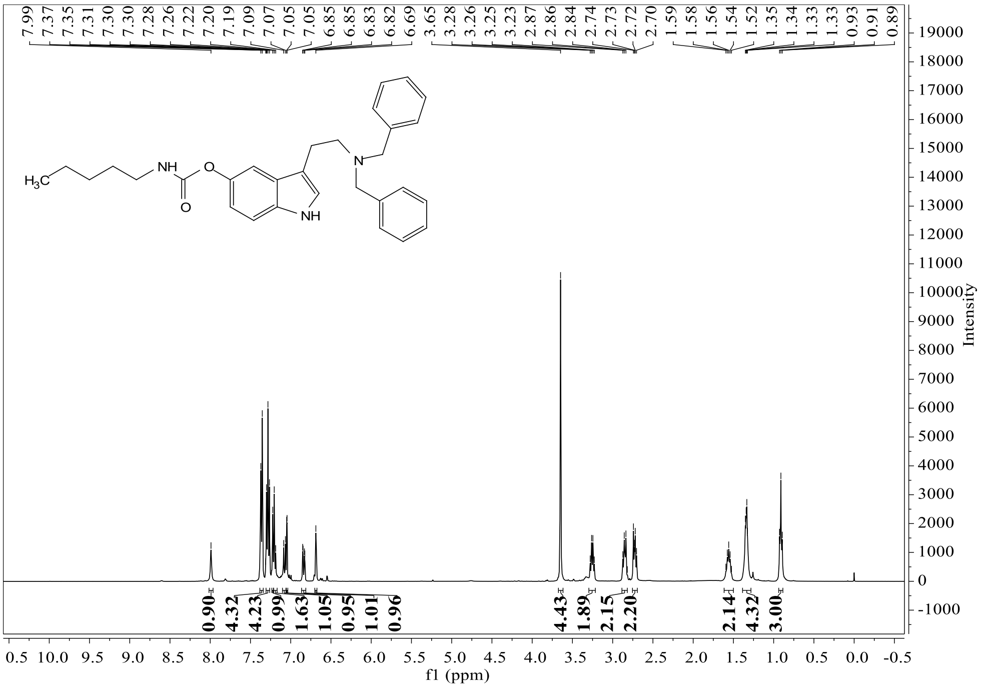

7.99
7.37
7.35
7.31
7.30
7.30
7.28
7.26
7.22
7.20
7.19
7.09
7.07
7.05
7.05
6.85
6.85
6.83
6.82
6.69
3.65
3.28
3.26
3.25
3.23
2.87
2.86
2.84
2.74
2.73
2.72
2.70
1.59
1.58
1.56
1.54
1.52
1.35
1.34
1.33
1.33
0.93
0.91
0.89
NH
O
H3C
N
NH
0.90
4.32
4.23
0.99
1.63
1.05
0.95
1.01
0.96
4.43
1.89
2.15
2.20
2.14
4.32
3.00
Intensity
f1 (ppm)

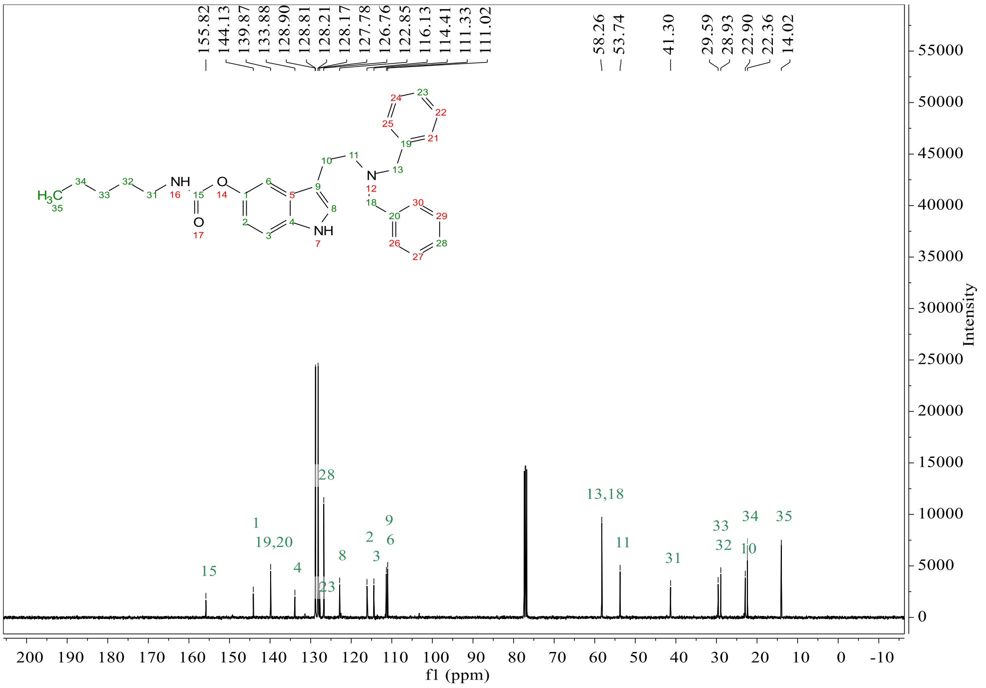

155.82
144.13
139.87
133.88
128.90
128.81
128.21
128.17
127.78
126.76
122.85
116.13
114.41
111.33
111.02
58.26
53.74
41.30
29.59
28.93
22.90
22.36
14.02
15
1
19,20
4
28
23
8
2
3
9
6
13,18
11
31
33
32
34
10
35
Intensity
f1 (ppm)

2d:

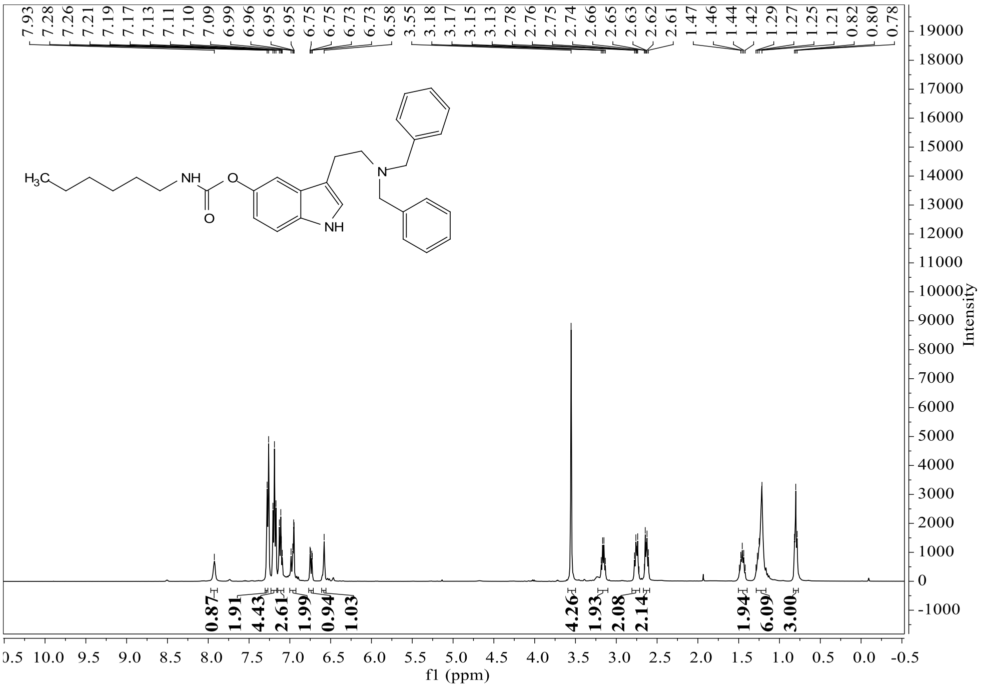

7.93
7.28
7.26
7.21
7.19
7.17
7.13
7.11
7.10
7.09
6.99
6.96
6.95
6.95
6.75
6.75
6.73
6.73
6.58
3.55
3.18
3.17
3.15
3.13
2.78
2.76
2.75
2.74
2.66
2.65
2.63
2.62
2.61
1.47
1.46
1.44
1.42
1.29
1.27
1.25
1.21
0.82
0.80
0.78
H3C
NH
O
O
N
NH
0.87
1.91
4.43
2.61
1.99
0.94
1.03
4.26
1.93
2.08
2.14
1.94
6.09
3.00
f1 (ppm)
Intensity

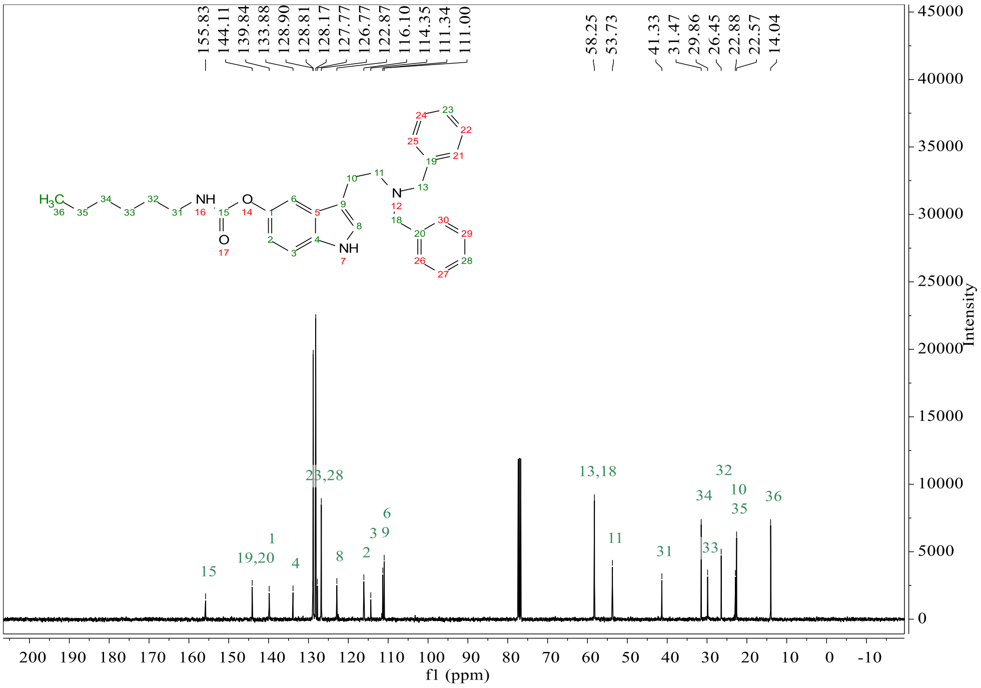

155.83
144.11
139.84
133.88
128.90
128.81
128.17
127.77
126.77
122.87
116.10
114.35
111.34
111.00
58.25
53.73
41.33
31.47
29.86
26.45
22.88
22.57
14.04
15
19,20
1
4
23,28
8
2
3
9
6
13,18
11
31
33
34
32
10
35
36
f1 (ppm)
Intensity

2e:

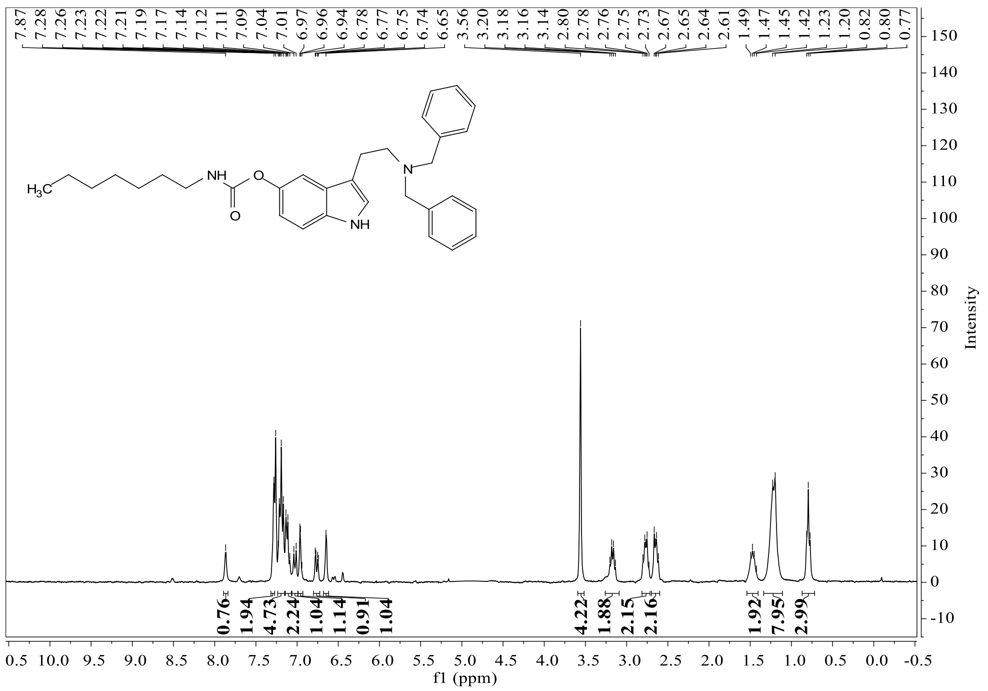

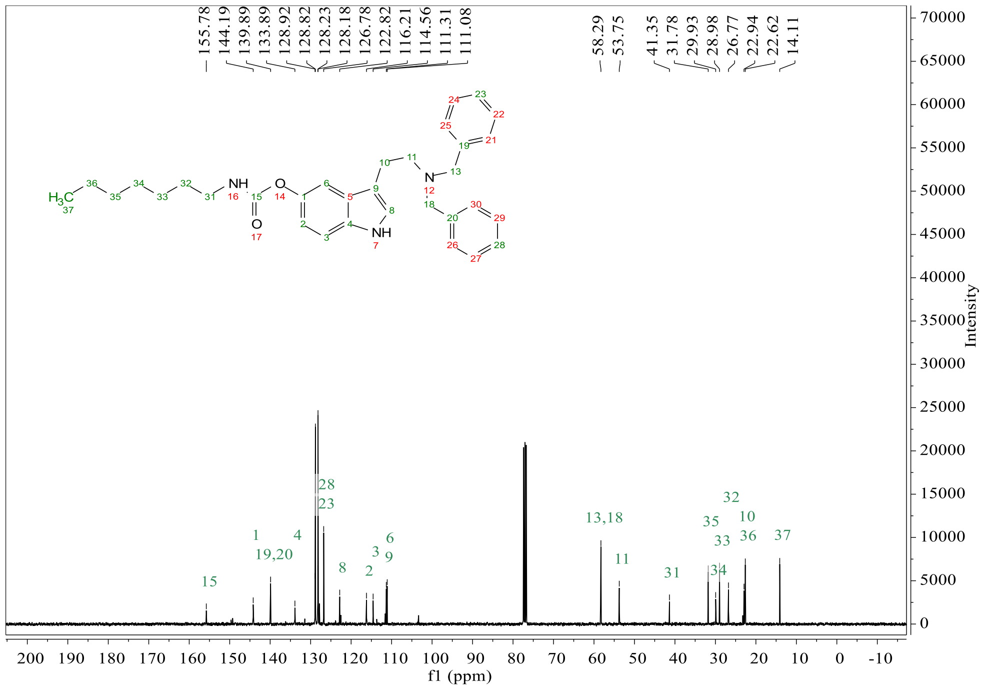

2f:

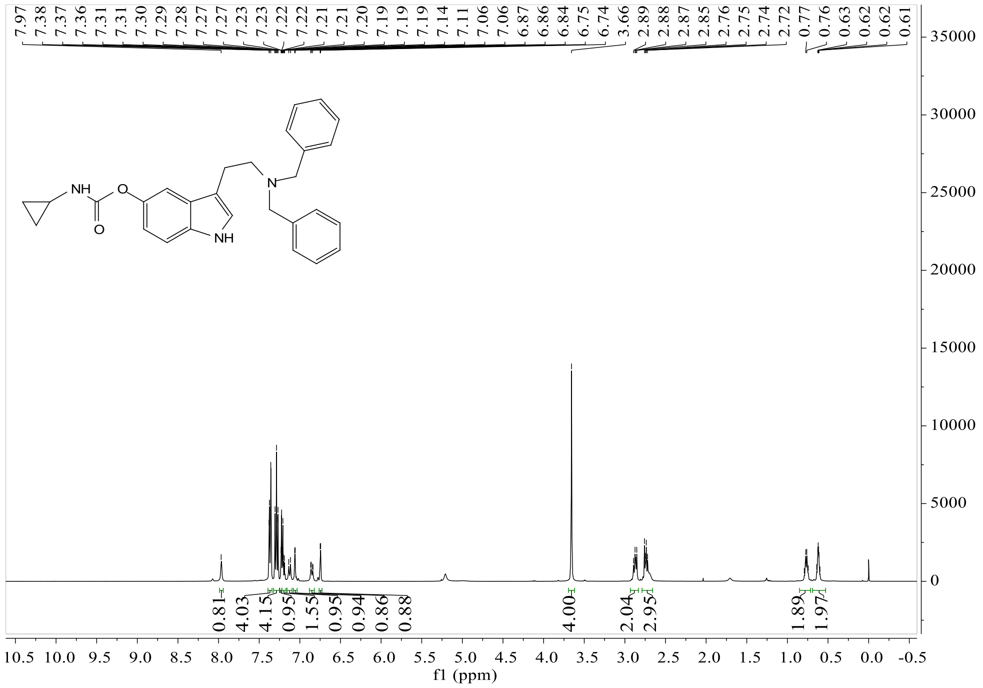

7.97
7.38
7.37
7.36
7.31
7.31
7.30
7.29
7.28
7.27
7.27
7.23
7.23
7.22
7.22
7.21
7.21
7.20
7.19
7.19
7.19
7.14
7.11
7.06
7.06
6.87
6.86
6.84
6.75
6.74
3.66
2.89
2.88
2.87
2.85
2.76
2.75
2.74
2.72
0.77
0.76
0.63
0.62
0.62
0.61
NH
O
O
N
NH
0.81
4.03
4.15
0.95
1.55
0.95
0.94
0.86
0.88
4.00
2.04
2.95
1.89
1.97
f1 (ppm)

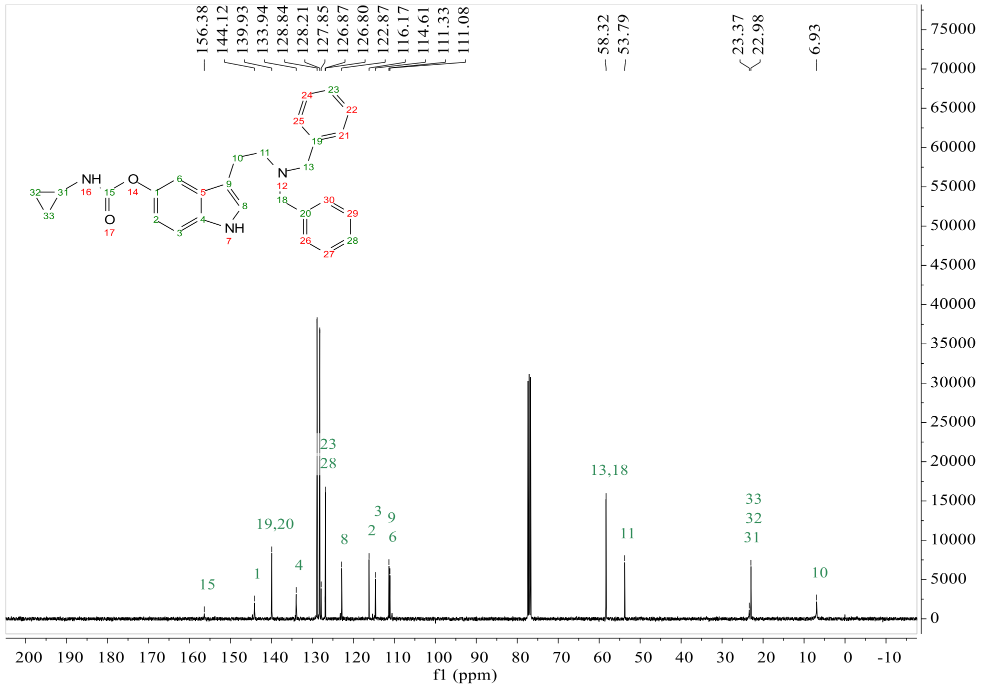

156.38
144.12
139.93
133.94
128.84
128.21
127.85
126.87
126.80
122.87
116.17
114.61
111.33
111.08
58.32
53.79
23.37
22.98
6.93
15
1
19,20
4
23
28
8
2
3
9
6
13,18
11
33
32
31
10
f1 (ppm)

2g:

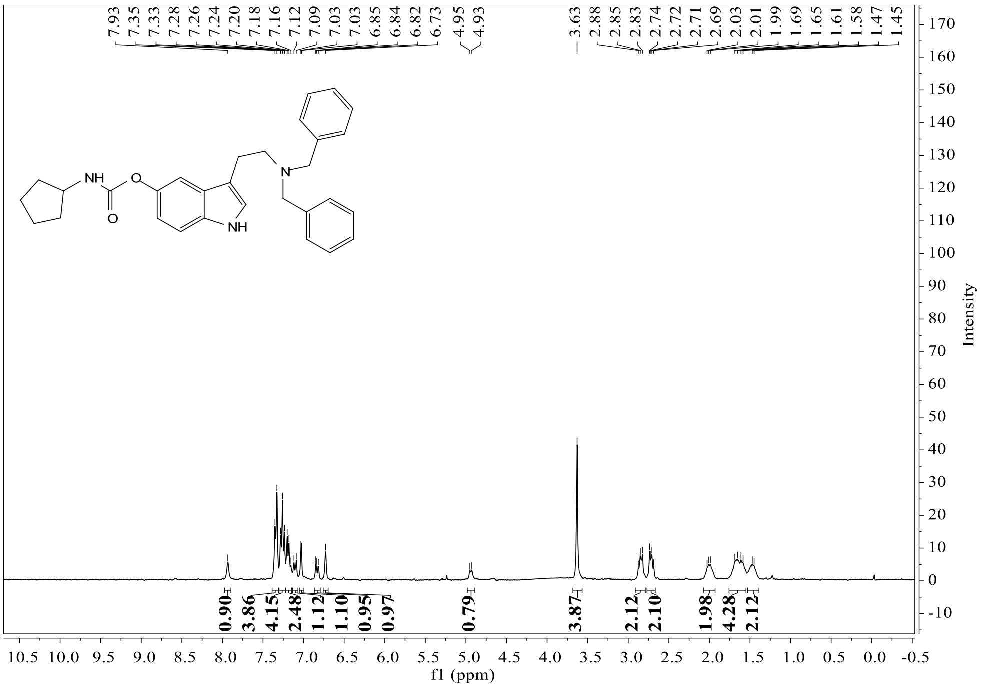

7.93
7.35
7.33
7.28
7.26
7.24
7.20
7.18
7.16
7.12
7.09
7.03
7.03
6.85
6.84
6.82
6.73
4.95
4.93
3.63
2.88
2.85
2.83
2.74
2.72
2.71
2.69
2.03
2.01
1.99
1.69
1.65
1.61
1.58
1.47
1.45
NH
O
O
N
NH
0.90
3.86
4.15
2.48
1.12
1.10
0.95
0.97
0.79
3.87
2.12
2.10
1.98
4.28
2.12
f1 (ppm)
Intensity

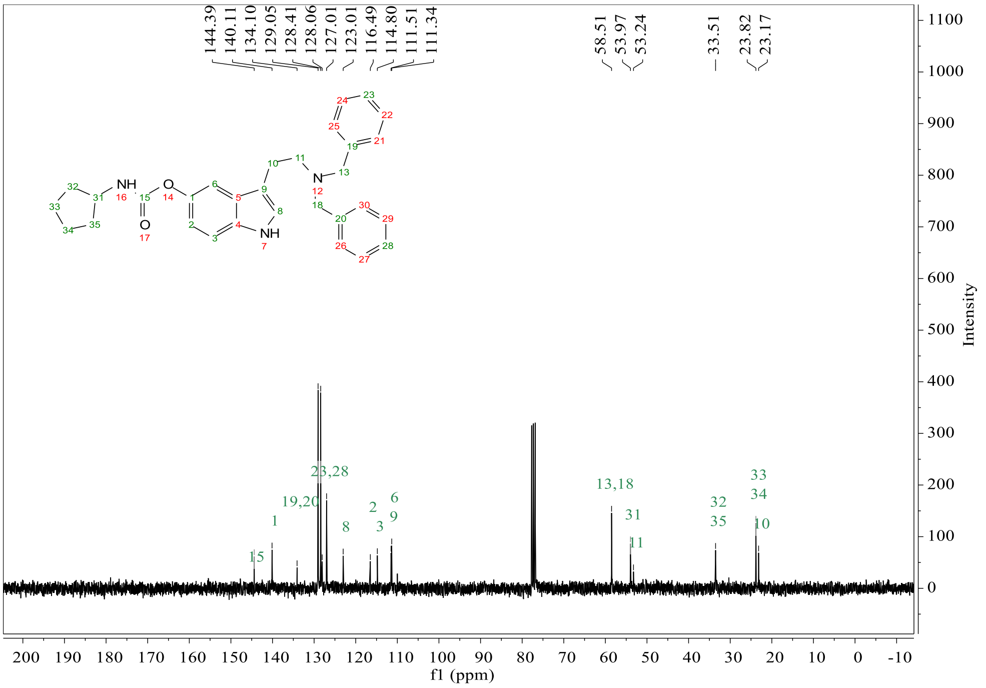

144.39
140.11
134.10
129.05
128.41
128.06
127.01
123.01
116.49
114.80
111.51
111.34
58.51
53.97
53.24
33.51
23.82
23.17
15
1
19,20
23,28
8
2
3
6
9
13,18
31
11
32
35
33
34
10
f1 (ppm)
Intensity

2h:

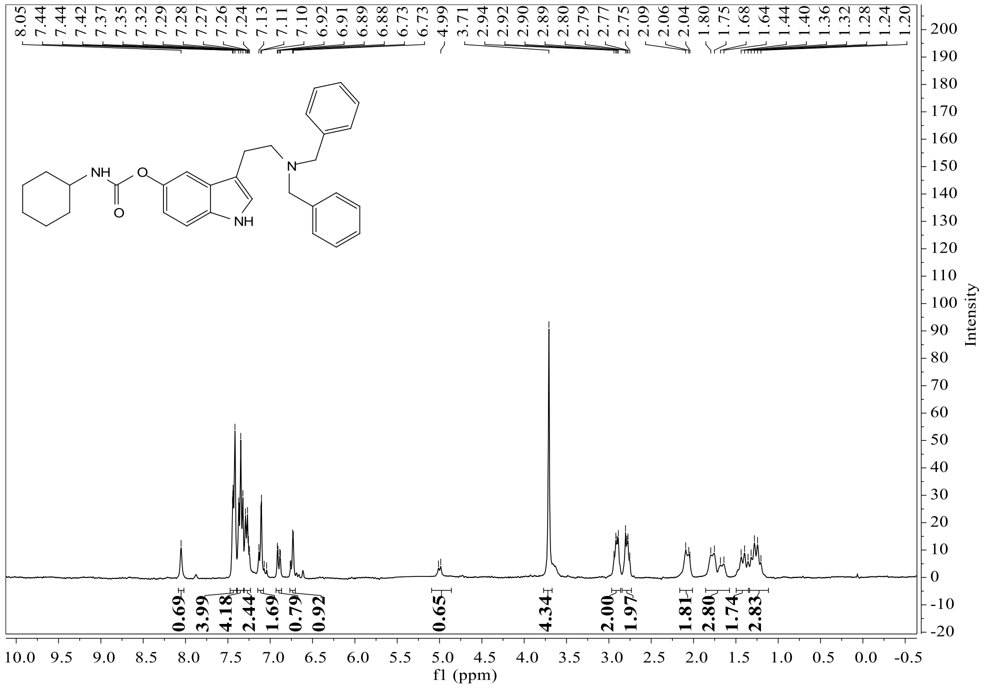

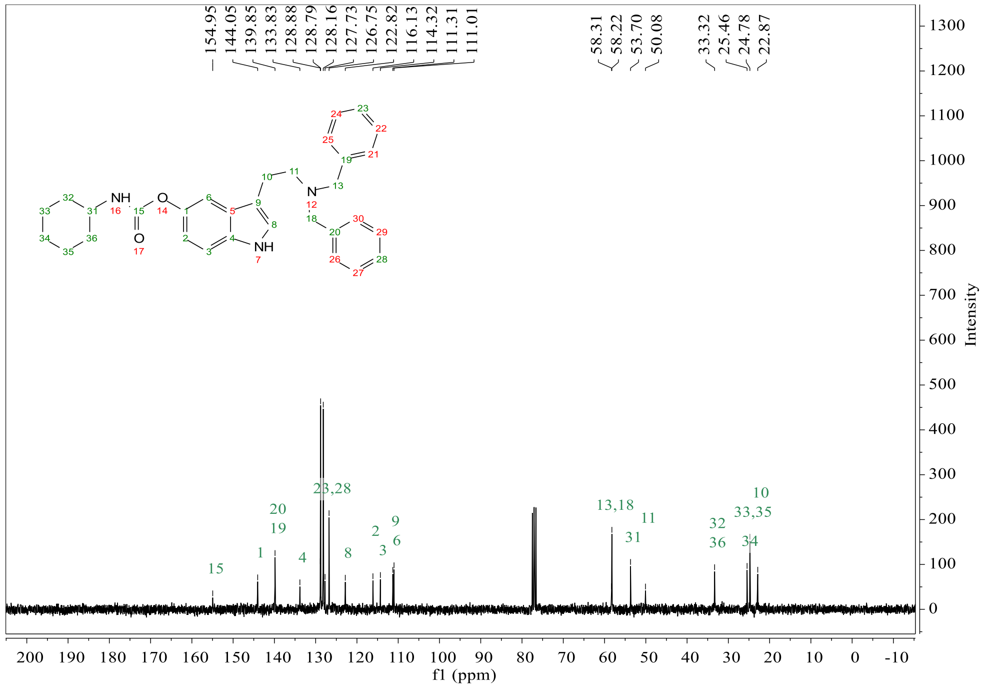

2i:

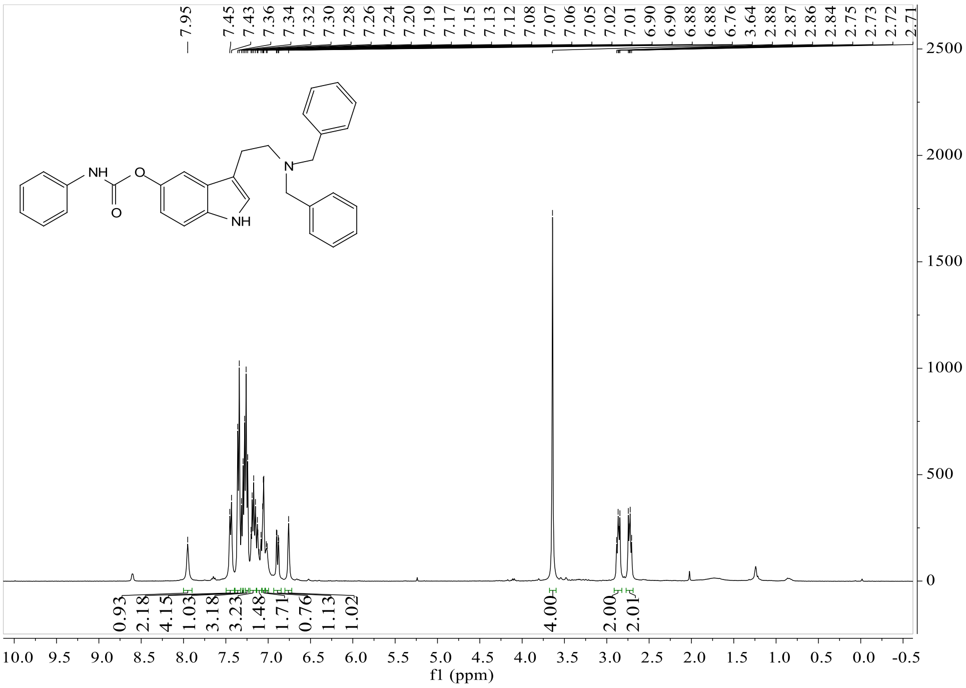

7.95
7.45 7.43 7.36 7.34 7.32 7.30 7.28 7.26 7.24 7.20 7.19 7.17 7.15 7.13 7.12 7.08 7.07 7.06 7.05 7.02 7.01 6.90 6.90 6.88 6.88 6.76 3.64 2.88 2.87 2.86 2.84 2.75 2.73 2.72 2.71
NH
O
O
N
NH
0.93 2.18 4.15 1.03 3.18 3.23 1.48 1.71 0.76 1.13 1.02
4.00
2.00 2.01
f1 (ppm)

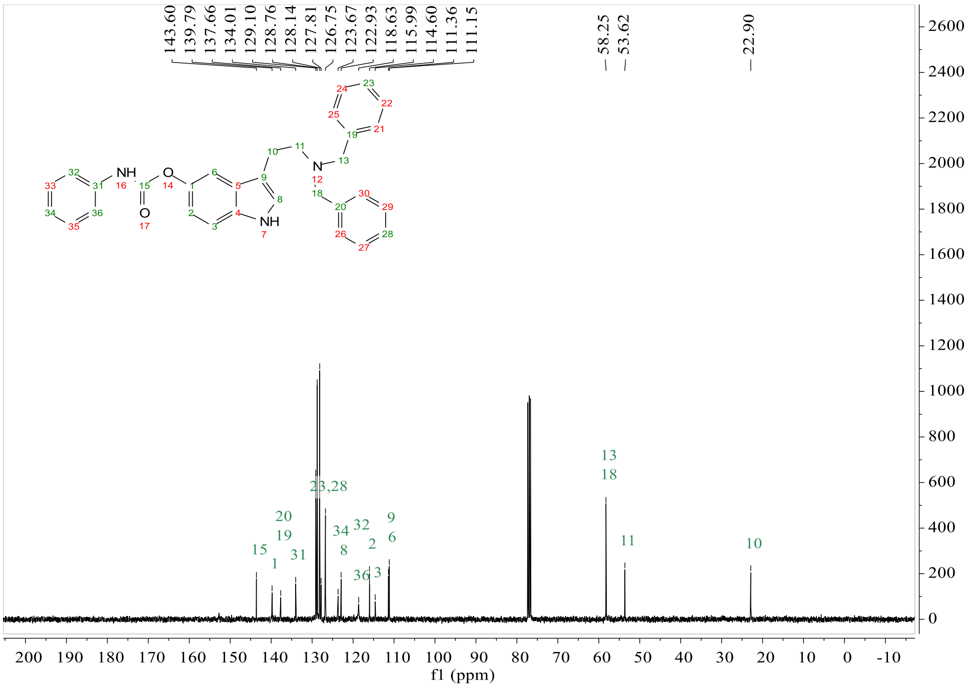

143.60 139.79 137.66 134.01 129.10 128.76 128.14 127.81 126.75 123.67 122.93 118.63 115.99 114.60 111.36 111.15
58.25 53.62
22.90
13
18
11
10
23,28
20
19
15
1
31
34
8
32
2
9
6
36
3
f1 (ppm)

2j:

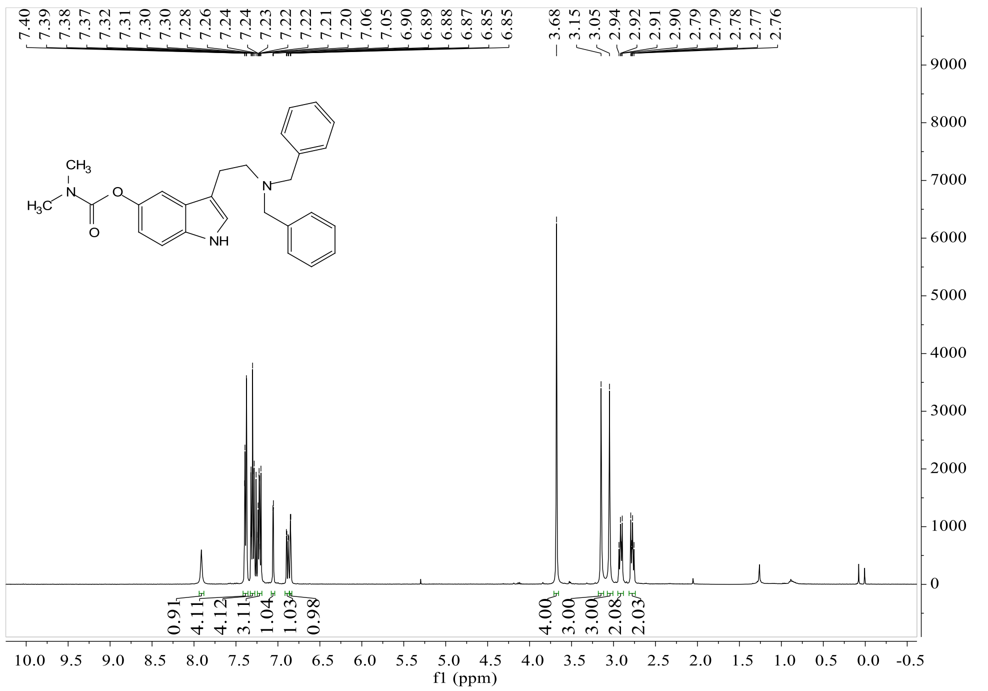

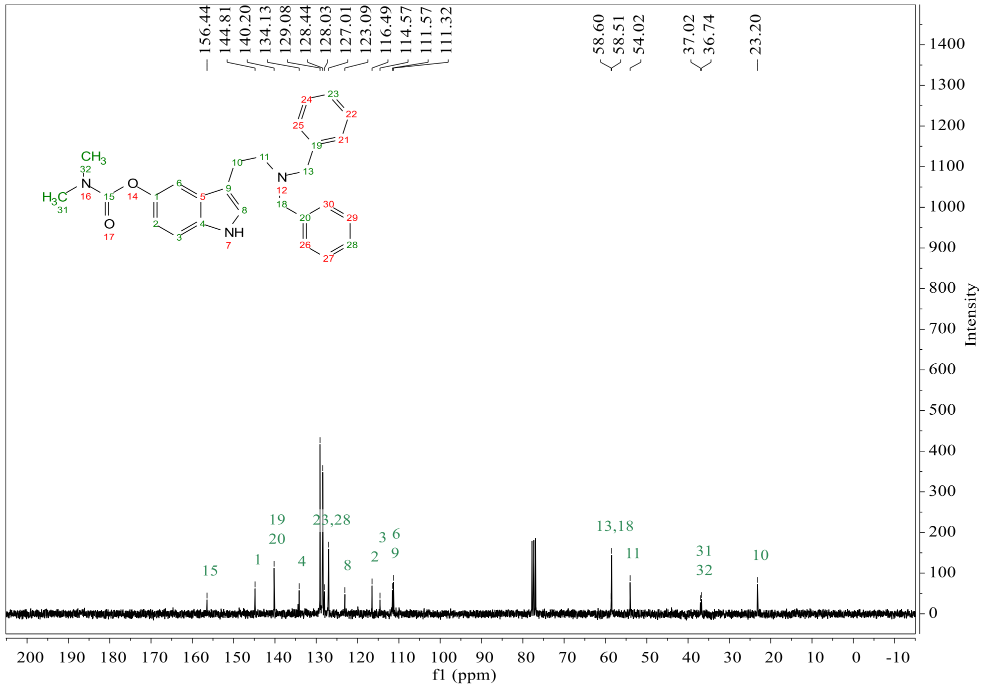

2k:

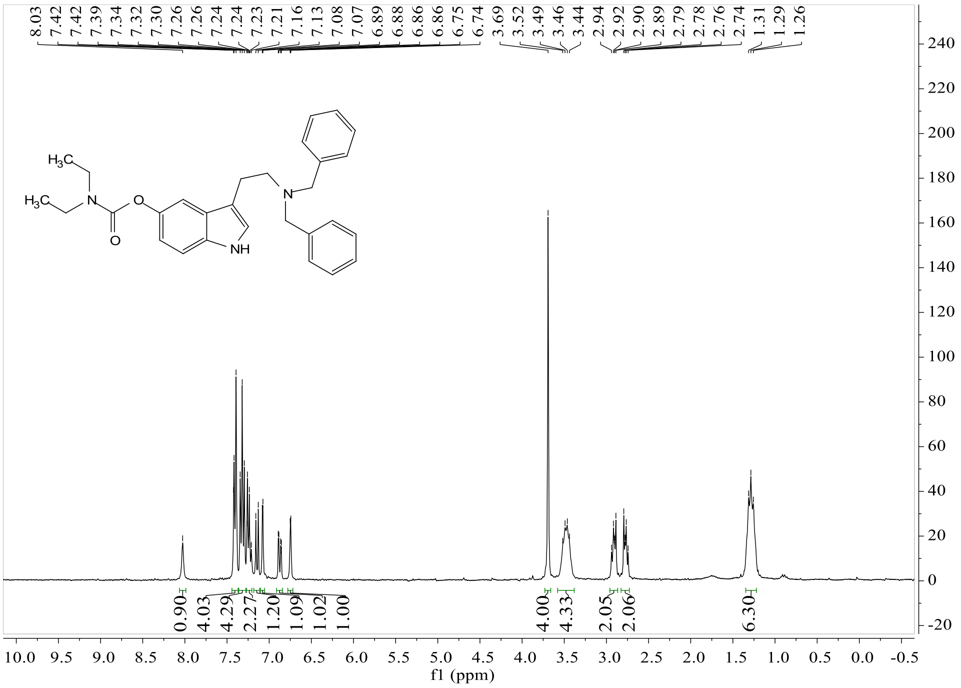

8.03 7.42 7.42 7.39 7.34 7.32 7.30 7.26 7.26 7.24 7.24 7.23 7.21 7.16 7.13 7.08 7.07 6.89 6.88 6.86 6.86 6.75 6.74 3.69 3.52 3.49 3.46 3.44 2.94 2.92 2.90 2.89 2.79 2.78 2.76 2.74 1.31 1.29 1.26
0.90 4.03 4.29 2.27 1.20 1.09 1.02 1.00 4.00 4.33 2.05 2.06 6.30
f1 (ppm)

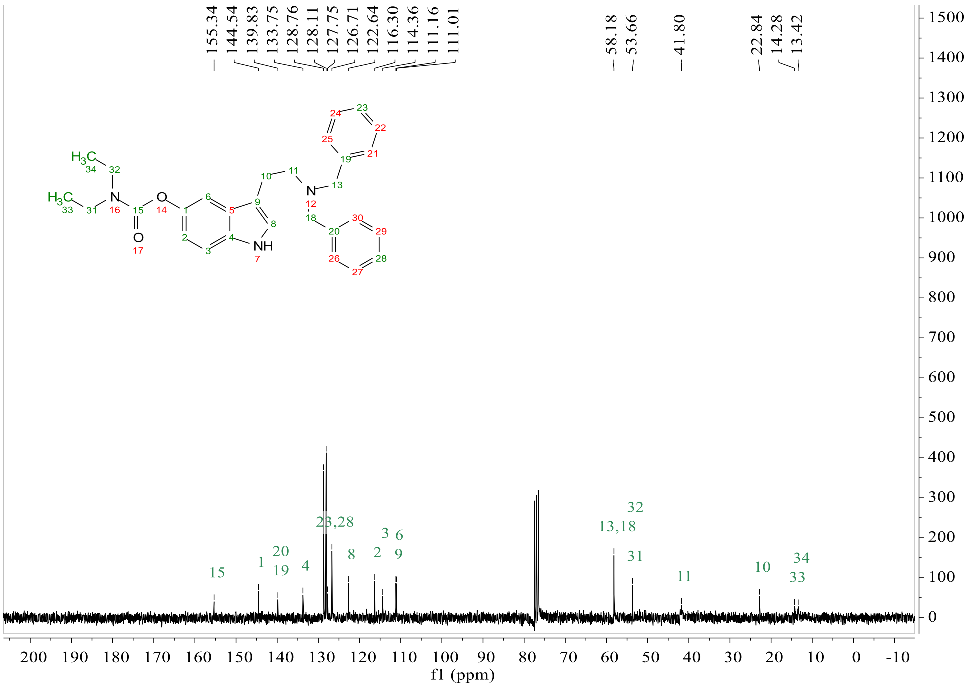

155.34 144.54 139.83 133.75 128.76 128.11 127.75 126.71 122.64 116.30 114.36 111.16 111.01 58.18 53.66 41.80 22.84 14.28 13.42
f1 (ppm)

2l:

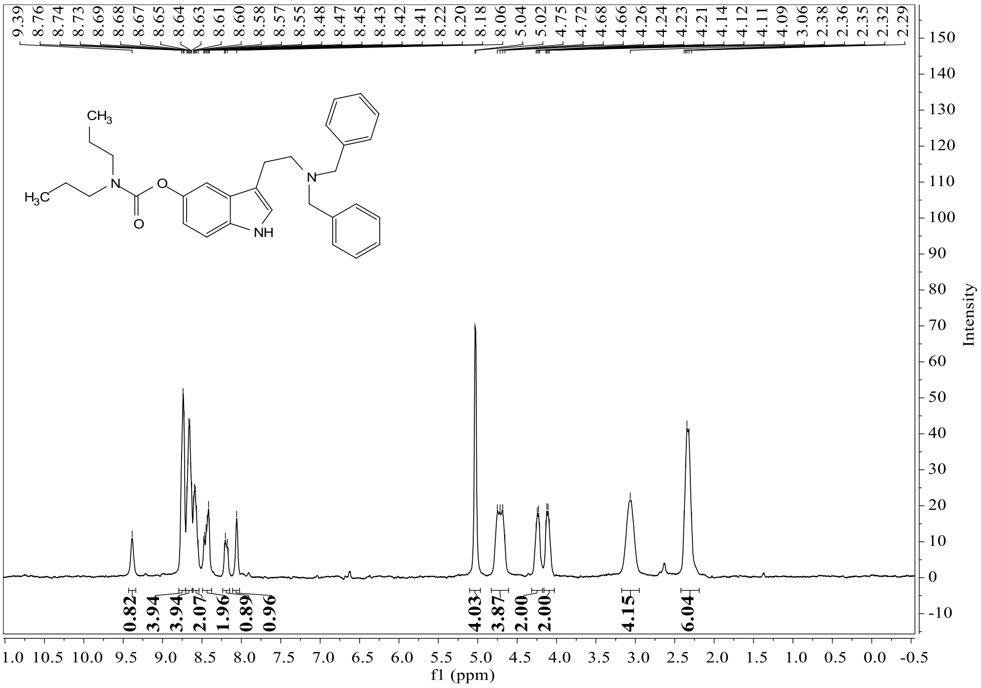

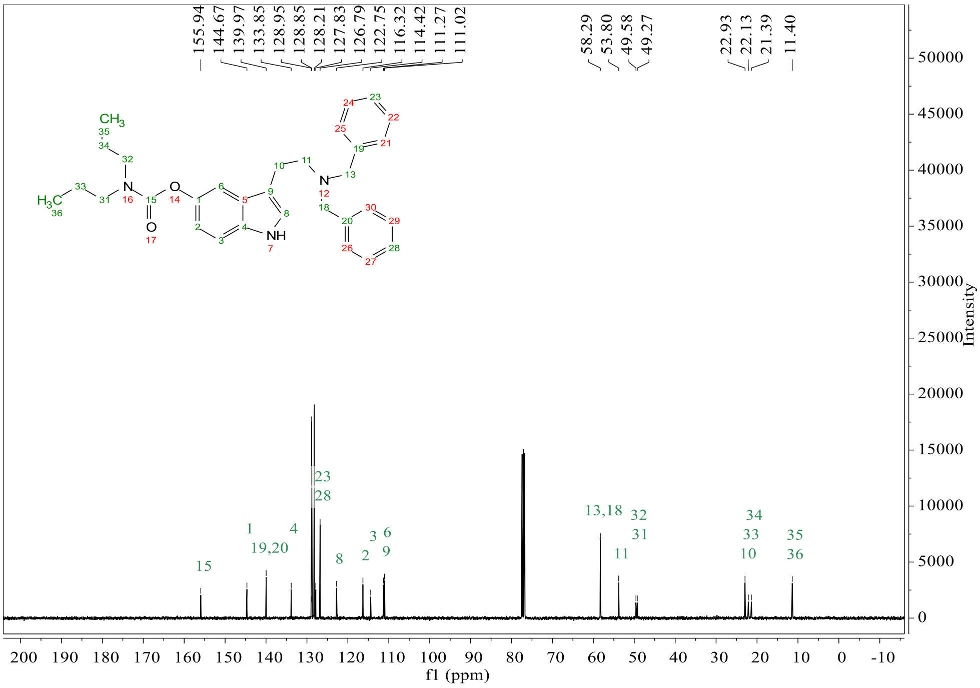

2m:

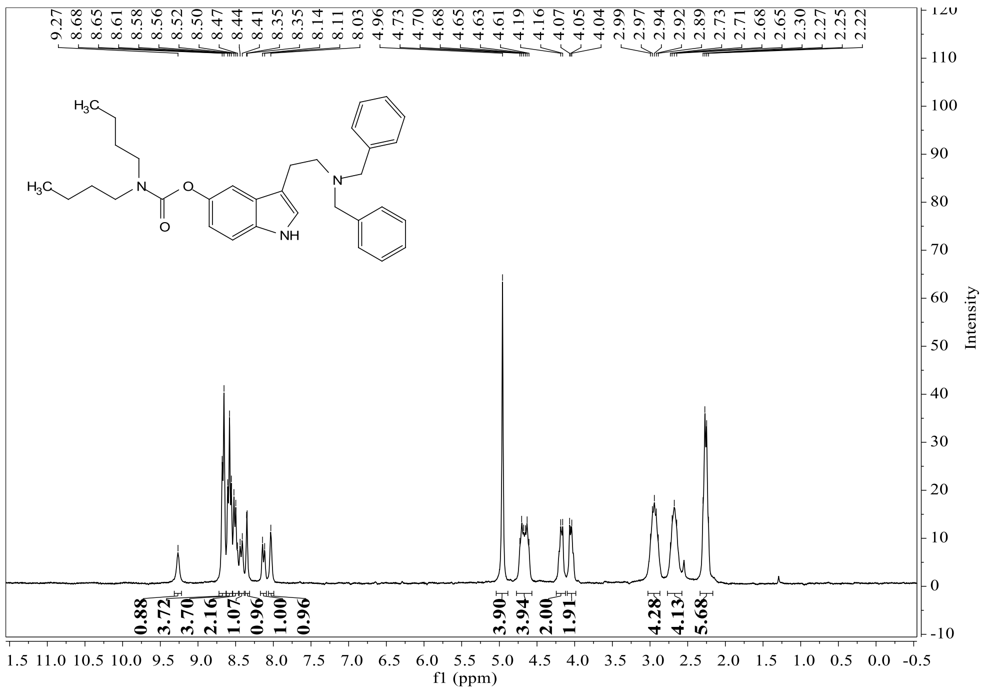

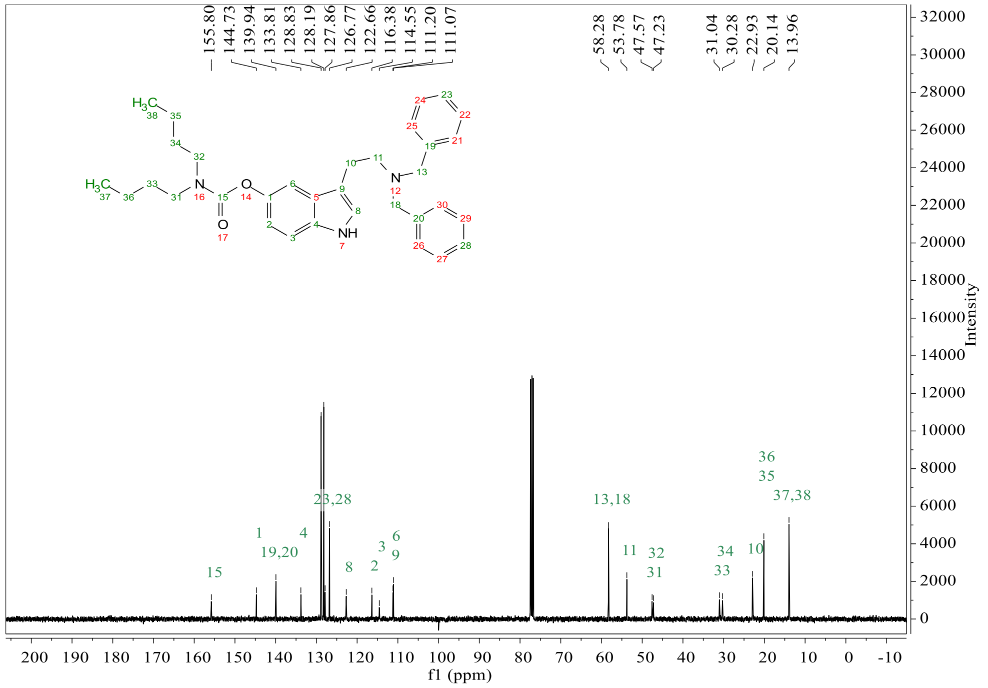

2n:

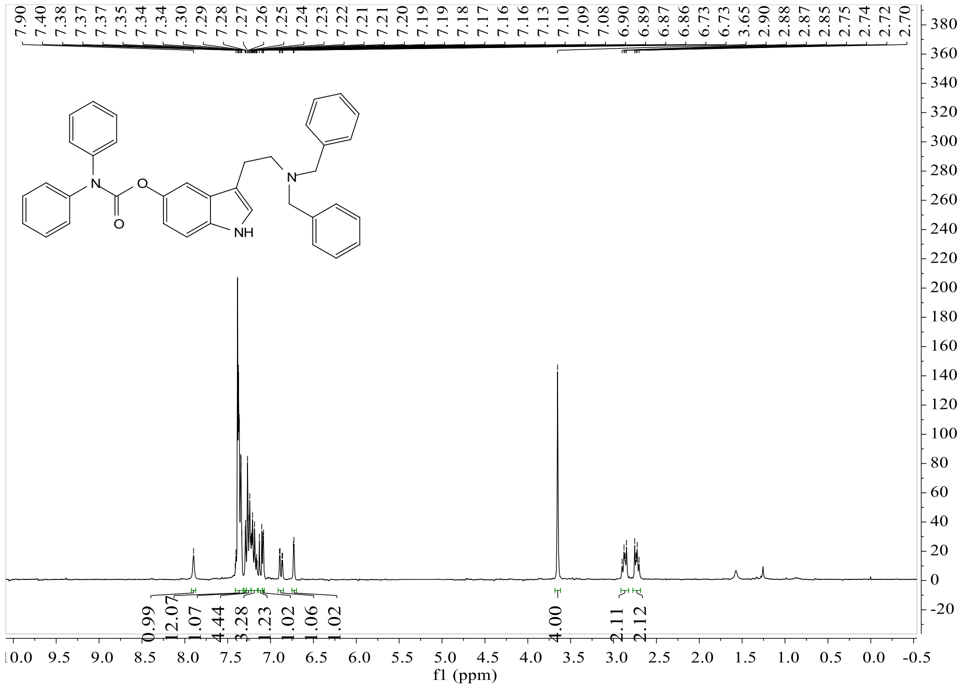

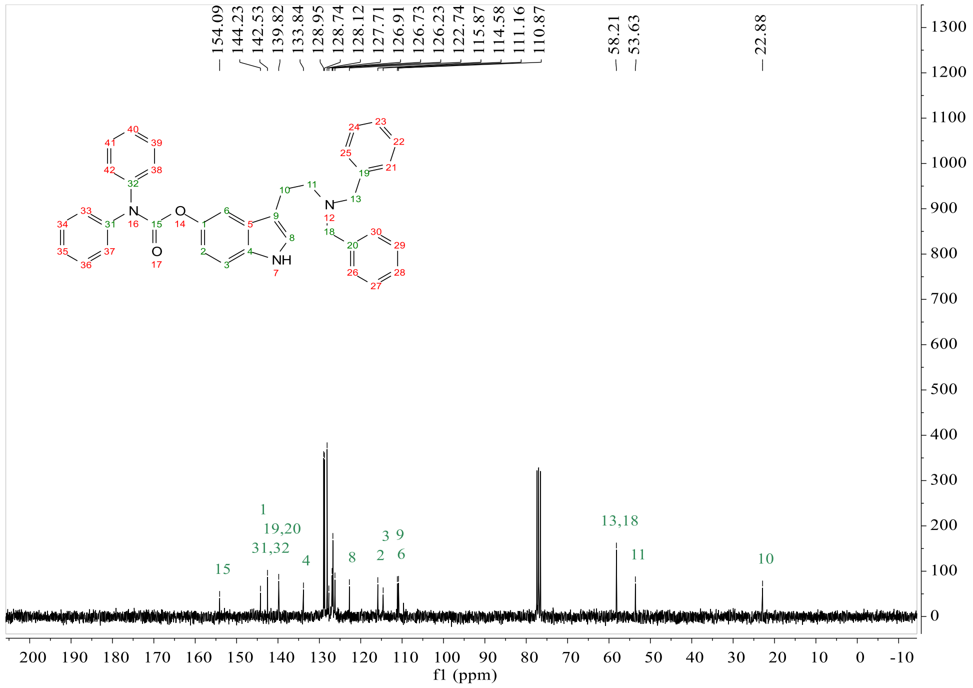

2o:

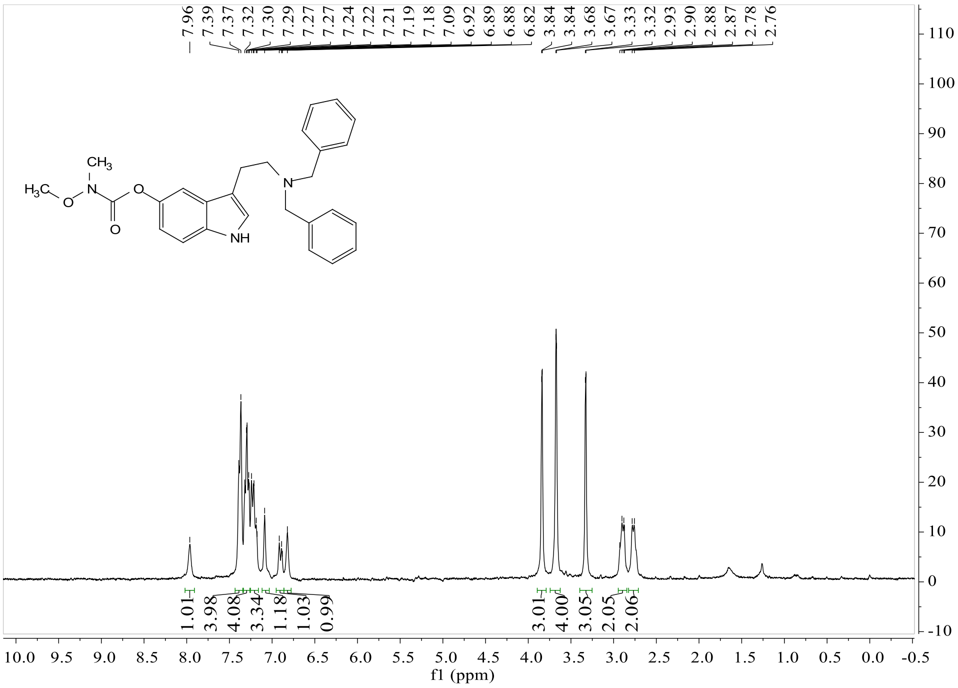

7.96
7.39
7.37
7.32
7.30
7.29
7.27
7.27
7.24
7.22
7.21
7.19
7.18
7.09
6.92
6.89
6.88
6.82
3.84
3.84
3.68
3.67
3.33
3.32
2.93
2.90
2.88
2.87
2.78
2.76
H_3C
O
N
CH_3
O
O
NH
N
1.01
3.98
4.08
3.34
1.18
1.03
0.99
3.01
4.00
3.05
2.05
2.06
f1 (ppm)

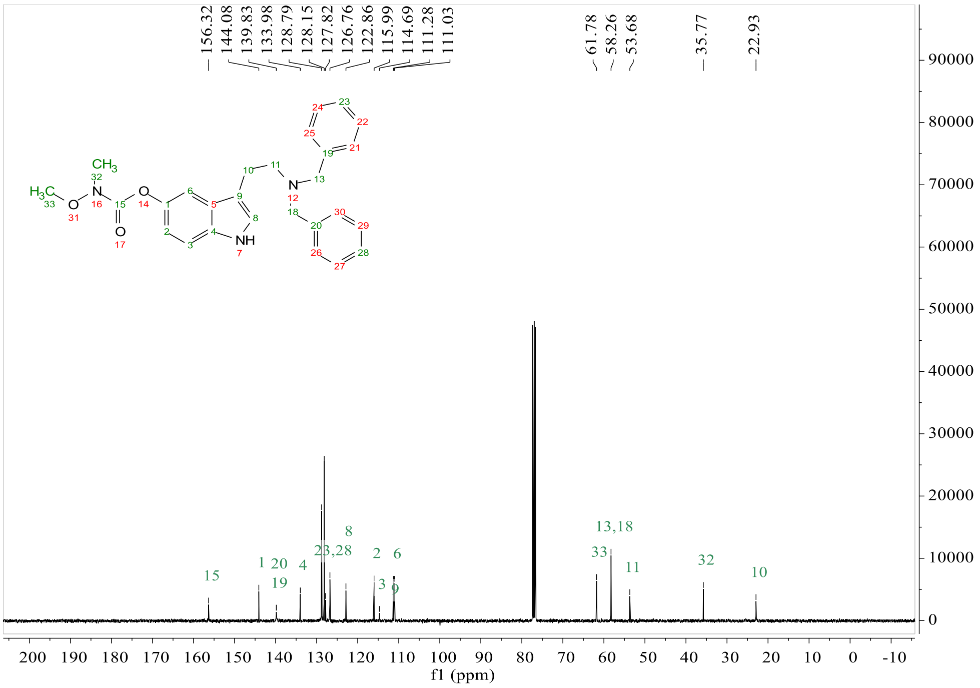

156.32
144.08
139.83
133.98
128.79
128.15
127.82
126.76
122.86
115.99
114.69
111.28
111.03
61.78
58.26
53.68
35.77
22.93
15
1 20
19
4
8
23,28
2
3
6
9
13,18
33
11
32
10
f1 (ppm)

2p:

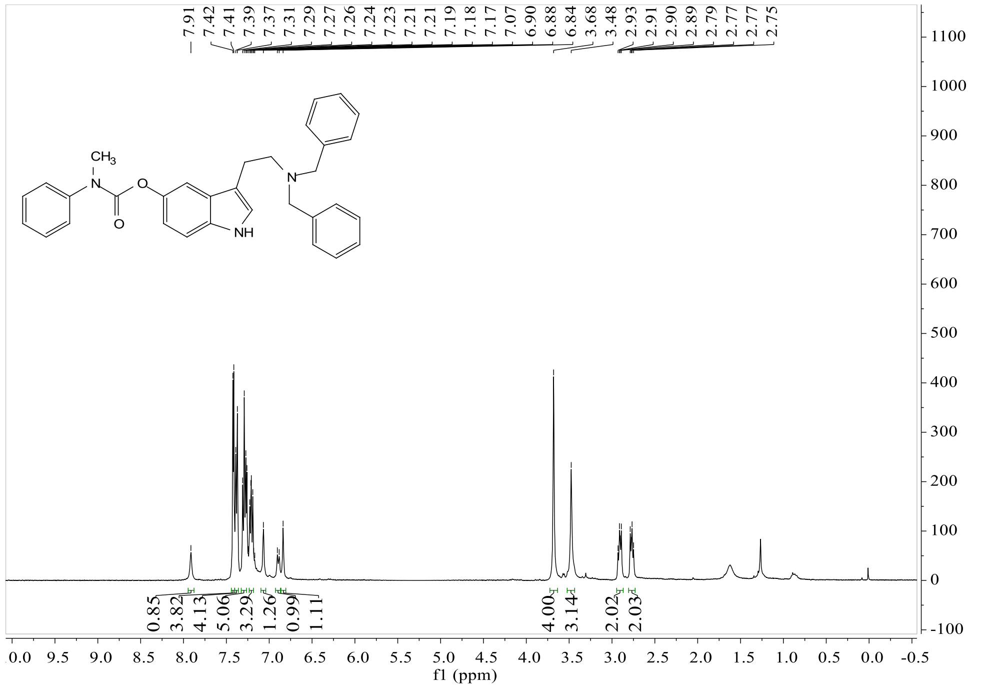
7.91
7.42
7.41
7.39
7.37
7.31
7.29
7.27
7.26
7.24
7.23
7.21
7.21
7.19
7.18
7.17
7.07
6.90
6.88
6.84
3.68
3.48
2.93
2.91
2.90
2.89
2.79
2.77
2.77
2.75
CH3
N
O
O
NH
N
0.85
3.82
4.13
5.06
3.29
1.26
0.99
1.11
4.00
3.14
2.02
2.03
f1 (ppm)

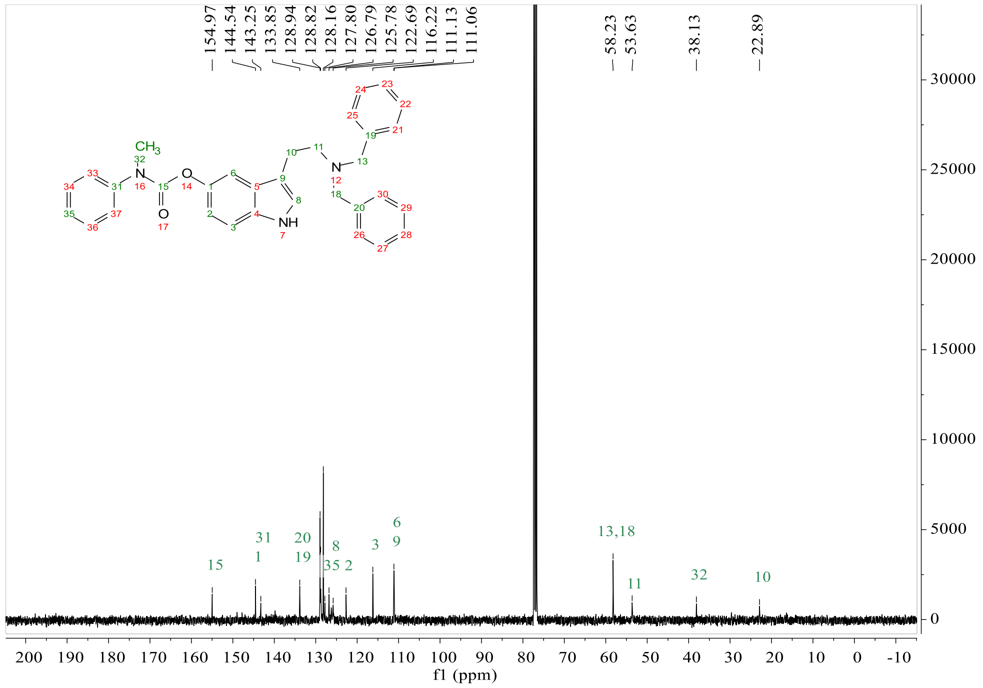
154.97
144.54
143.25
133.85
128.94
128.82
128.16
127.80
126.79
125.78
122.69
116.22
111.13
111.06
58.23
53.63
38.13
22.89
15
31
1
20
19
8
35
2
3
6
9
13,18
11
32
10
f1 (ppm)

2q:

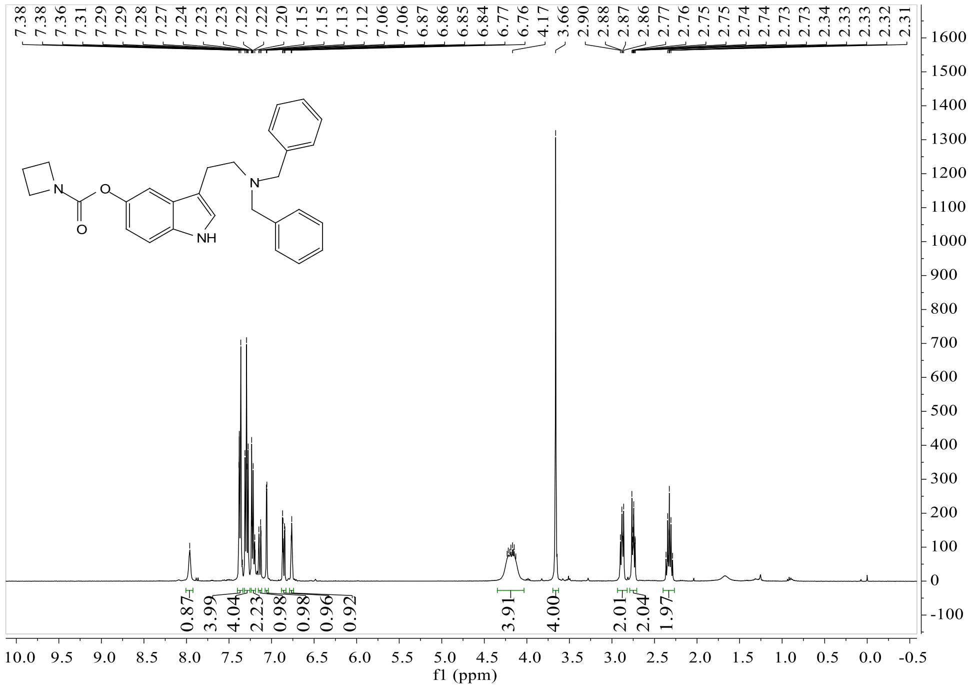

7.38 7.38 7.36 7.31 7.29 7.29 7.28 7.27 7.24 7.23 7.23 7.22 7.22 7.20 7.15 7.15 7.13 7.12 7.06 7.06 6.87 6.86 6.85 6.84 6.77 6.76 4.17 3.66 2.90 2.88 2.87 2.86 2.77 2.76 2.75 2.75 2.74 2.74 2.73 2.73 2.34 2.33 2.33 2.32 2.31
N
O
O
NH
N
0.87 3.99 4.04 2.23 0.98 0.98 0.96 0.92 3.91 4.00 2.01 2.04 1.97
f1 (ppm)

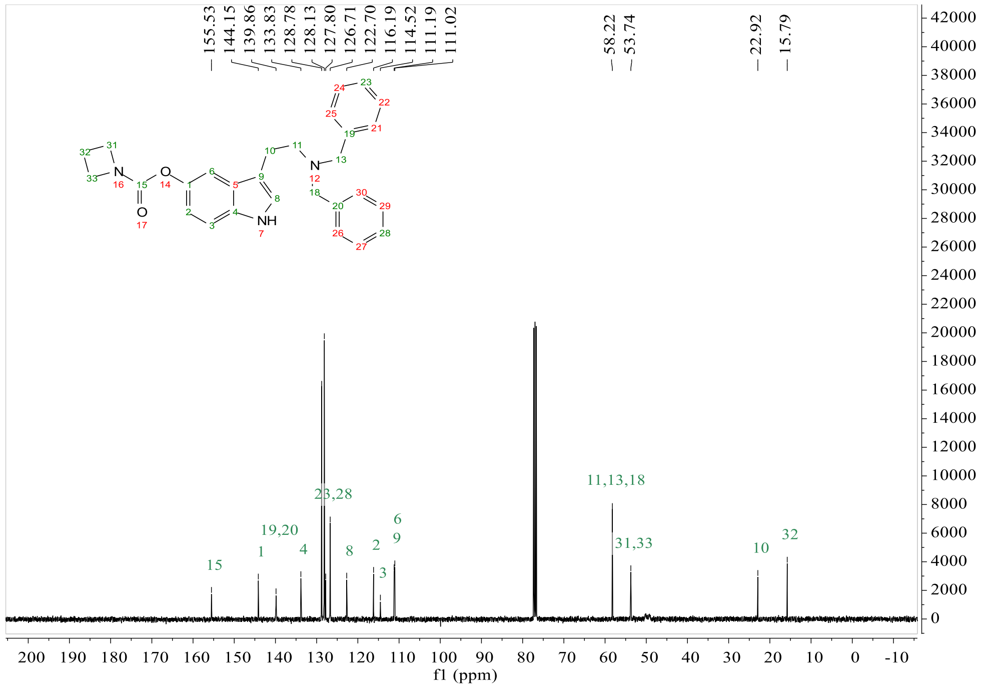

155.53 144.15 139.86 133.83 128.78 128.13 127.80 126.71 122.70 116.19 114.52 111.19 111.02 58.22 53.74 22.92 15.79
15 1 19,20 4 23,28 8 2 3 6 9 11,13,18 31,33 10 32
f1 (ppm)

2r:

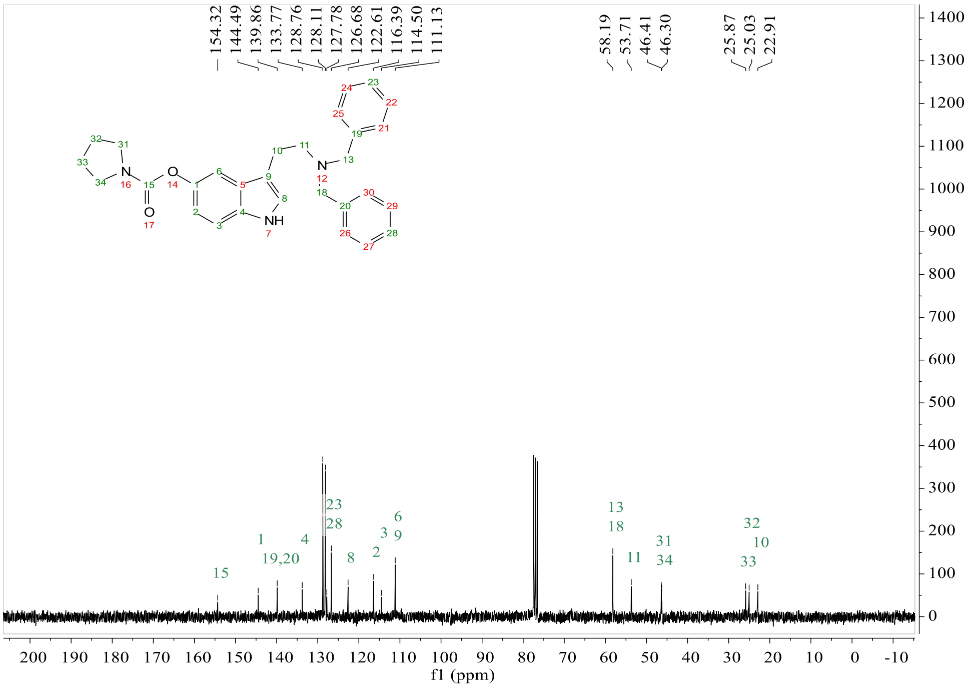
154.32
144.49
139.86
133.77
128.76
128.11
127.78
126.68
122.61
116.39
114.50
111.13
58.19
53.71
46.41
46.30
25.87
25.03
22.91
f1 (ppm)

2s:

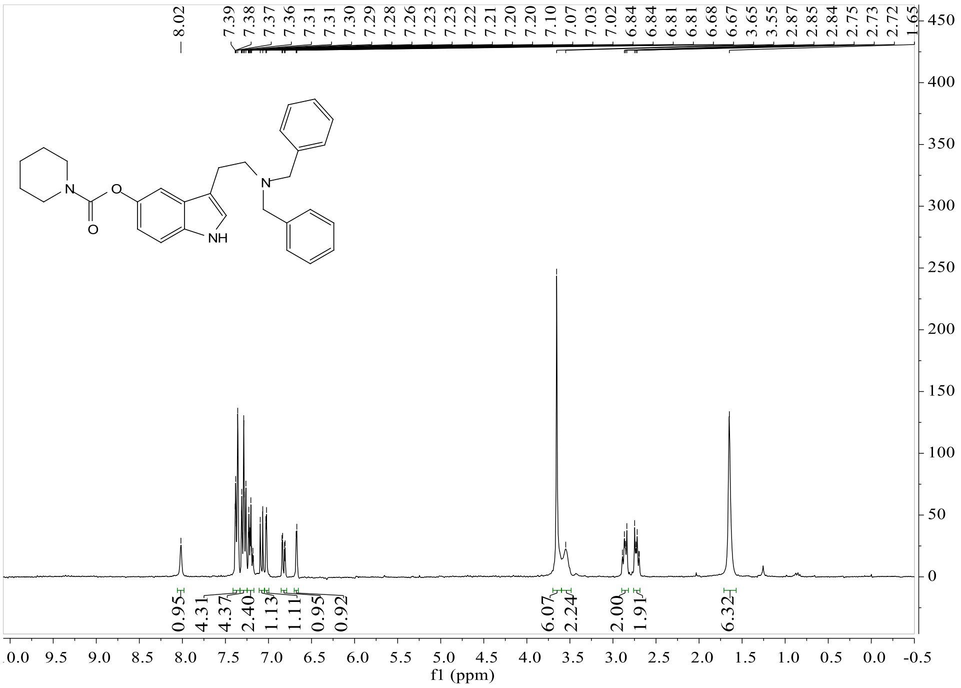

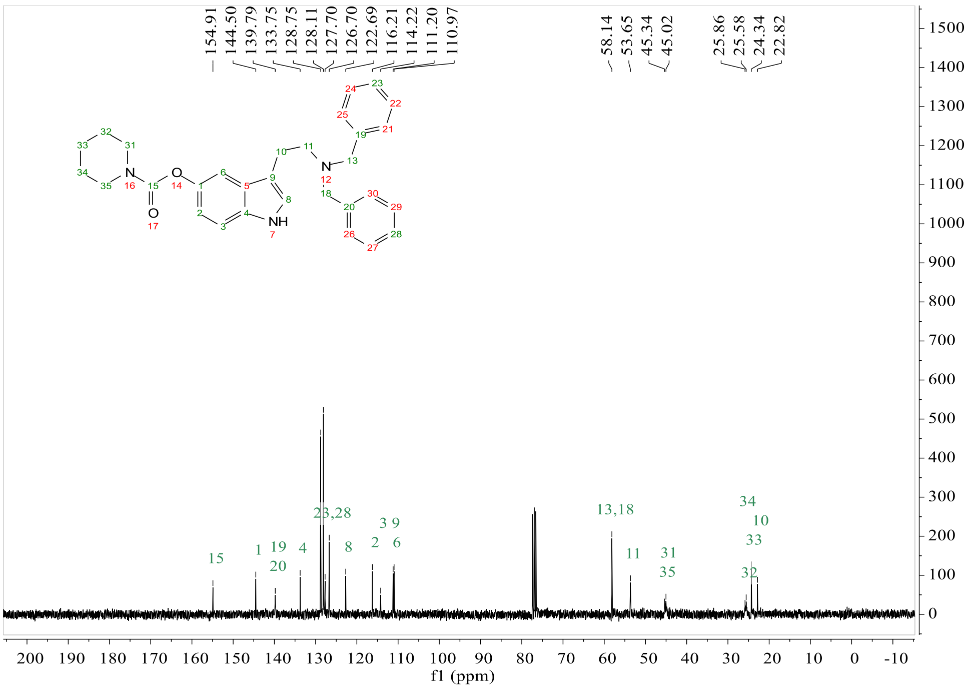

2t:

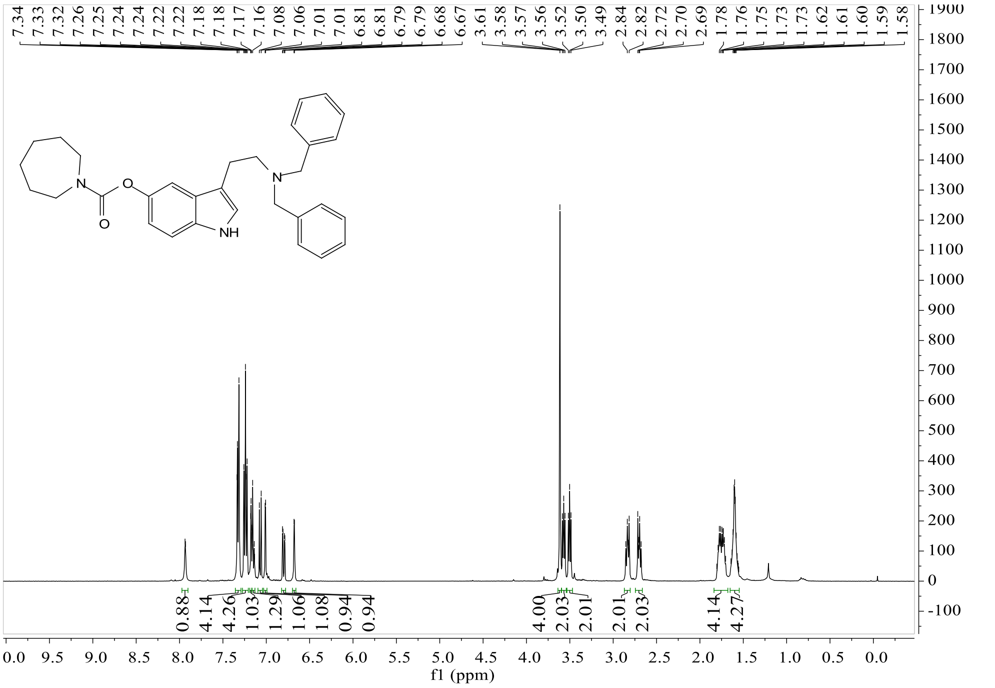

7.34 7.33 7.32 7.26 7.25 7.24 7.24 7.22 7.22 7.18 7.18 7.17 7.16 7.08 7.06 7.01 7.01 6.81 6.81 6.79 6.79 6.68 6.67 3.61 3.58 3.57 3.56 3.52 3.50 3.49 2.84 2.82 2.72 2.70 2.69 1.78 1.76 1.75 1.73 1.73 1.62 1.61 1.60 1.59 1.58
NH
N
O
0.88 4.14 4.26 1.03 1.29 1.06 1.08 0.94 0.94 4.00 2.03 2.01 2.01 2.03 4.14 4.27
f1 (ppm)

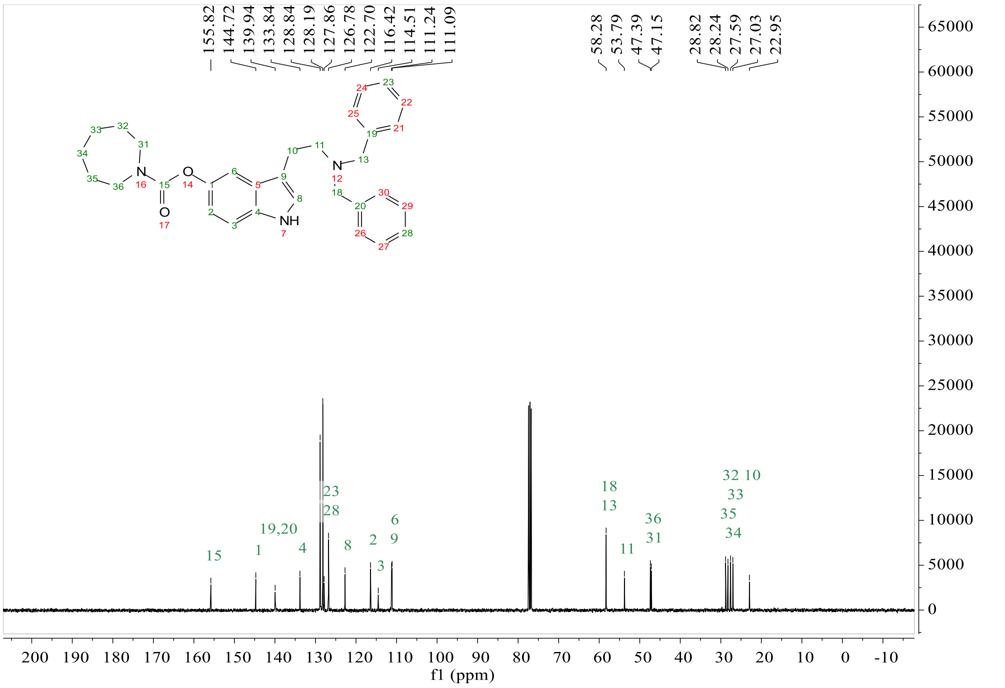

155.82 144.72 139.94 133.84 128.84 128.19 127.86 126.78 122.70 116.42 114.51 111.24 111.09
58.28 53.79 47.39 47.15
28.82 28.24 27.59 27.03 22.95
15 1 19,20 4 23 28 8 2 3 6 9 18 13 11 36 31 32 10 33 35 34
f1 (ppm)

2u:

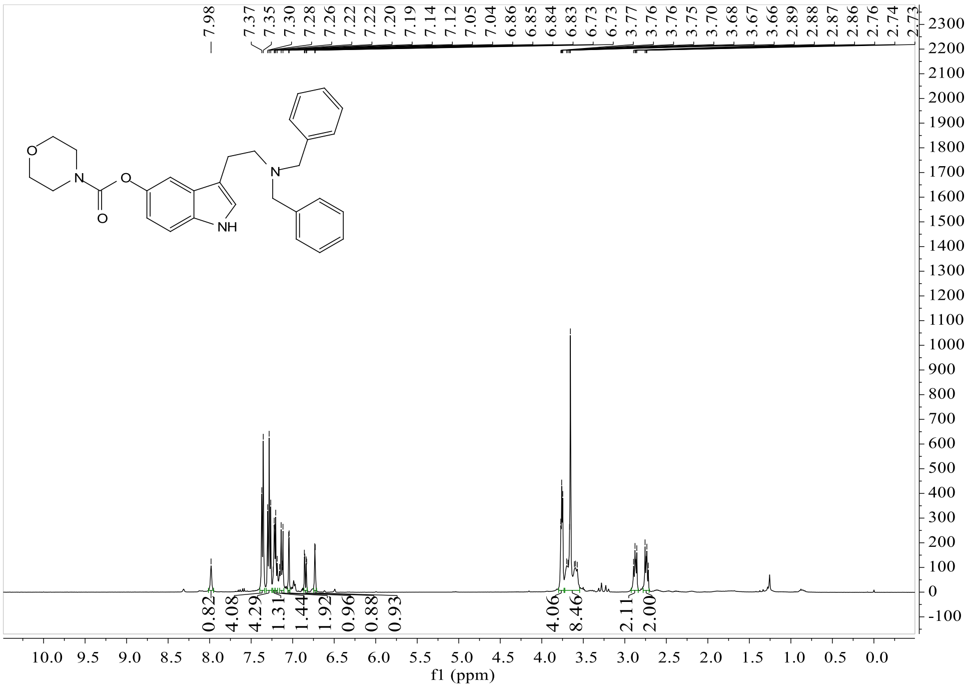

7.98
7.37
7.35
7.30
7.28
7.26
7.22
7.22
7.20
7.19
7.14
7.12
7.05
7.04
6.86
6.85
6.84
6.83
6.73
6.73
6.73
3.77
3.76
3.76
3.75
3.70
3.68
3.67
3.66
2.89
2.88
2.87
2.86
2.76
2.74
2.73
NH
0.82
4.08
4.29
1.31
1.44
1.92
0.96
0.88
0.93
4.06
8.46
2.11
2.00
f1 (ppm)

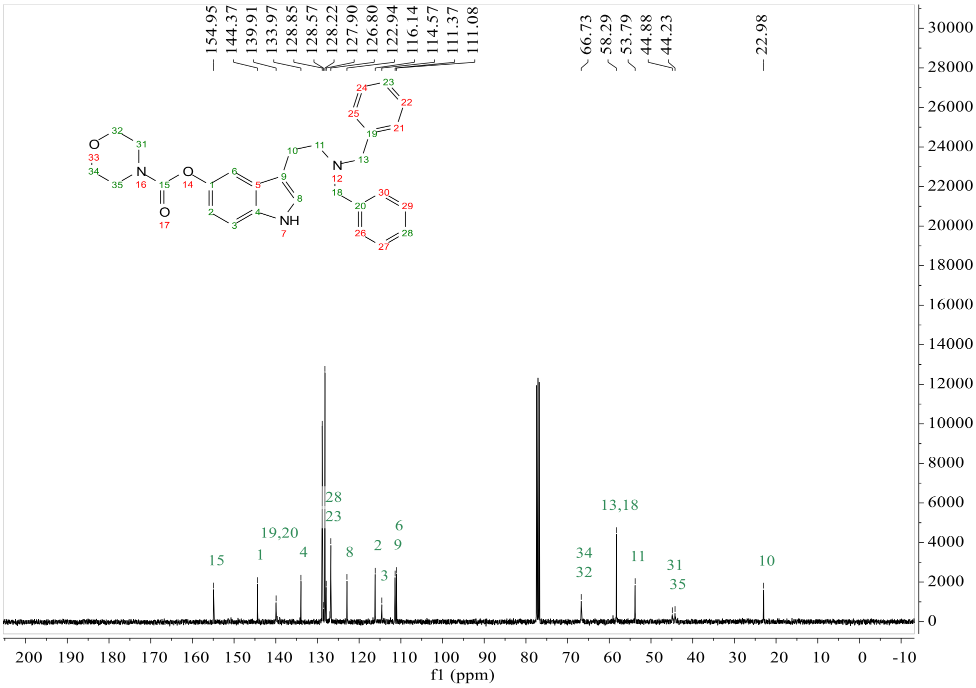

154.95
144.37
139.91
133.97
128.85
128.57
128.22
127.90
126.80
122.94
116.14
114.57
111.37
111.08
66.73
58.29
53.79
44.88
44.23
22.98
15
1
19,20
4
28
23
8
2
3
6
9
34
32
13,18
11
31
35
10
f1 (ppm)

3a:

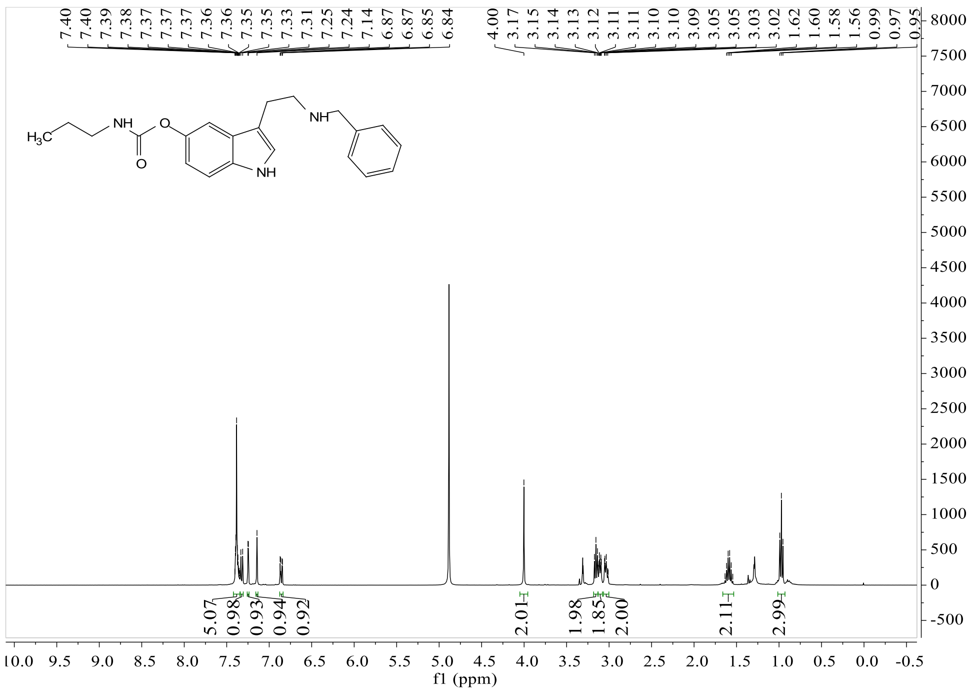

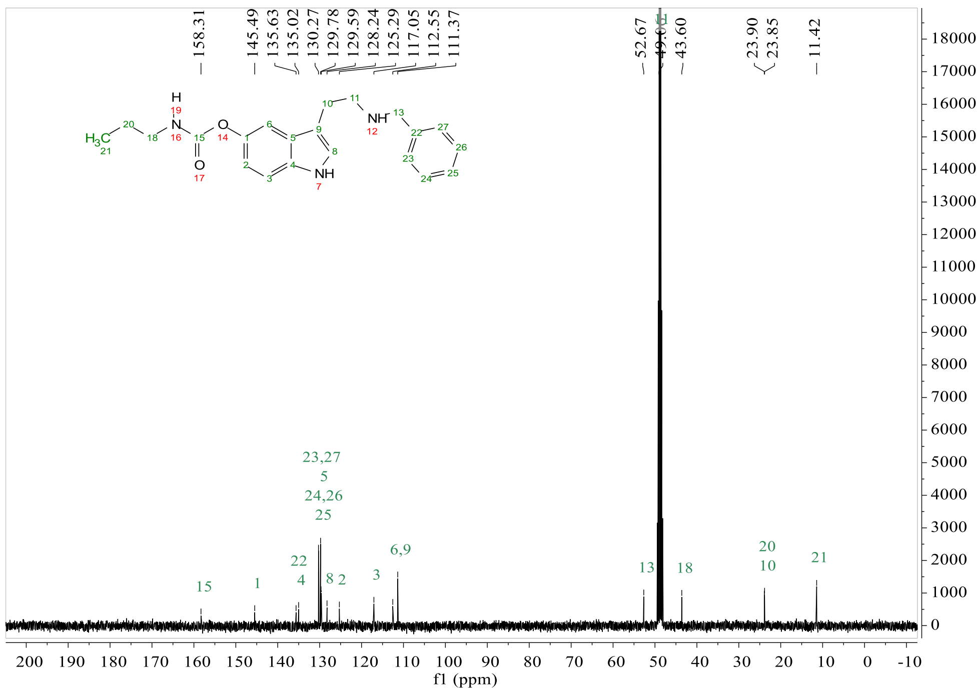

3b:

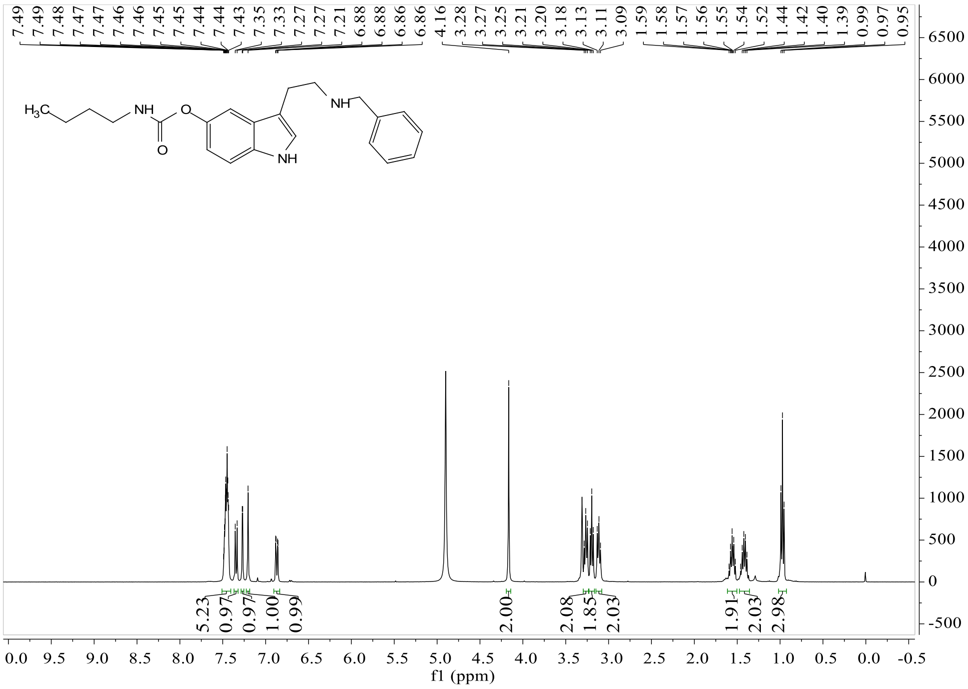

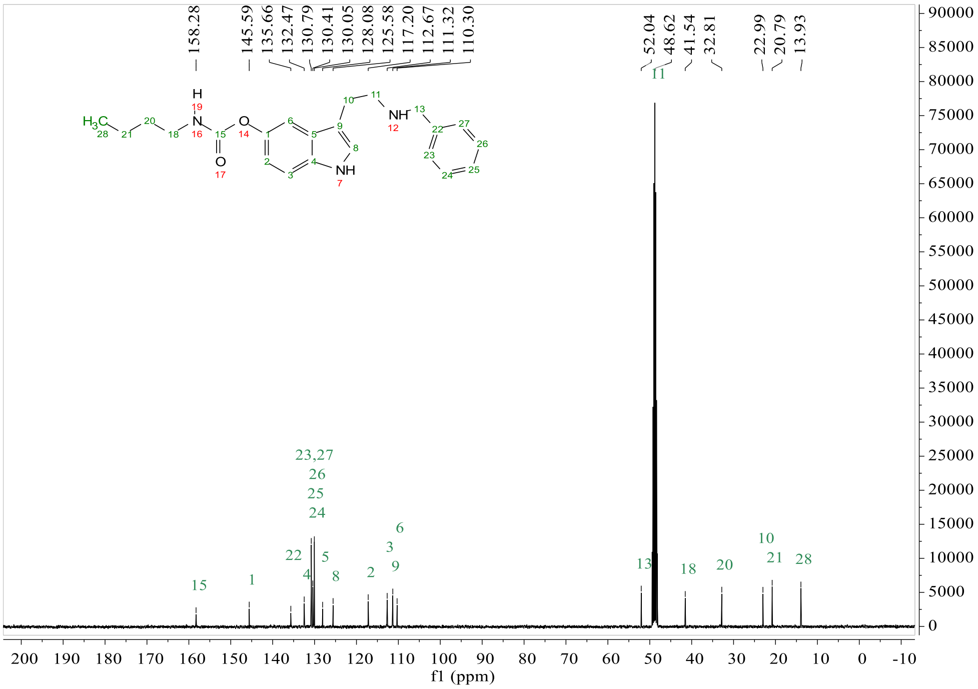

3c:

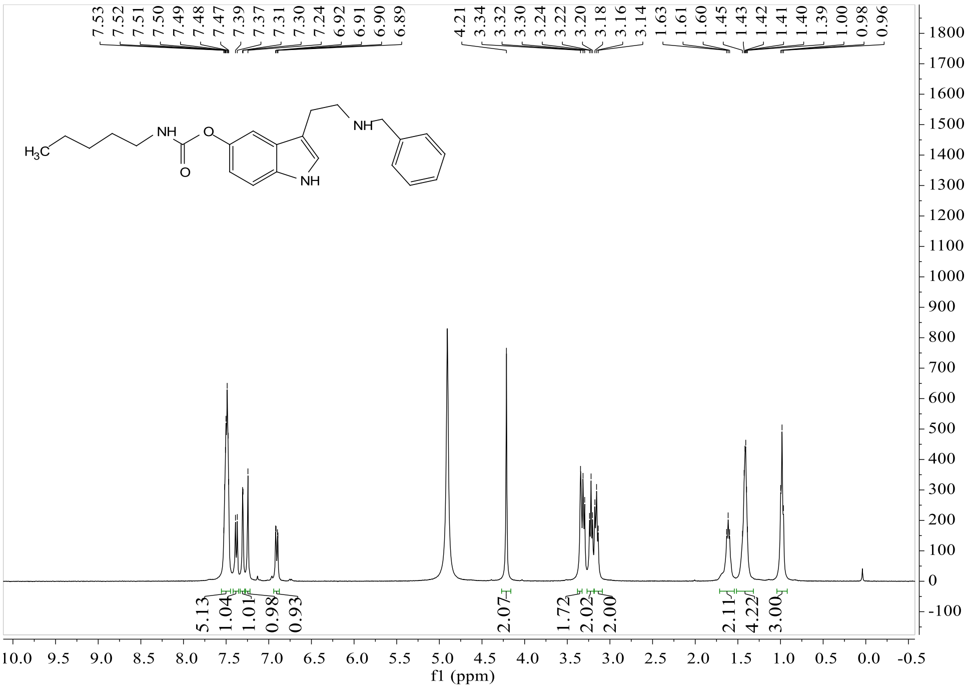
7.53
7.52
7.51
7.50
7.49
7.48
7.47
7.39
7.37
7.31
7.30
7.24
6.92
6.91
6.90
6.89
4.21
3.34
3.32
3.30
3.24
3.22
3.20
3.18
3.16
3.14
1.63
1.61
1.60
1.45
1.43
1.42
1.41
1.40
1.39
1.00
0.98
0.96
5.13
1.04
1.01
0.98
0.93
2.07
1.72
2.02
2.00
2.11
4.22
3.00
f1 (ppm)

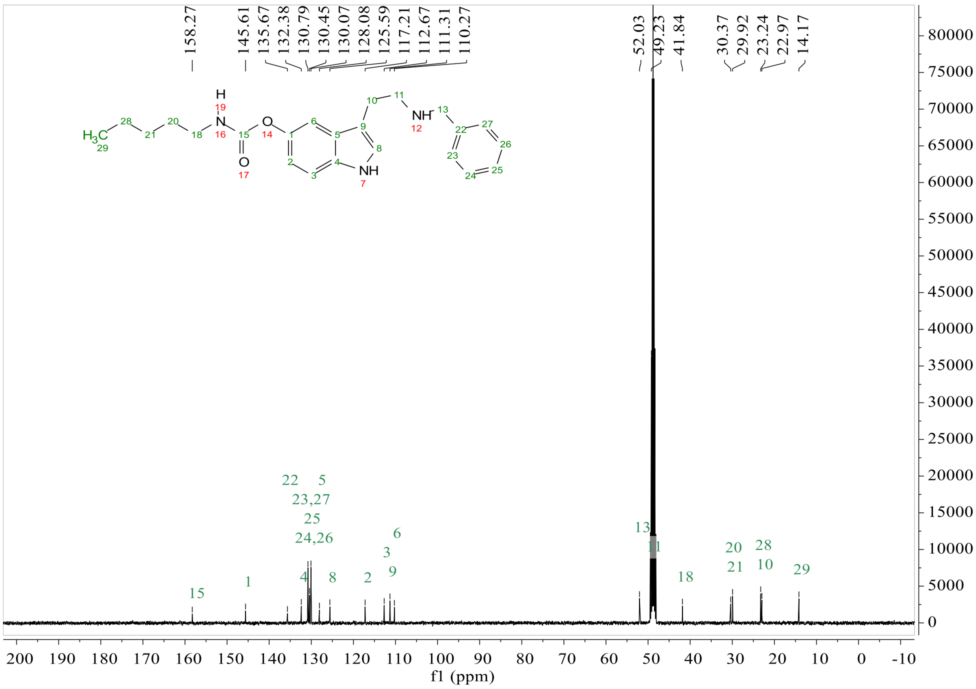
158.27
145.61
135.67
132.38
130.79
130.45
130.07
128.08
125.59
117.21
112.67
111.31
110.27
52.03
49.23
41.84
30.37
29.92
23.24
22.97
14.17
f1 (ppm)

3d:

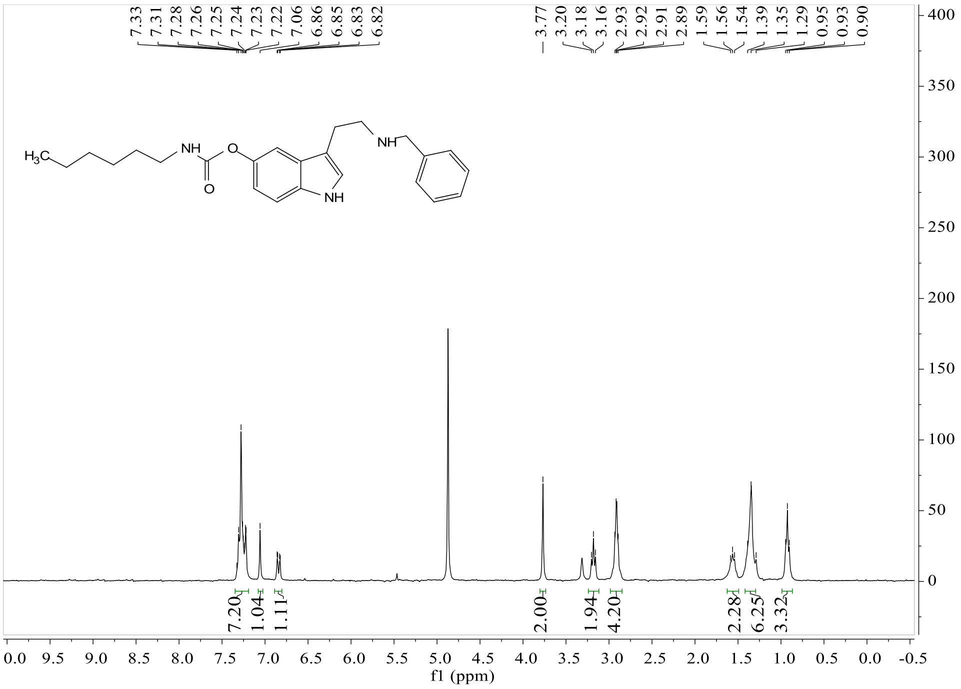

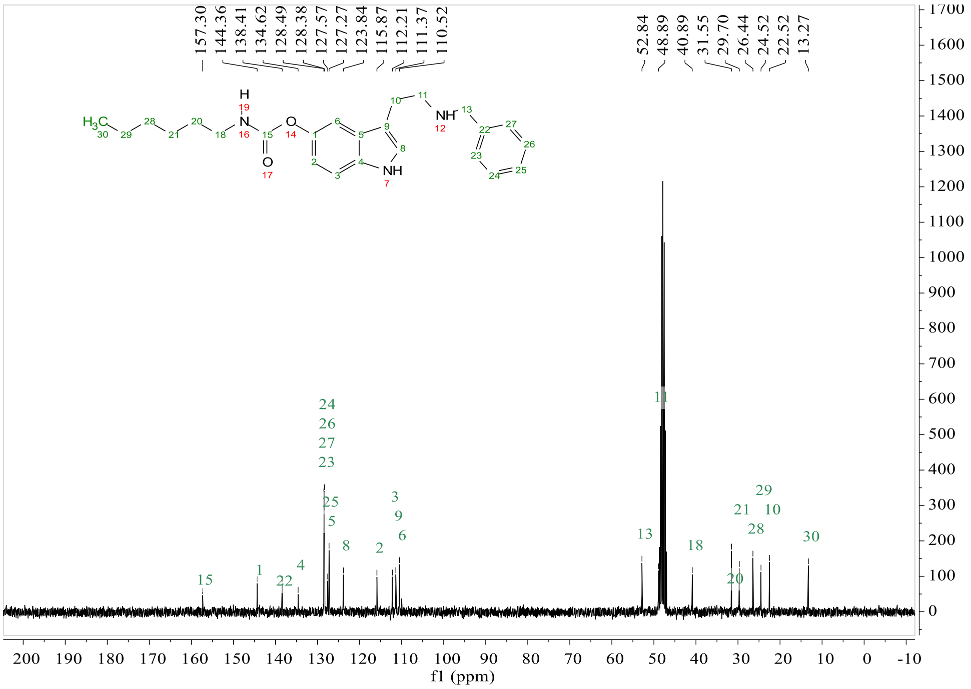

3e:

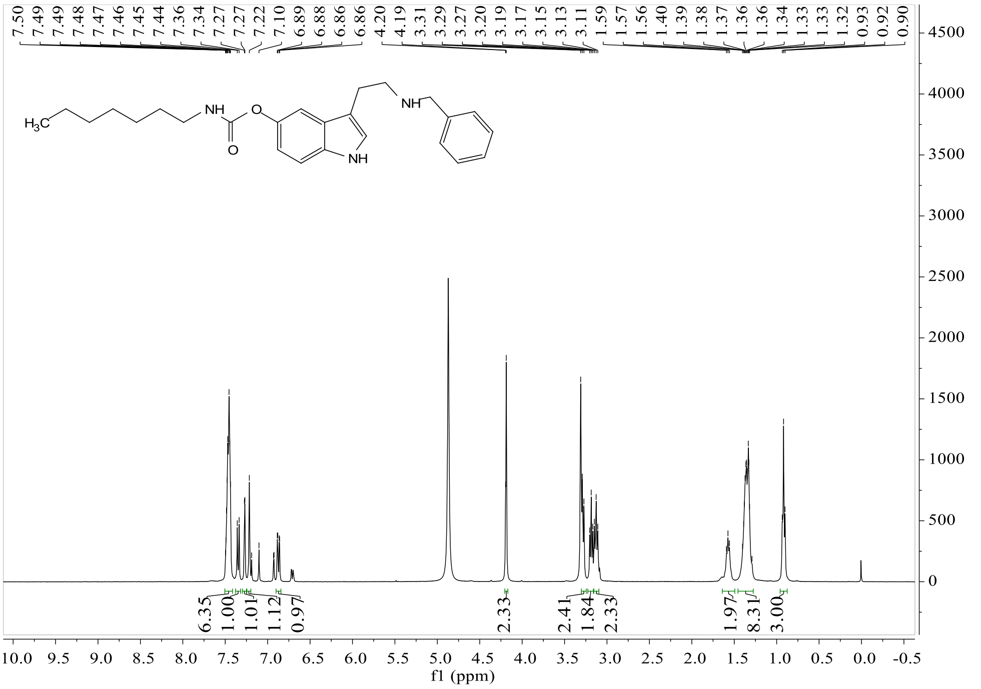

7.50
7.49
7.49
7.48
7.47
7.46
7.45
7.44
7.36
7.34
7.27
7.27
7.22
7.10
6.89
6.88
6.86
6.86
4.20
4.19
3.31
3.29
3.27
3.20
3.19
3.17
3.15
3.13
3.11
1.59
1.57
1.56
1.40
1.39
1.38
1.37
1.36
1.36
1.34
1.33
1.33
1.32
0.93
0.92
0.90
NH
O
NH
NH
O
6.35
1.00
1.01
1.12
0.97
2.33
2.41
1.84
2.33
1.97
8.31
3.00
f1 (ppm)

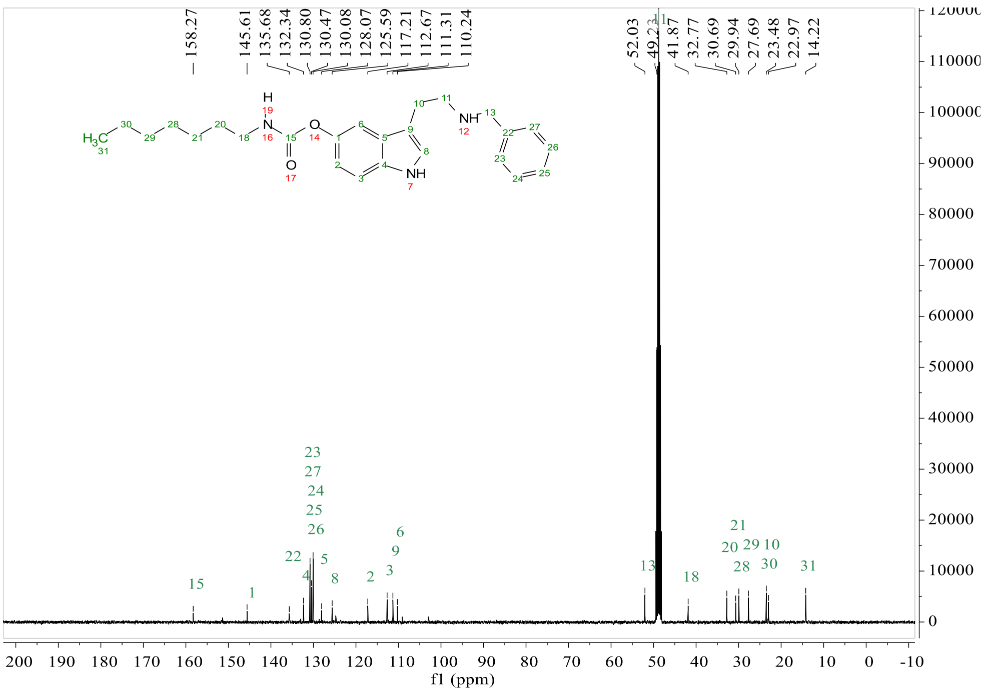

158.27
145.61
135.68
132.34
130.80
130.47
130.08
128.07
125.59
117.21
112.67
111.31
110.24
52.03
49.23
41.87
32.77
30.69
29.94
27.69
23.48
22.97
14.22
f1 (ppm)

3f:

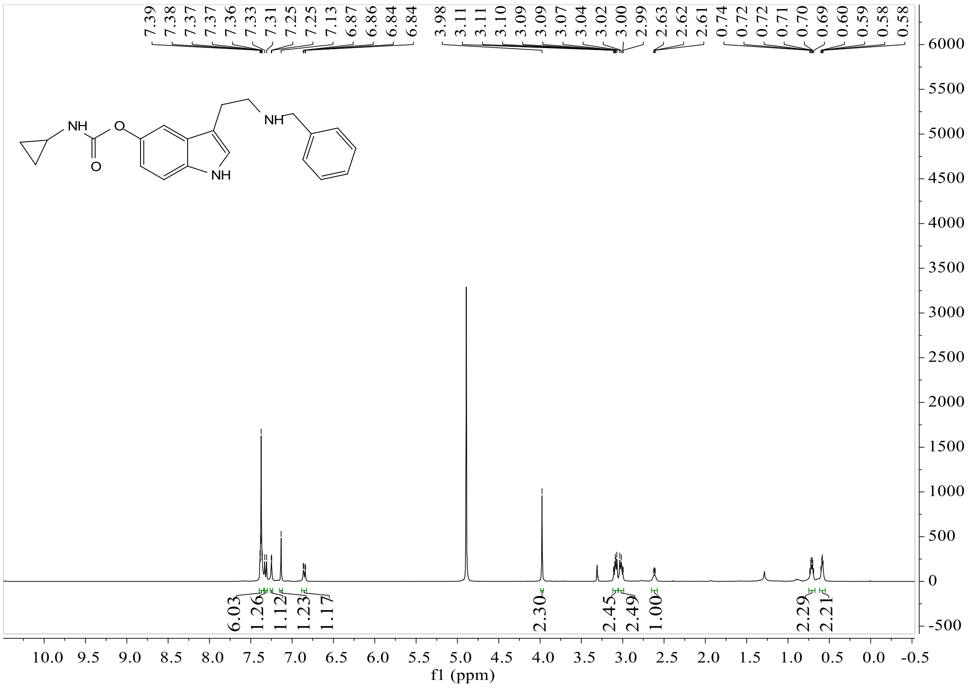

7.39 7.38 7.37 7.37 7.36 7.33 7.31 7.25 7.25 7.13 6.87 6.86 6.84 6.84 3.98 3.11 3.11 3.10 3.09 3.09 3.07 3.04 3.02 3.00 2.99 2.63 2.62 2.61 0.74 0.72 0.72 0.71 0.70 0.69 0.60 0.59 0.58 0.58
6.03 1.26 1.12 1.23 1.17 2.30 2.45 2.49 1.00 2.29 2.21
f1 (ppm)

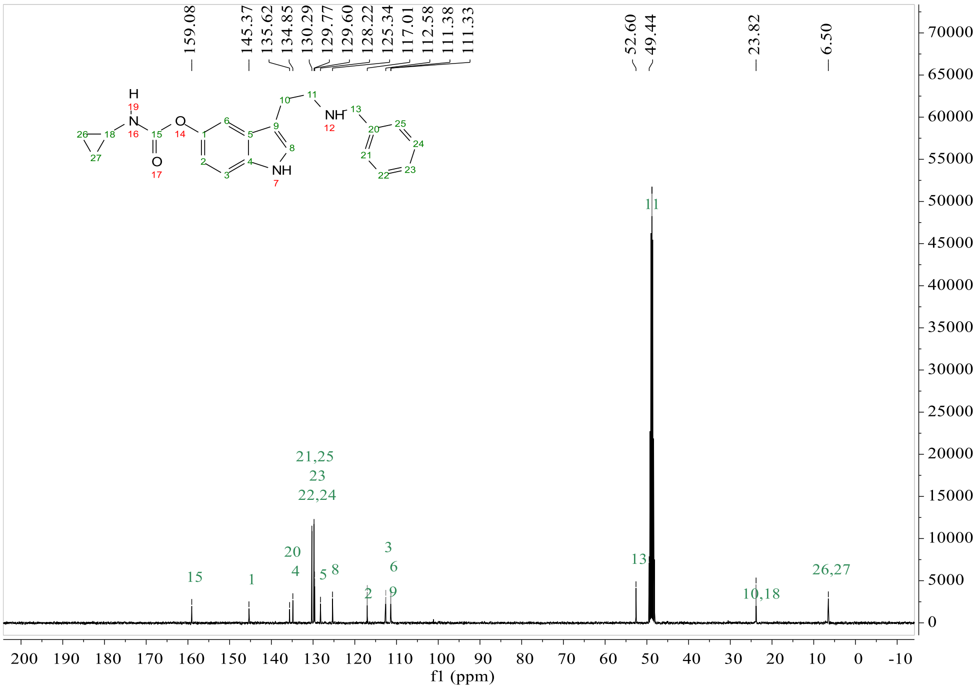

159.08 145.37 135.62 134.85 130.29 129.77 129.60 128.22 125.34 117.01 112.58 111.38 111.33 52.60 49.44 23.82 6.50
f1 (ppm)

3g:

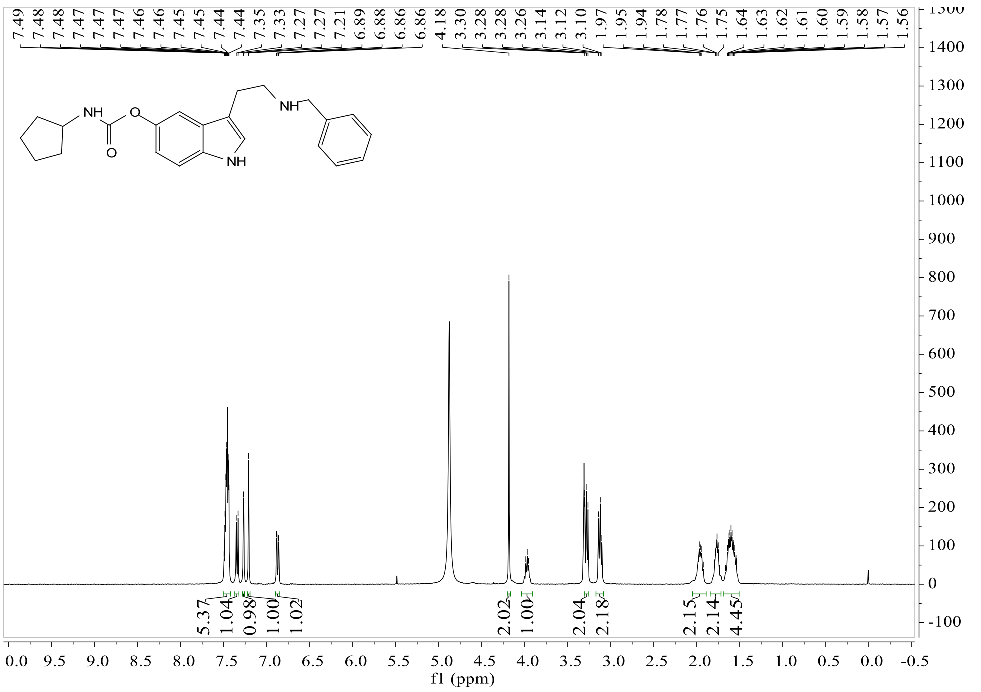

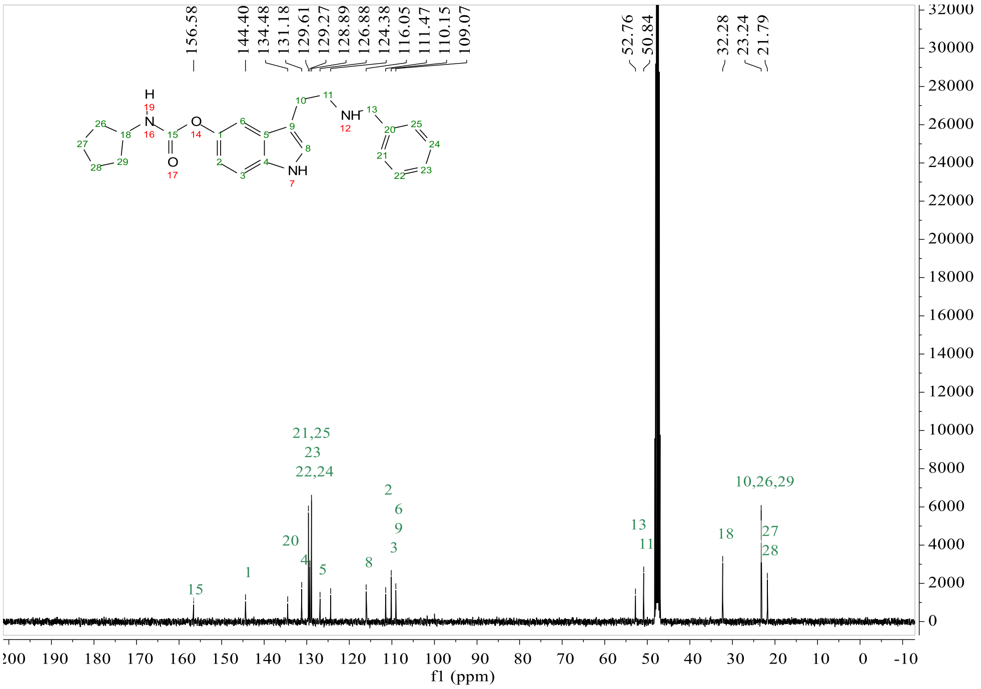

3h:

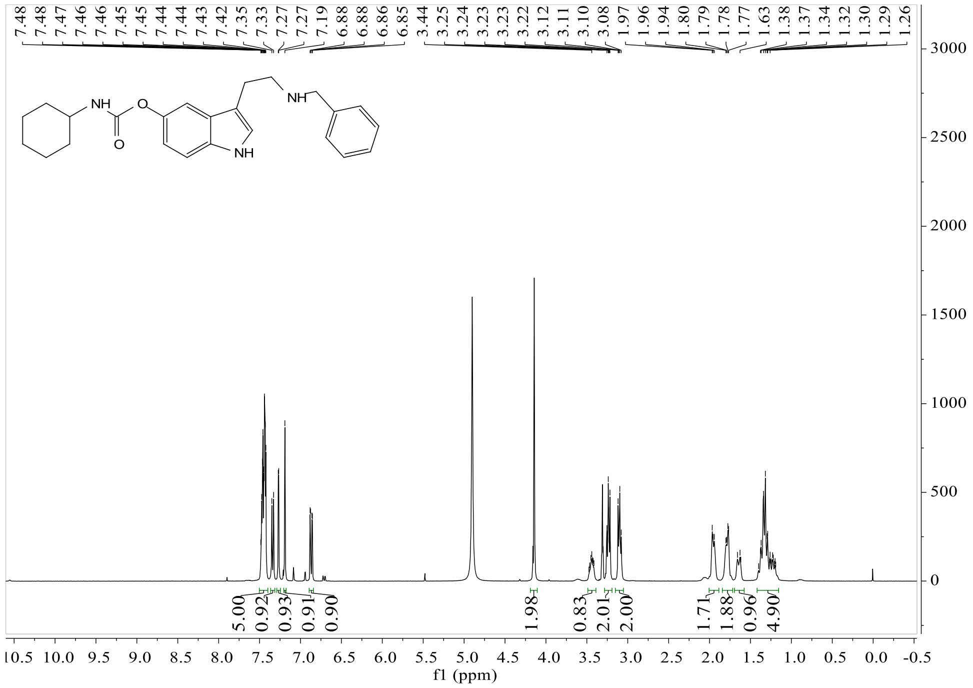

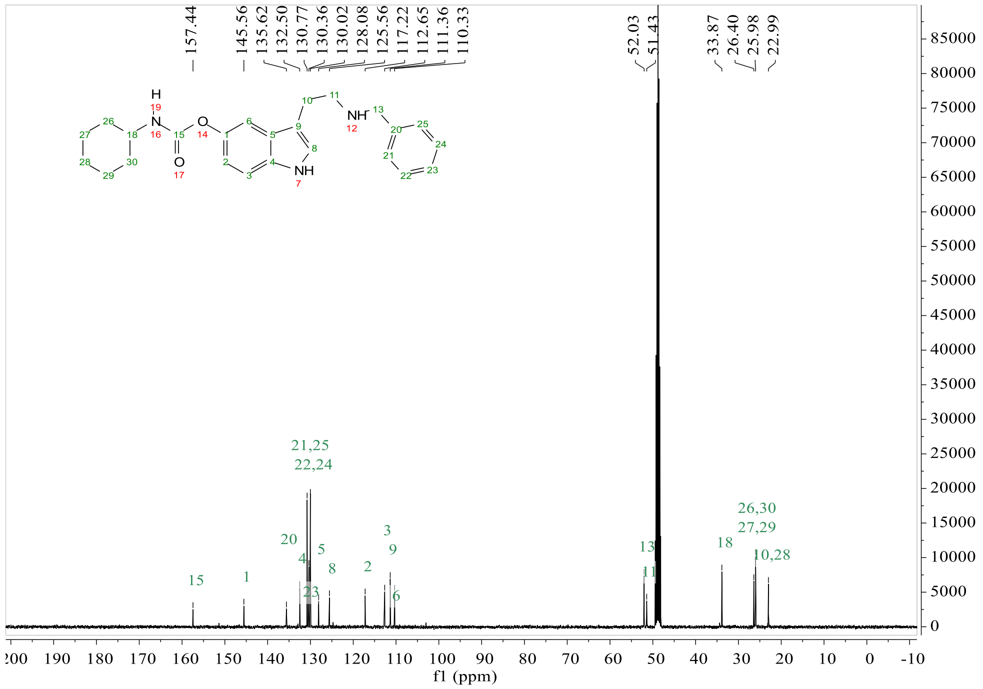

3i:

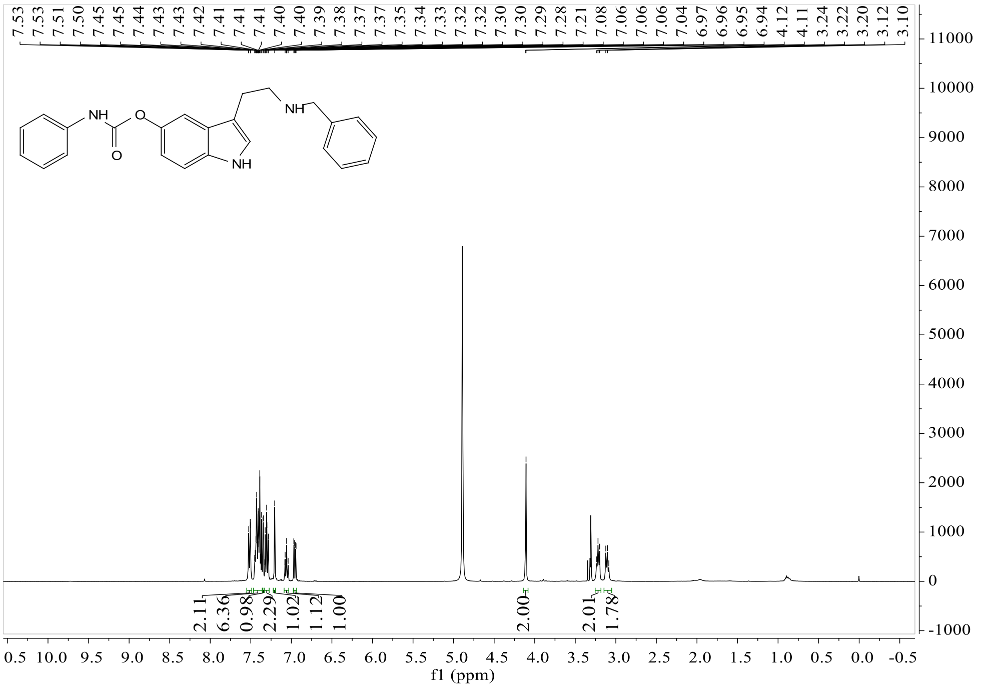

7.53
7.53
7.51
7.50
7.45
7.45
7.44
7.43
7.43
7.42
7.41
7.41
7.41
7.40
7.40
7.39
7.38
7.37
7.37
7.35
7.34
7.33
7.32
7.32
7.30
7.30
7.29
7.28
7.21
7.08
7.06
7.06
7.04
6.97
6.96
6.95
6.94
4.12
4.11
3.24
3.22
3.20
3.12
3.10
2.11
6.36
0.98
2.29
1.02
1.12
1.00
2.00
2.01
1.78
f1 (ppm)

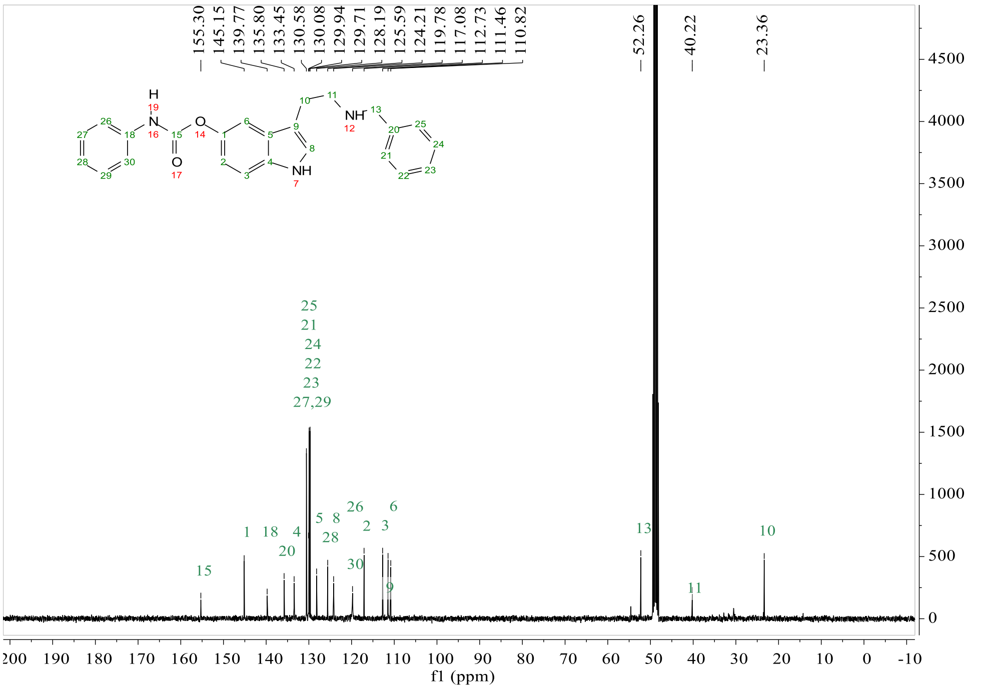

155.30
145.15
139.77
135.80
133.45
130.58
130.08
129.94
129.71
128.19
125.59
124.21
119.78
117.08
112.73
111.46
110.82
52.26
40.22
23.36
f1 (ppm)

3j:

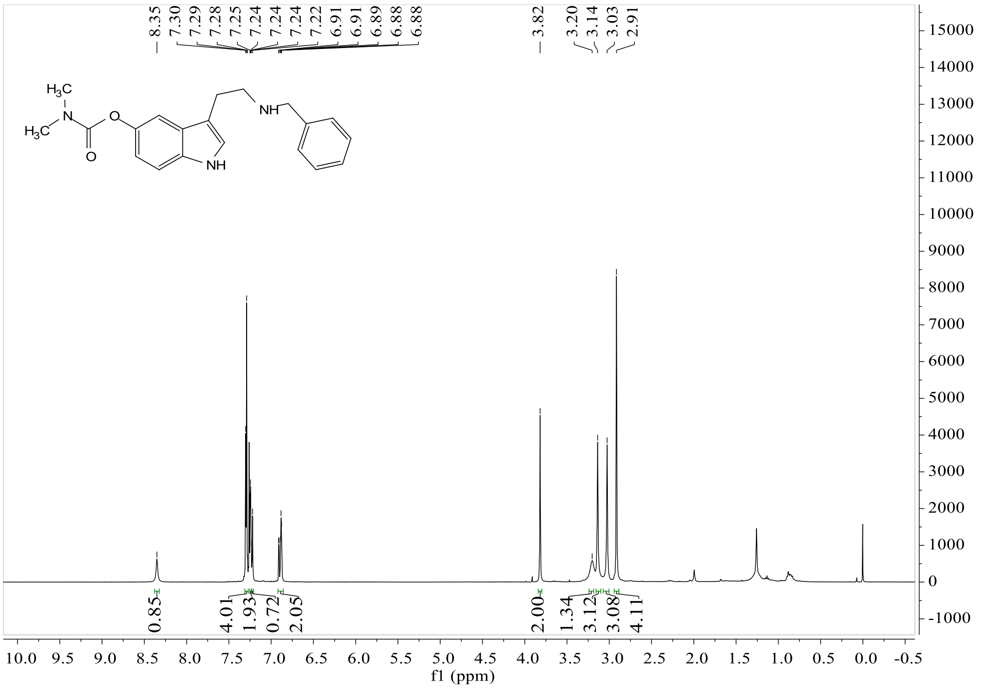

8.35
7.30
7.29
7.28
7.25
7.24
7.24
7.24
7.22
6.91
6.91
6.89
6.88
6.88
3.82
3.20
3.14
3.03
2.91
NH
0.85
4.01
1.93
0.72
2.05
2.00
1.34
3.12
3.08
4.11
f1 (ppm)

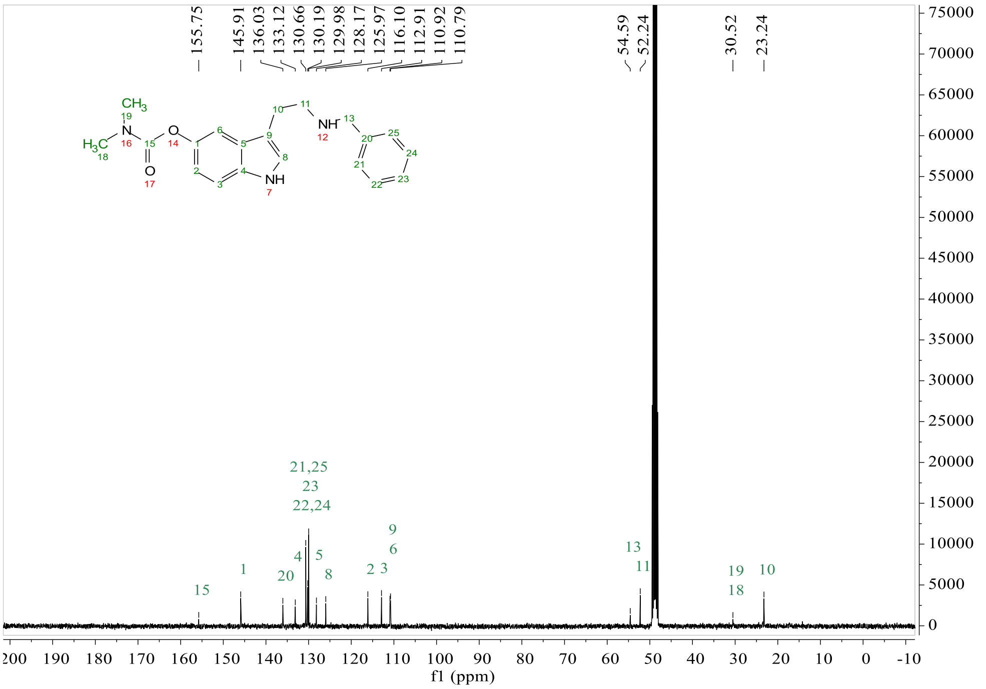

155.75
145.91
136.03
133.12
130.66
130.19
129.98
128.17
125.97
116.10
112.91
110.92
110.79
54.59
52.24
30.52
23.24
21,25
23
22,24
f1 (ppm)

3k:

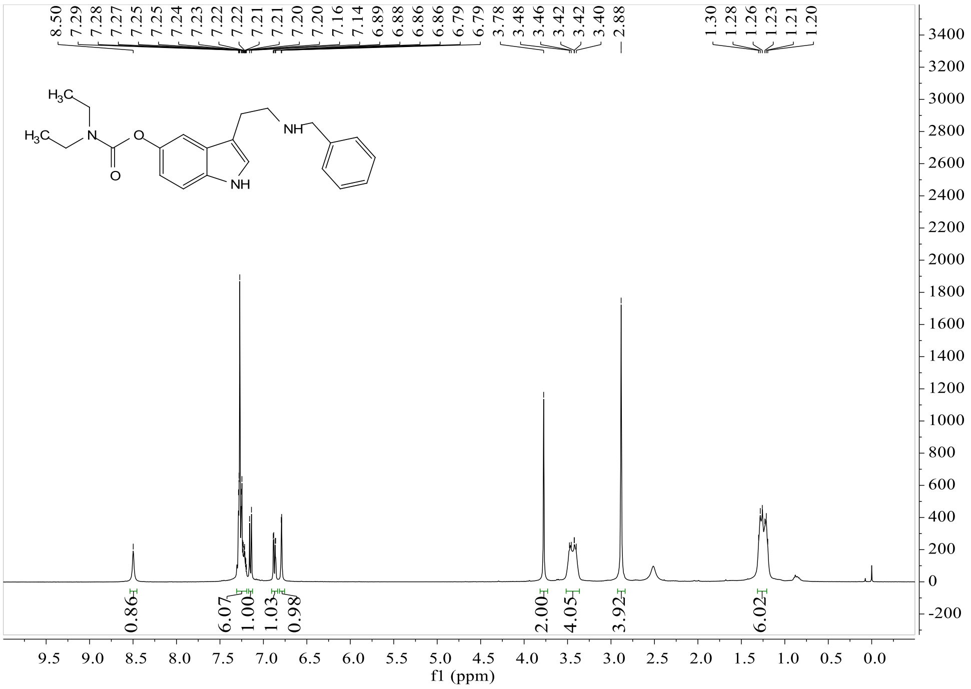

8.50
7.29
7.28
7.27
7.25
7.25
7.24
7.23
7.22
7.22
7.21
7.21
7.20
7.20
7.16
7.14
6.89
6.88
6.86
6.86
6.79
6.79
3.78
3.48
3.46
3.42
3.42
3.40
2.88
1.30
1.28
1.26
1.23
1.21
1.20
0.86
6.07
1.00
1.03
0.98
2.00
4.05
3.92
6.02
f1 (ppm)

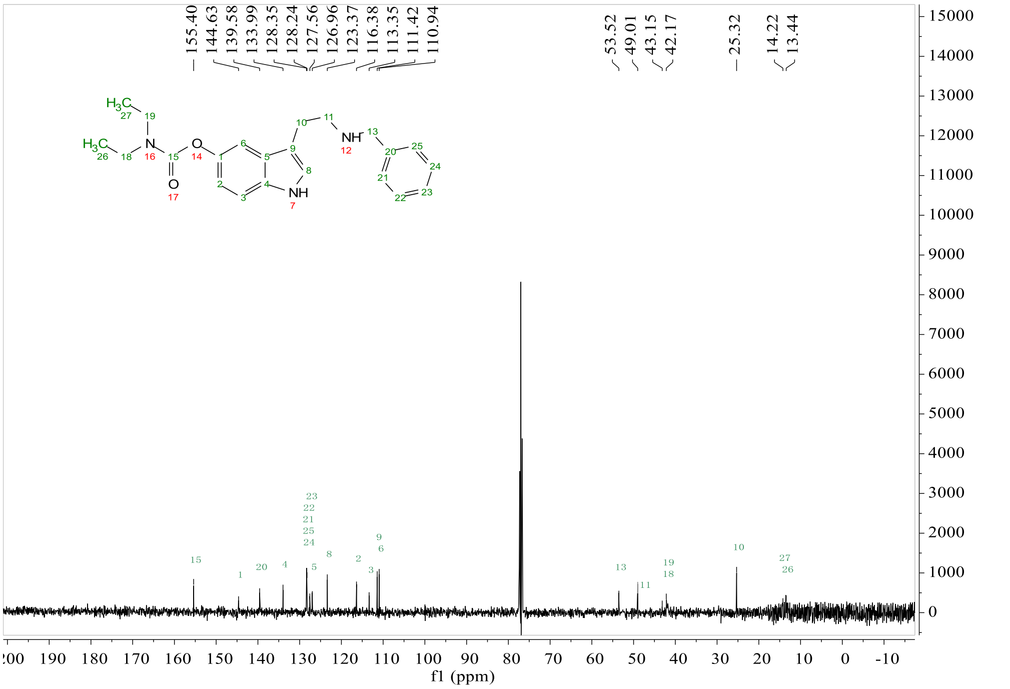

155.40
144.63
139.58
133.99
128.35
128.24
127.56
126.96
123.37
116.38
113.35
111.42
110.94
53.52
49.01
43.15
42.17
25.32
14.22
13.44
f1 (ppm)

3l:

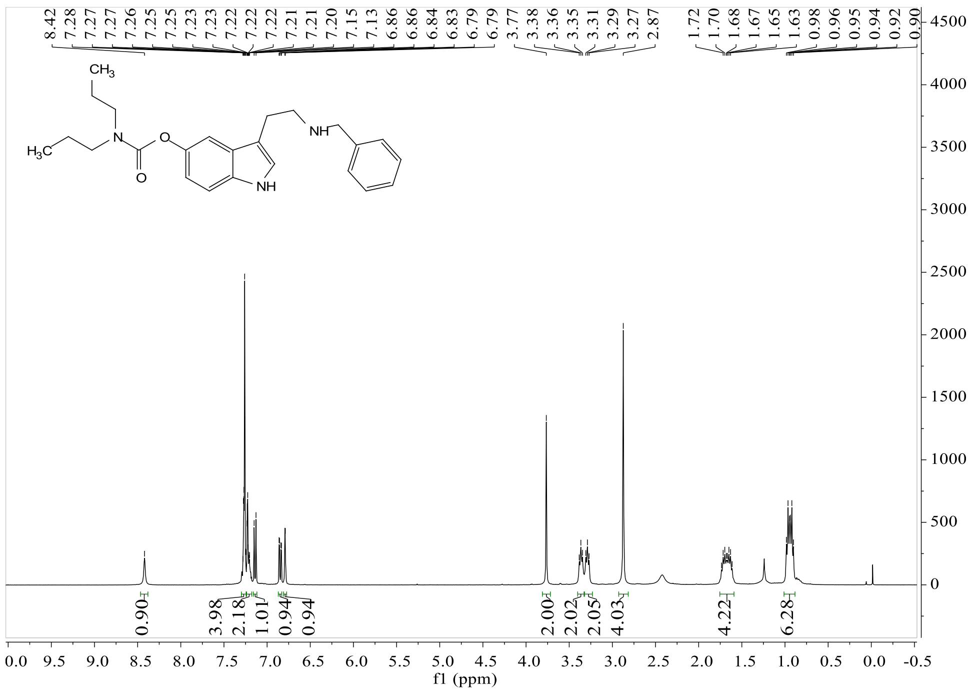

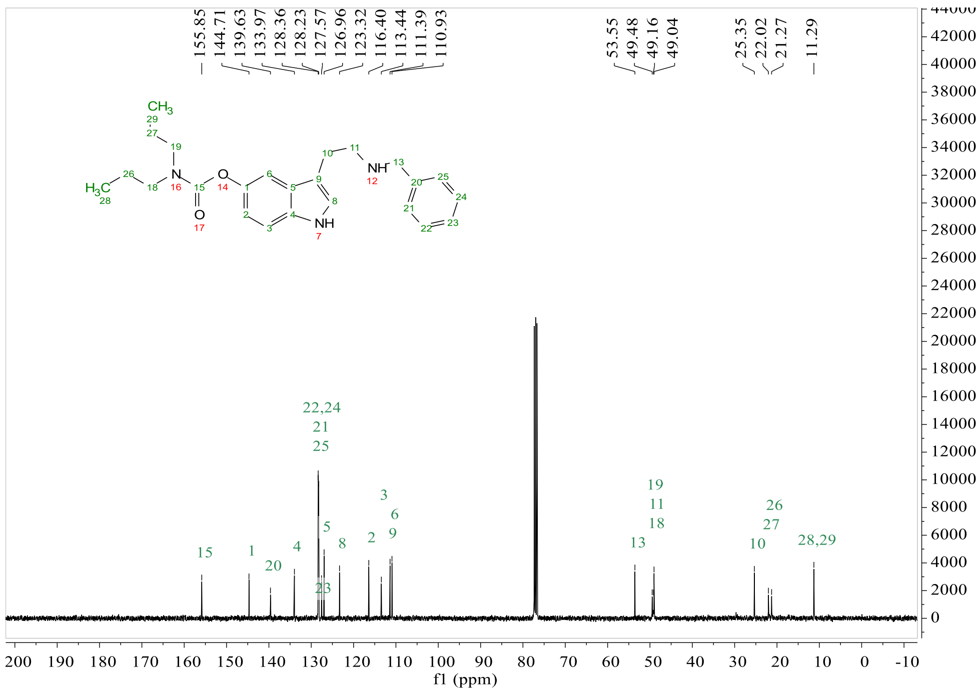

3m:

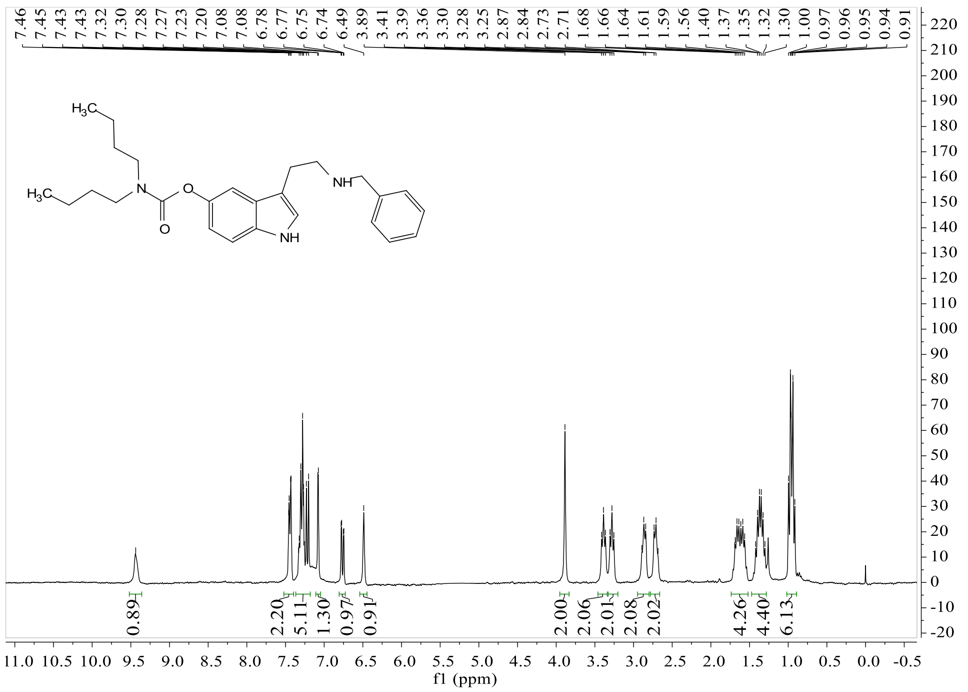

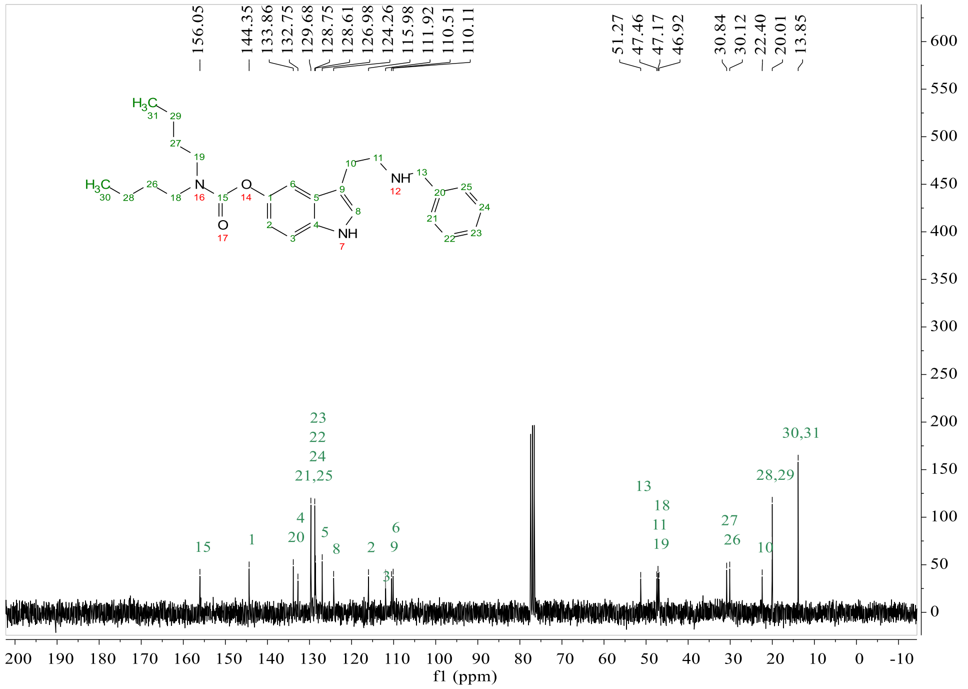

3n:

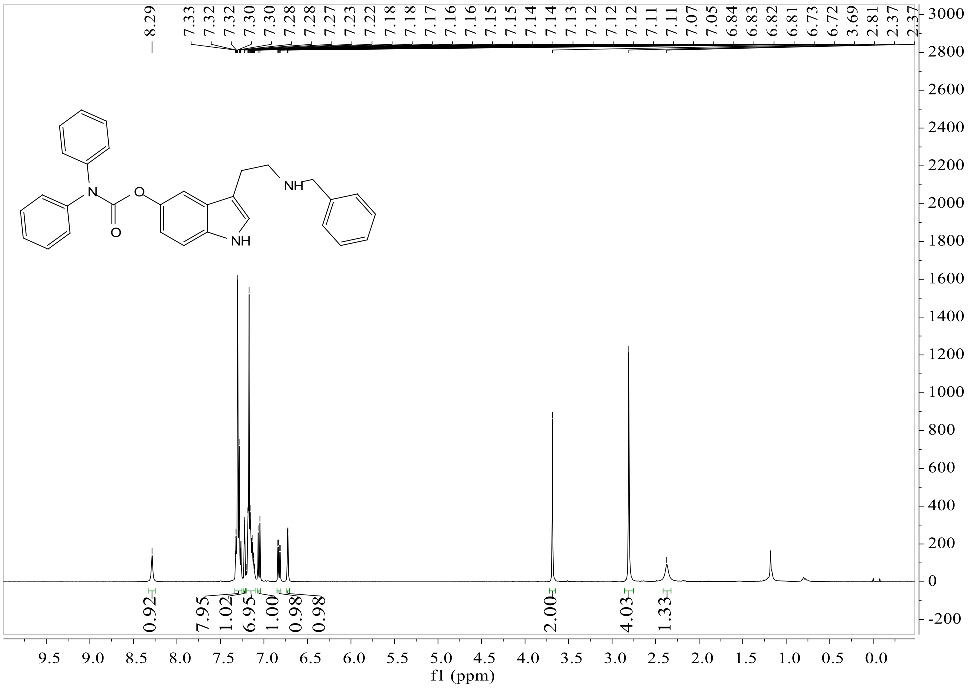

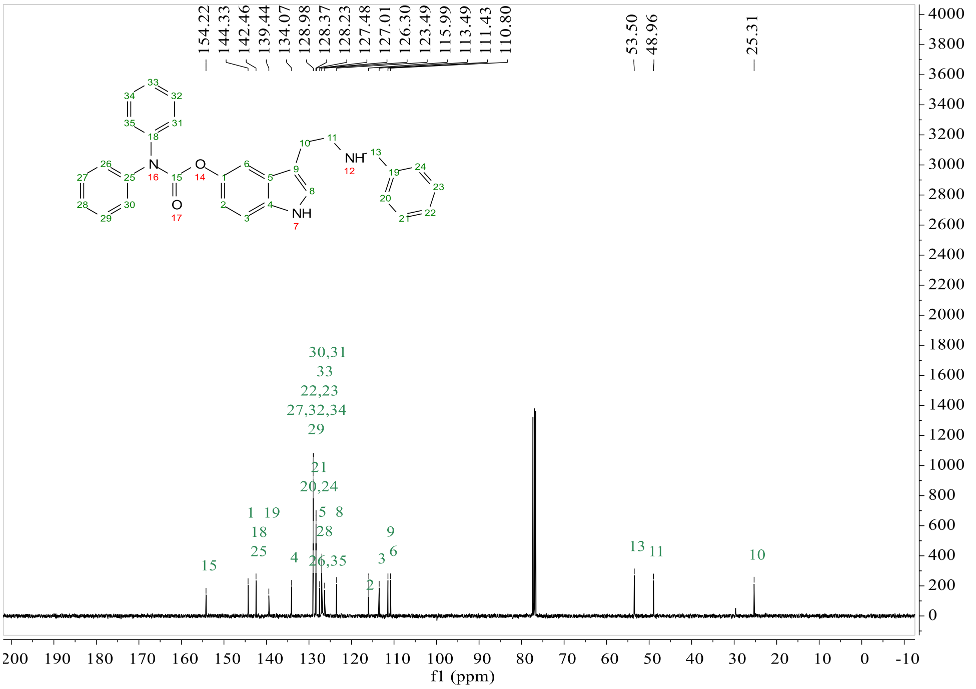

3o:

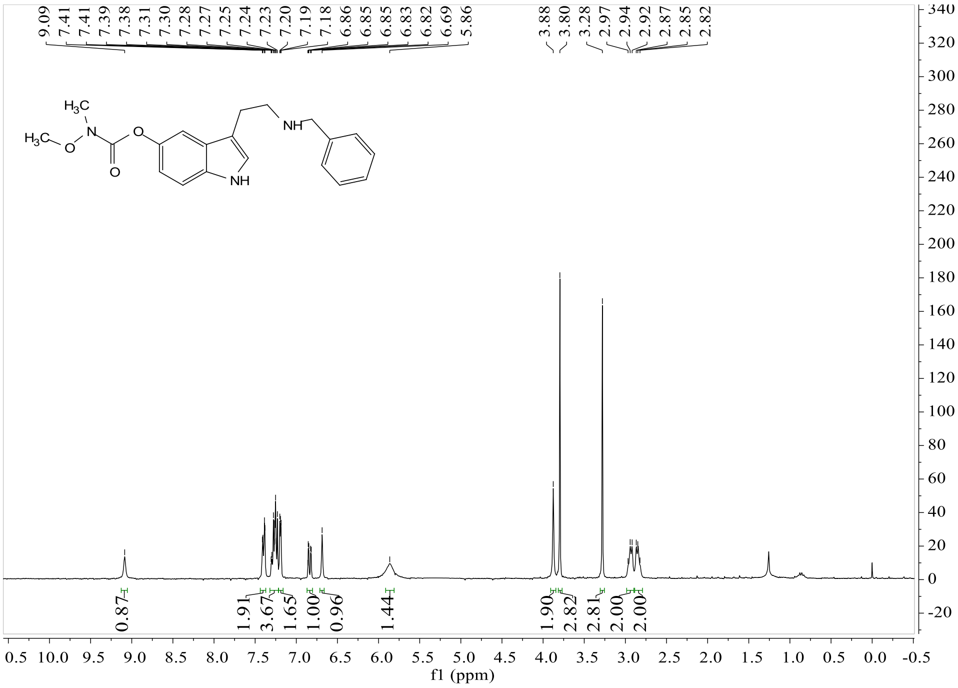

9.09 7.41 7.41 7.39 7.38 7.31 7.30 7.28 7.27 7.25 7.24 7.23 7.20 7.19 7.18 6.86 6.85 6.85 6.83 6.82 6.69 5.86
3.88 3.80 3.28 2.97 2.94 2.92 2.87 2.85 2.82
0.87 1.91 3.67 1.65 1.00 0.96 1.44 1.90 2.82 2.81 2.00 2.00
f1 (ppm)

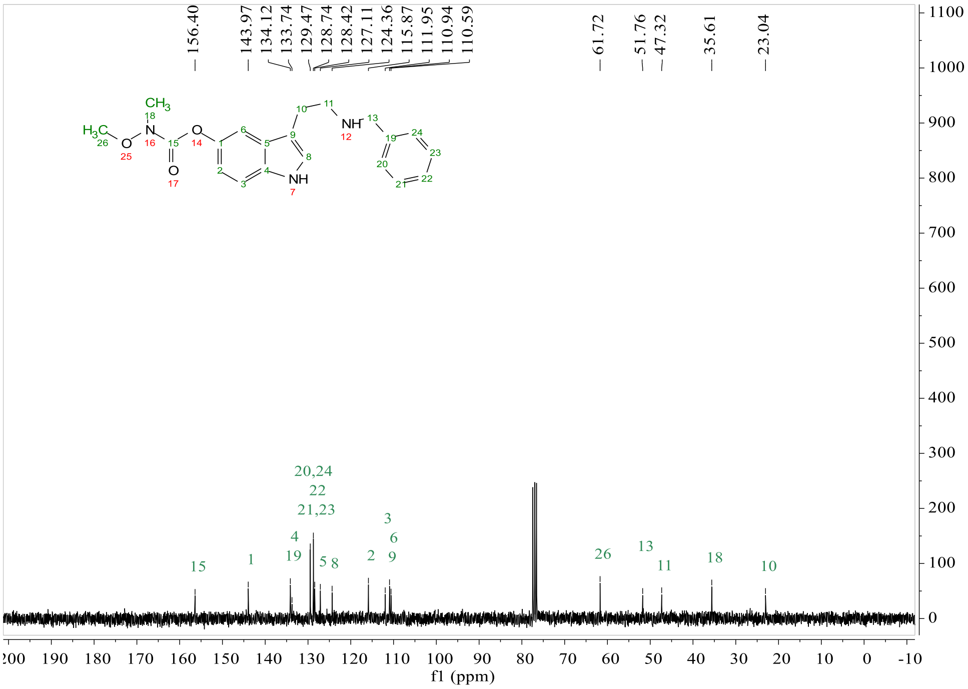

156.40 143.97 134.12 133.74 129.47 128.74 128.42 127.11 124.36 115.87 111.95 110.94 110.59
61.72 51.76 47.32 35.61 23.04
f1 (ppm)

3p:

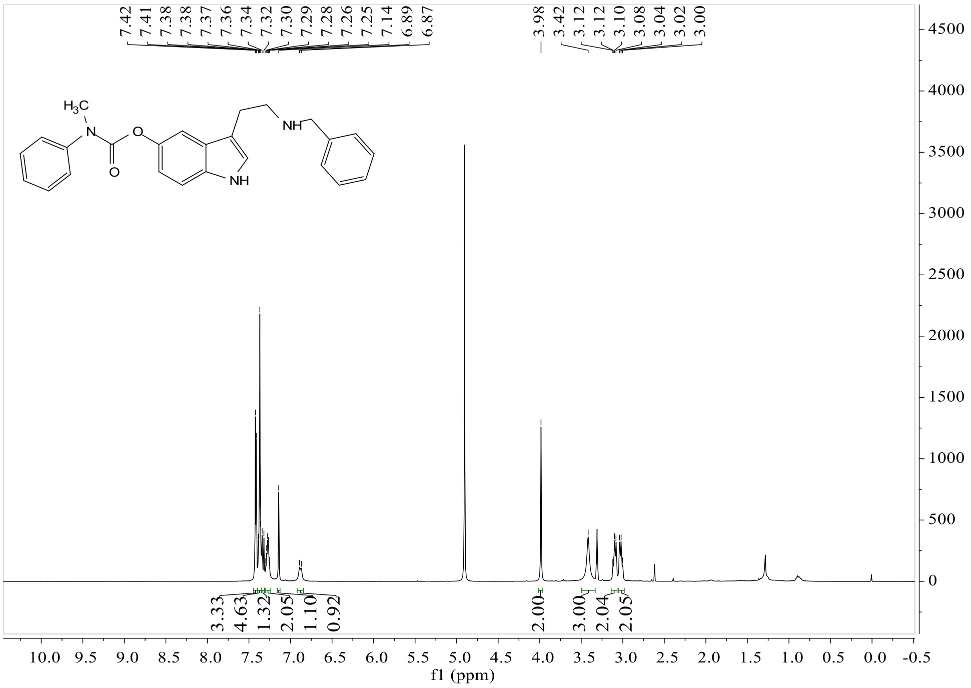

7.42
7.41
7.38
7.38
7.37
7.36
7.34
7.32
7.30
7.29
7.28
7.26
7.25
7.14
6.89
6.87
3.98
3.42
3.12
3.12
3.10
3.08
3.04
3.02
3.00
3.33
4.63
1.32
2.05
1.10
0.92
2.00
3.00
2.04
2.05
f1 (ppm)

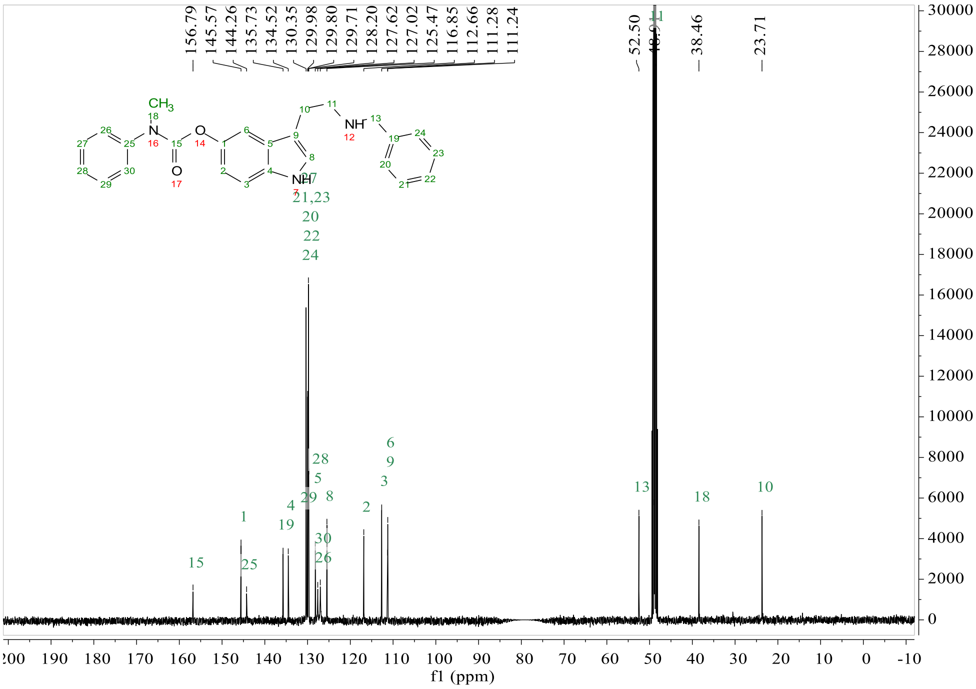

156.79
145.57
144.26
135.73
134.52
130.35
129.98
129.80
129.71
128.20
127.62
127.02
125.47
116.85
112.66
111.28
111.24
52.50
38.46
23.71
f1 (ppm)

3q:

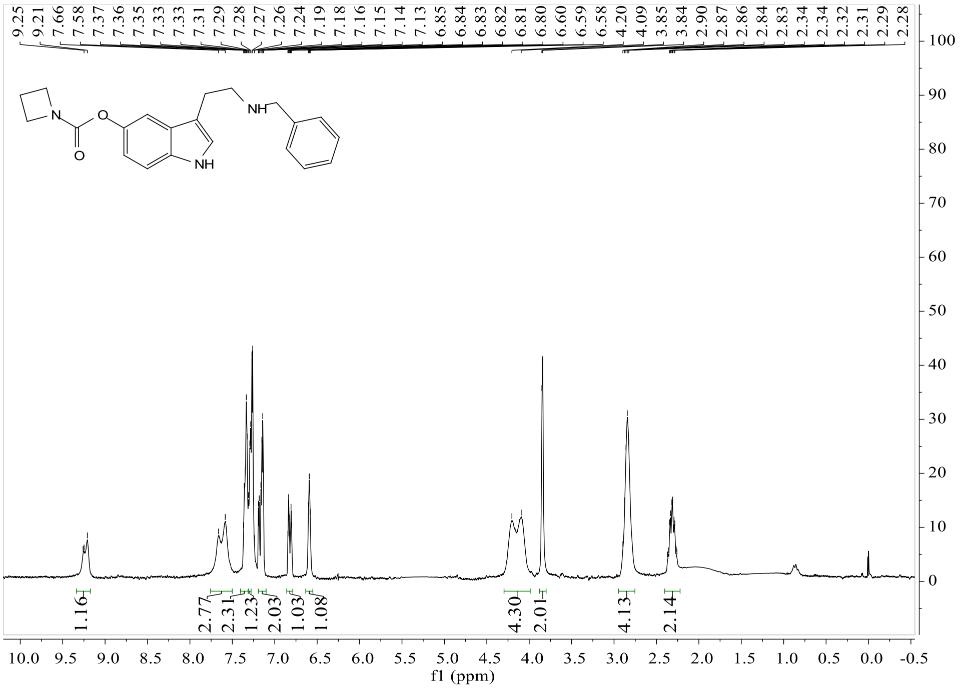
9.25
9.21
7.66
7.58
7.37
7.36
7.35
7.33
7.33
7.31
7.29
7.28
7.27
7.26
7.24
7.19
7.18
7.16
7.15
7.14
7.13
6.85
6.84
6.83
6.82
6.81
6.80
6.60
6.59
6.58
4.20
4.09
3.85
3.84
2.90
2.87
2.86
2.84
2.83
2.34
2.34
2.32
2.31
2.29
2.28
1.16
2.77
2.31
1.23
2.03
1.03
1.08
4.30
2.01
4.13
2.14
f1 (ppm)

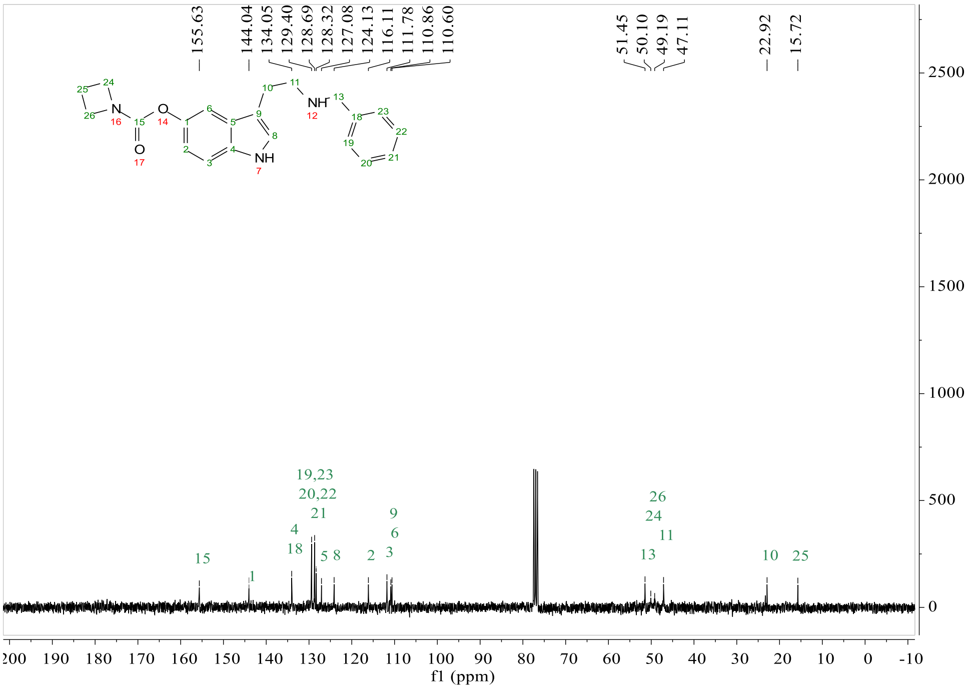
155.63
144.04
134.05
129.40
128.69
128.32
127.08
124.13
116.11
111.78
110.86
110.60
51.45
50.10
49.19
47.11
22.92
15.72
f1 (ppm)

3r:

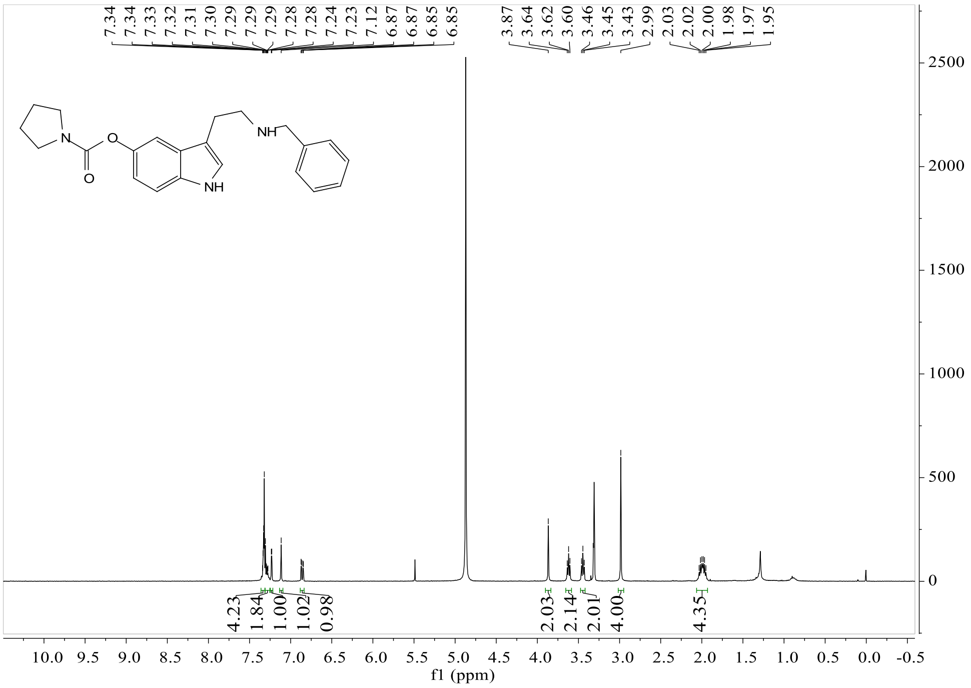
7.34
7.34
7.33
7.32
7.31
7.30
7.29
7.29
7.29
7.28
7.28
7.24
7.24
7.23
7.12
6.87
6.85
6.85
3.87
3.64
3.62
3.60
3.46
3.45
3.43
2.99
2.03
2.02
2.00
1.98
1.97
1.95
NH
N
O
O
NH
4.23
1.84
1.00
1.02
0.98
2.03
2.14
2.01
4.00
4.35
f1 (ppm)

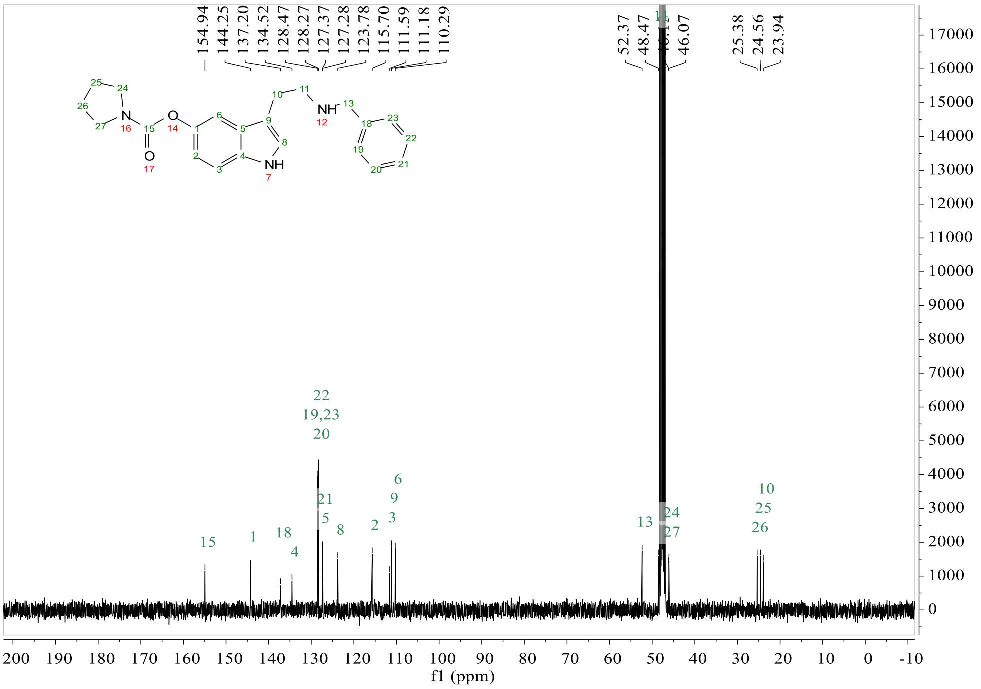
154.94
144.25
137.20
134.52
128.47
128.27
127.37
127.28
123.78
115.70
111.59
111.18
110.29
52.37
48.47
46.07
25.38
24.56
23.94
22
19,23
20
21
5
15
1
18
4
8
2
6
9
3
13
24
27
10
25
26
f1 (ppm)

3s:

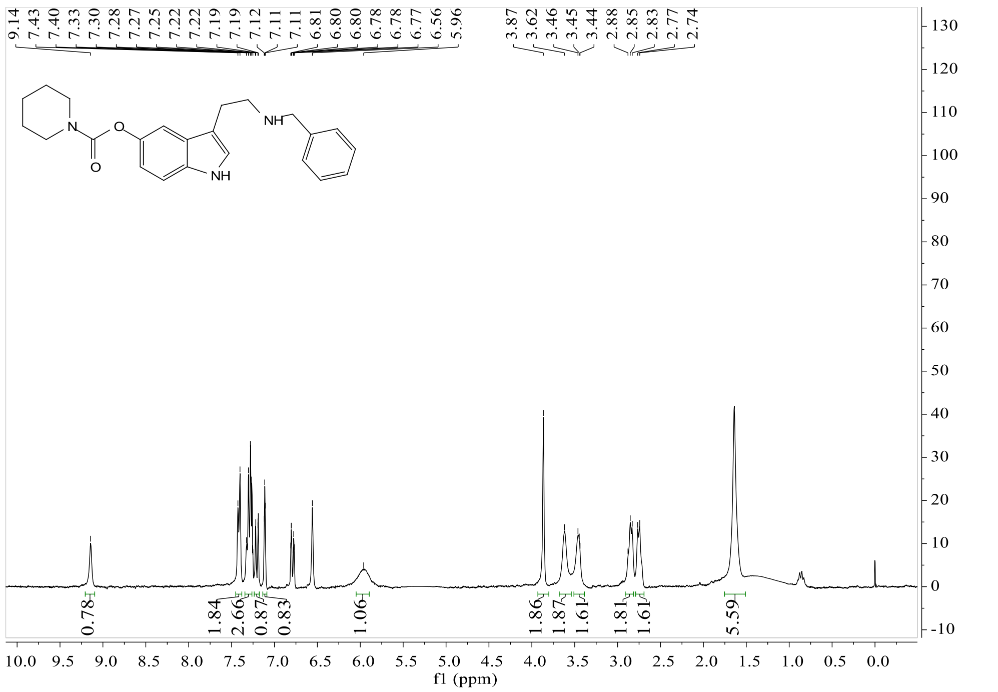

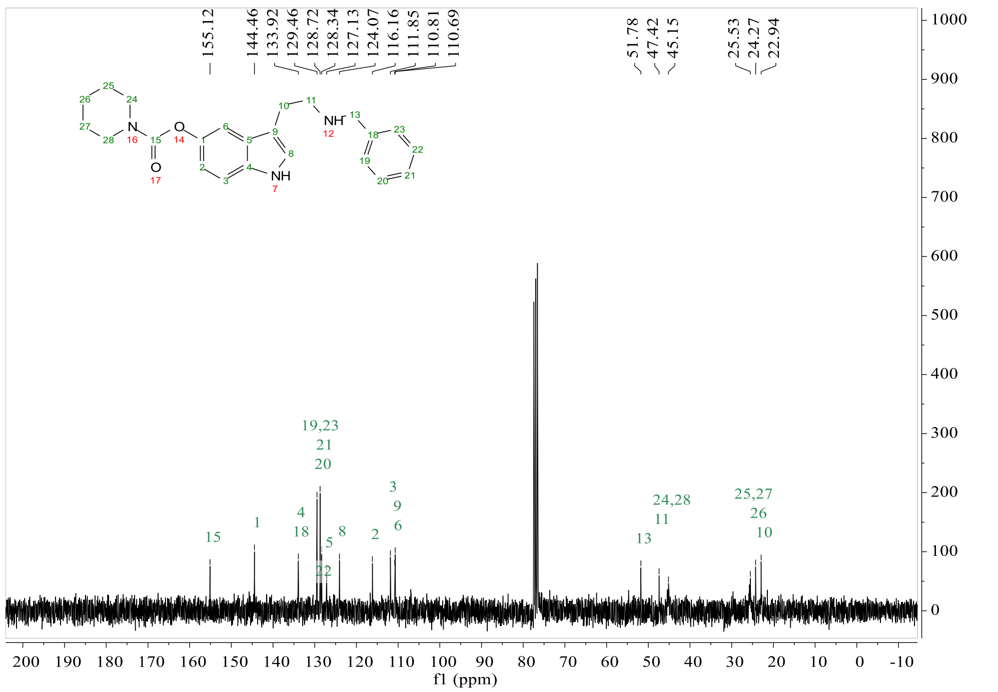

3t:

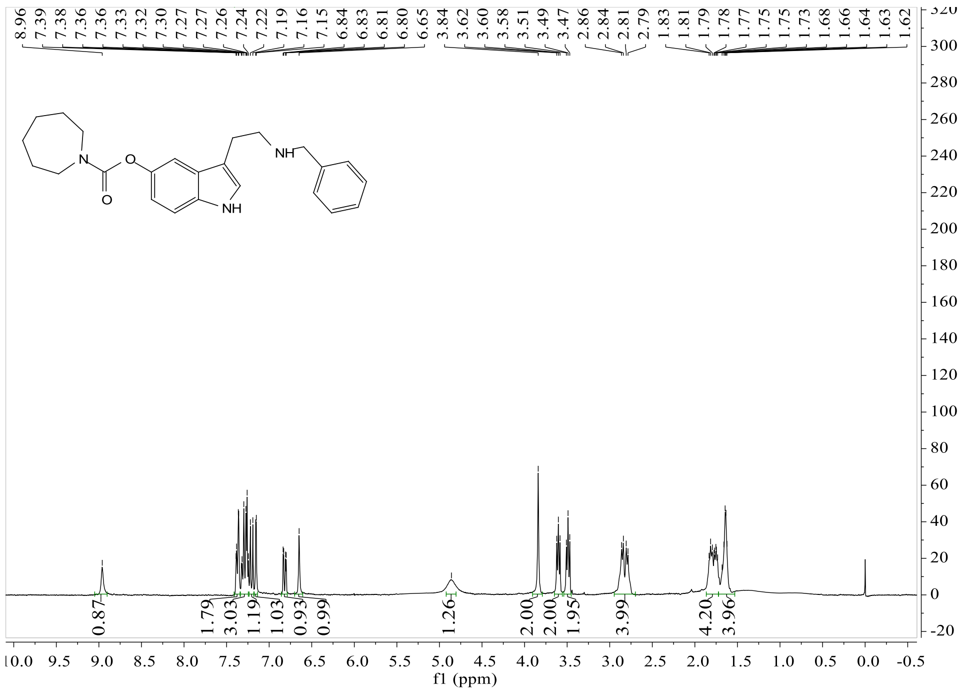

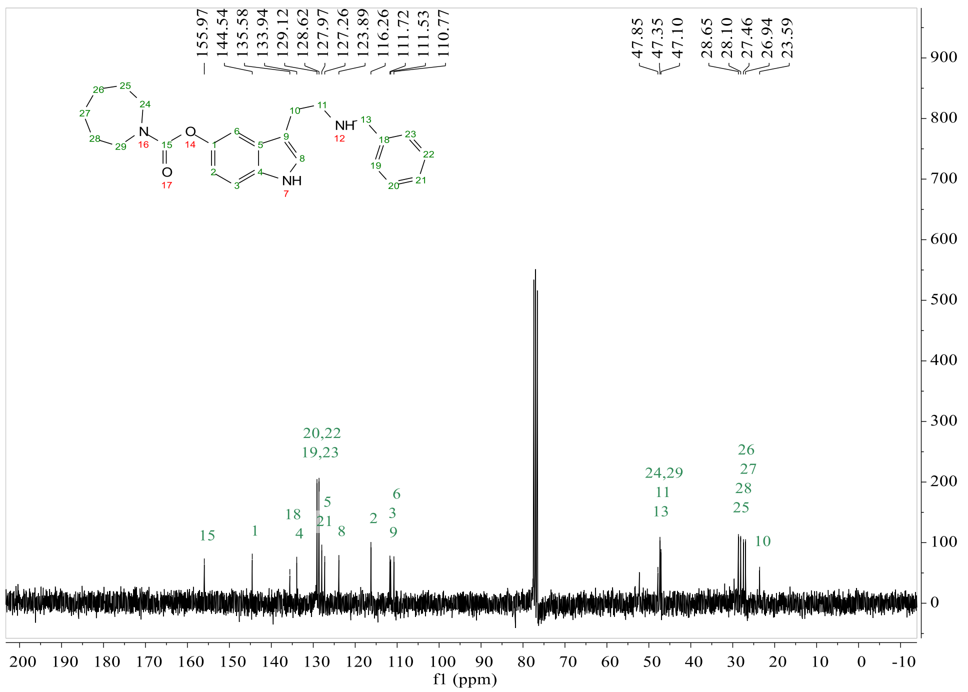

3u:

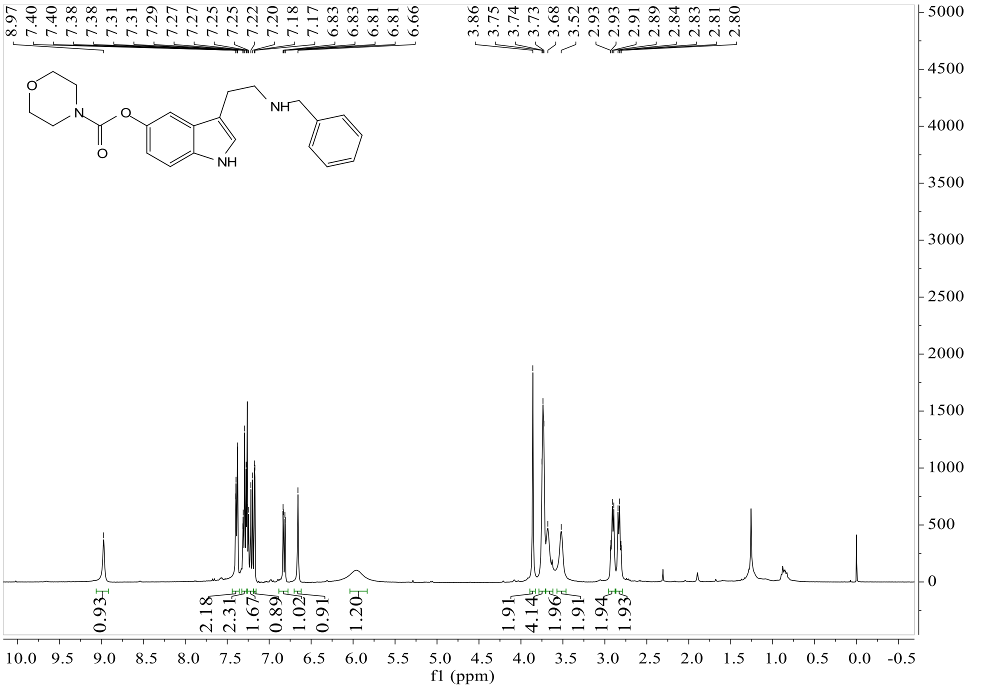
8.97 7.40 7.40 7.38 7.38 7.31 7.31 7.29 7.27 7.27 7.25 7.25 7.22 7.20 7.18 7.17 6.83 6.83 6.81 6.81 6.66
3.86 3.75 3.74 3.73 3.68 3.52 2.93 2.93 2.91 2.89 2.84 2.83 2.81 2.80
0.93 2.18 2.31 1.67 0.89 1.02 0.91 1.20 1.91 4.14 1.96 1.91 1.94 1.93
f1 (ppm)

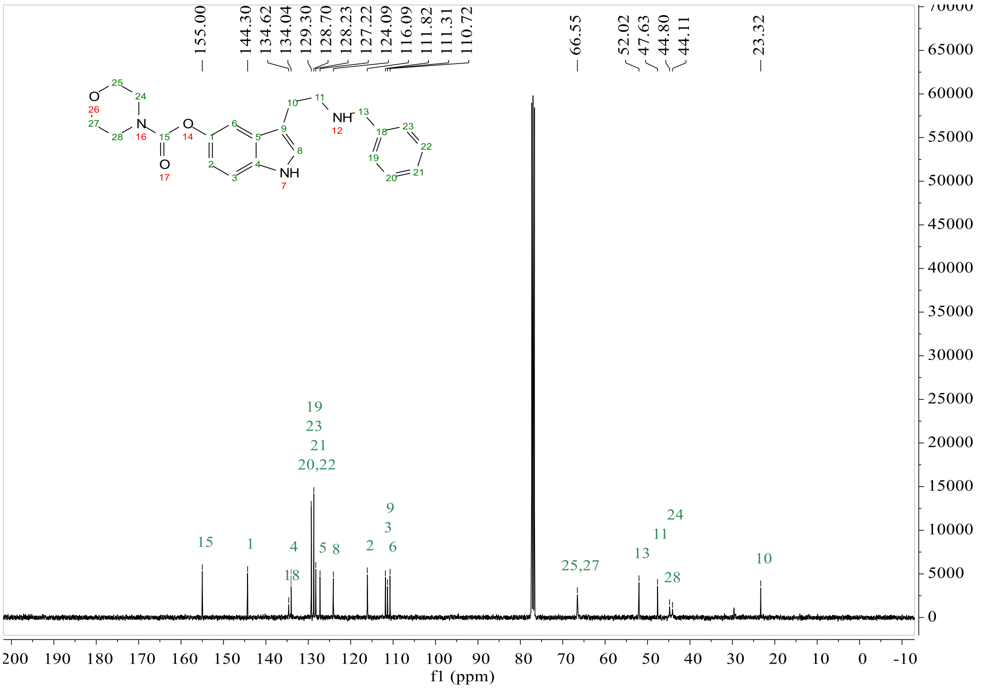
155.00 144.30 134.62 134.04 129.30 128.70 128.23 127.22 124.09 116.09 111.82 111.31 110.72
66.55 52.02 47.63 44.80 44.11 23.32
f1 (ppm)

4c:

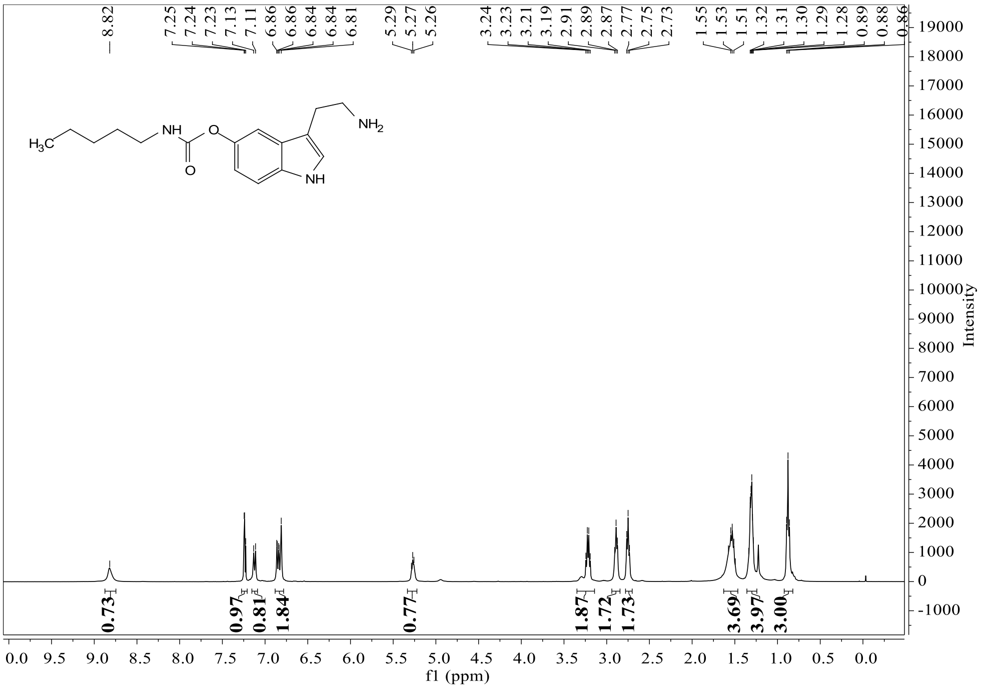

8.82
7.25
7.24
7.23
7.13
7.11
6.86
6.86
6.84
6.84
6.81
5.29
5.27
5.26
3.24
3.23
3.21
3.19
2.91
2.89
2.87
2.77
2.75
2.73
1.55
1.53
1.51
1.32
1.31
1.30
1.29
1.28
0.89
0.88
0.86
0.73
0.97
0.81
1.84
0.77
1.87
1.72
1.73
3.69
3.97
3.00
f1 (ppm)
Intensity
NH
O
O
NH
NH2
H3C

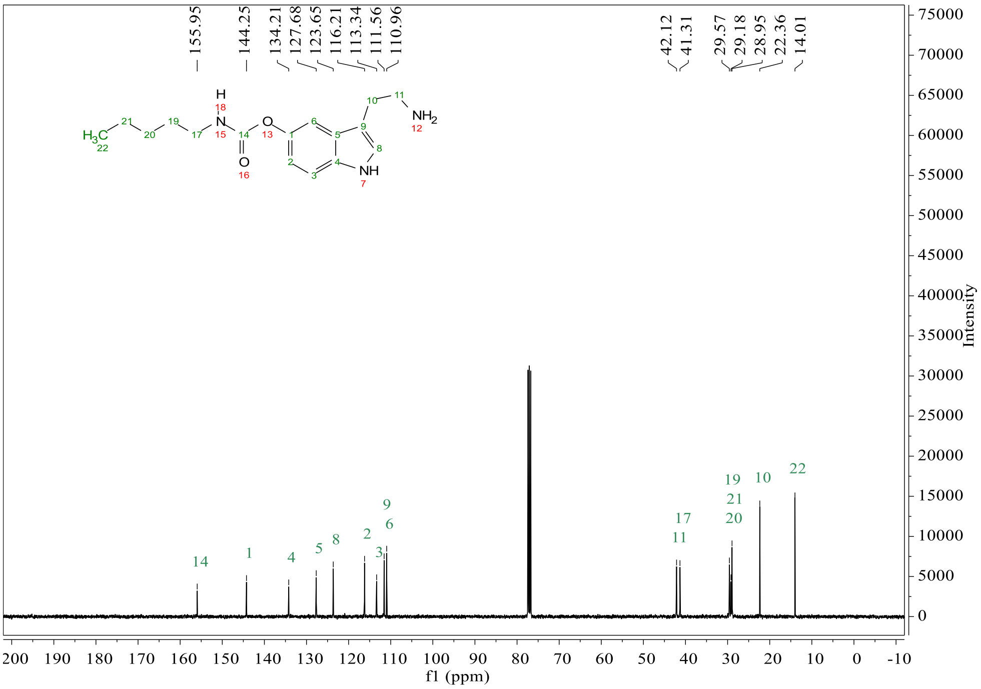

155.95
144.25
134.21
127.68
123.65
116.21
113.34
111.56
110.96
42.12
41.31
29.57
29.18
28.95
22.36
14.01
f1 (ppm)
Intensity
14
1
4
5
8
2
3
9
6
17
11
19
21
20
10
22

4d:

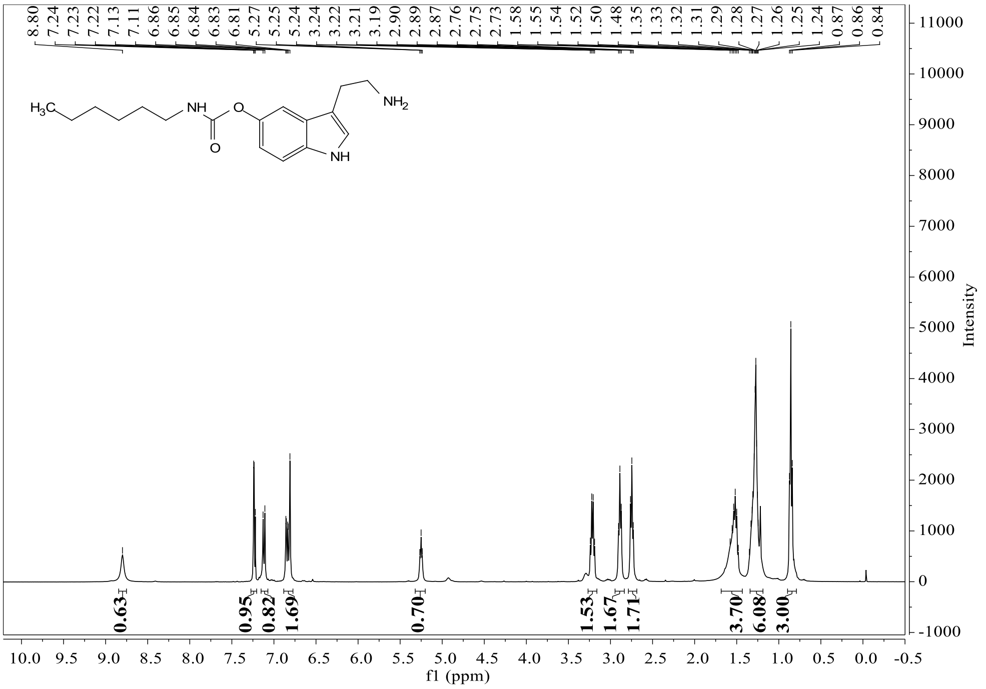

8.80 7.24 7.23 7.22 7.13 7.11 6.86 6.85 6.84 6.83 6.81 5.27 5.25 5.24 3.24 3.22 3.21 3.19 2.90 2.89 2.87 2.76 2.75 2.73 1.58 1.55 1.54 1.52 1.50 1.48 1.35 1.33 1.32 1.31 1.29 1.28 1.27 1.26 1.25 1.24 0.87 0.86 0.84
0.63 0.95 0.82 1.69 0.70 1.53 1.67 1.71 3.70 6.08 3.00
f1 (ppm)
Intensity

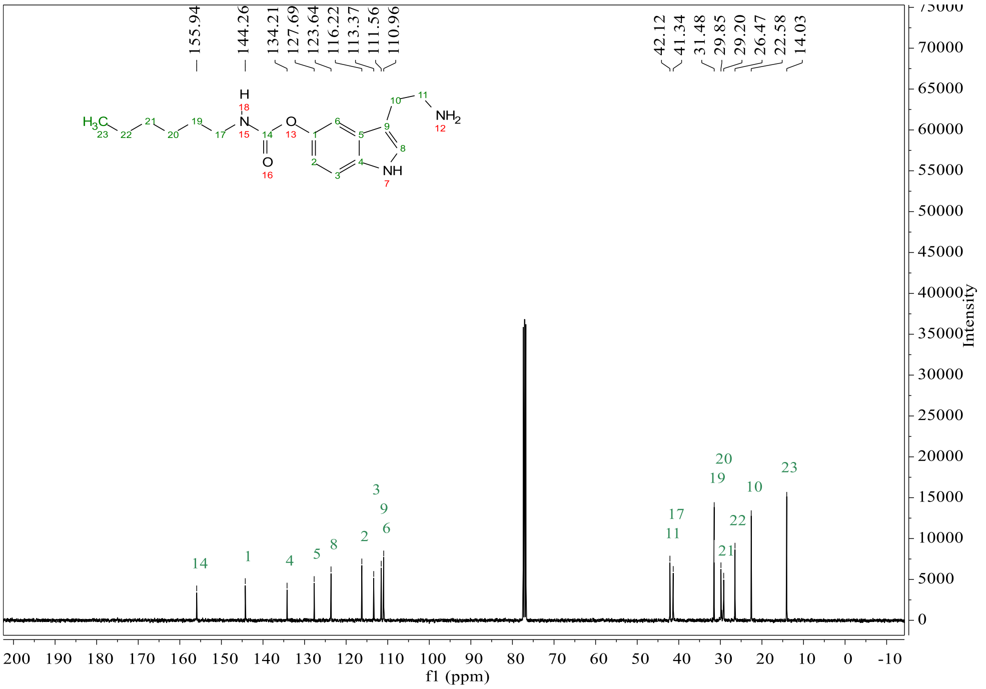

155.94 144.26 134.21 127.69 123.64 116.22 113.37 111.56 110.96 42.12 41.34 31.48 29.85 29.20 26.47 22.58 14.03
f1 (ppm)
Intensity

4e:

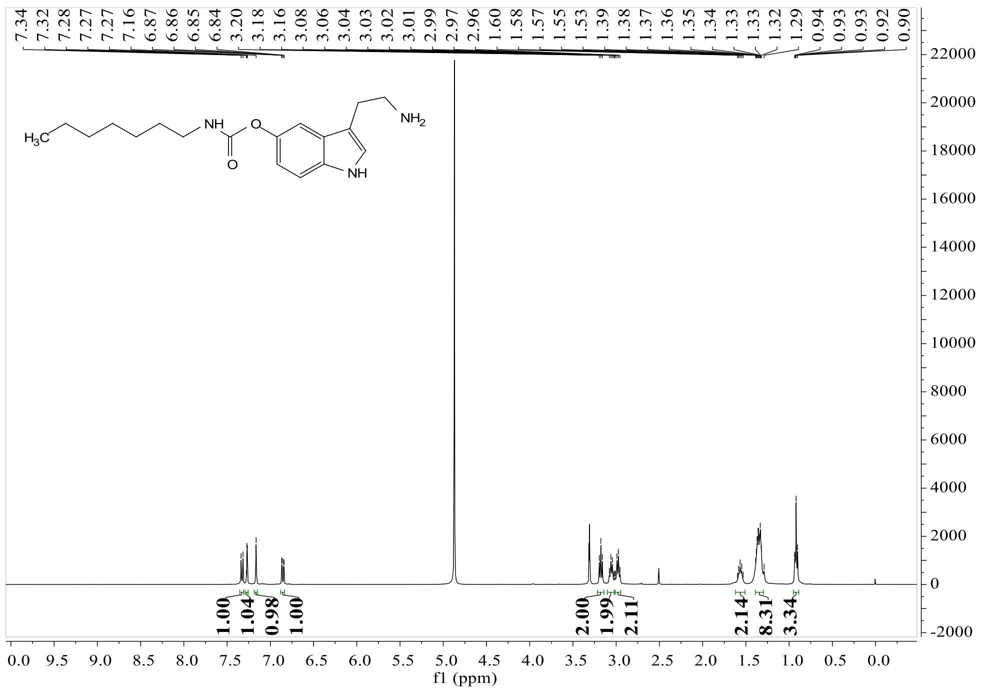

7.34
7.32
7.28
7.27
7.27
7.16
6.87
6.86
6.85
6.84
3.20
3.18
3.16
3.08
3.06
3.04
3.03
3.02
3.01
2.99
2.97
2.96
1.60
1.58
1.57
1.55
1.53
1.39
1.38
1.37
1.36
1.35
1.34
1.33
1.33
1.32
1.29
0.94
0.93
0.93
0.92
0.90
1.00
1.04
0.98
1.00
2.00
1.99
2.11
2.14
8.31
3.34
f1 (ppm)

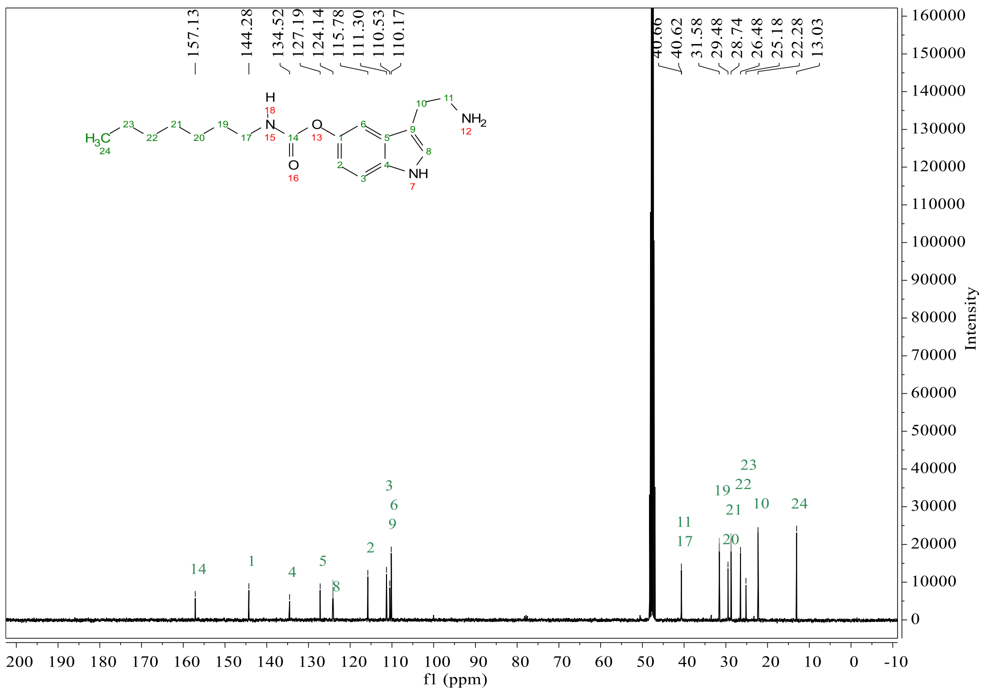

157.13
144.28
134.52
127.19
124.14
115.78
111.30
110.53
110.17
40.66
40.62
31.58
29.48
28.74
26.48
25.18
22.28
13.03
Intensity
f1 (ppm)

HPLC results of synthesized compounds

*Compound **2a** (96.11%)*

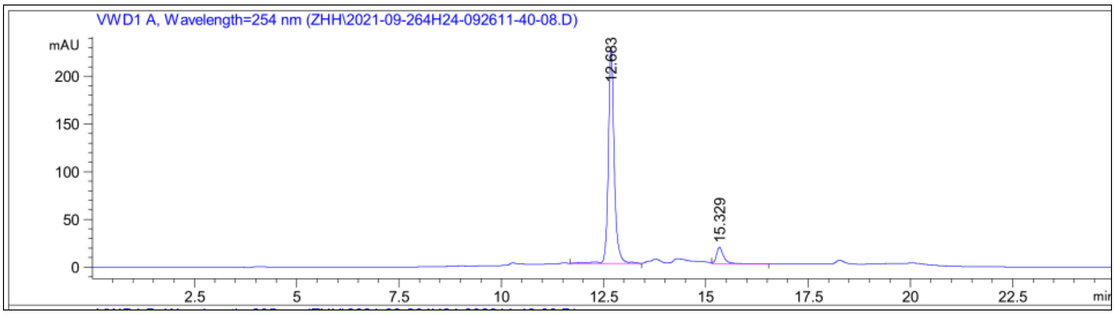

Peak #	RetTime [min]	Type	Width [min]	Area [mAU*s]	Height [mAU]	Area %
1	12.683	VV R	0.1489	2266.37378	226.24910	96.1103
2	15.329	MM	0.1263	91.72200	12.10147	3.8897

*Compound **2b** (96.18%)*

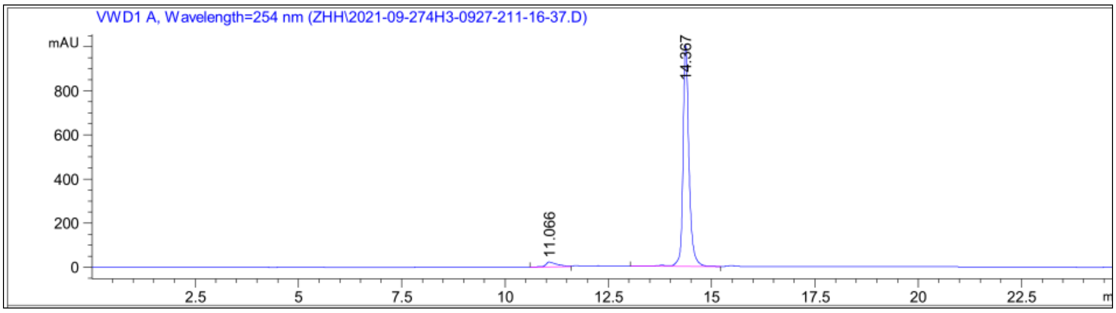

Peak #	RetTime [min]	Type	Width [min]	Area [mAU*s]	Height [mAU]	Area %
1	11.066	BV	0.2535	402.79239	21.40935	3.8194
2	14.367	VB R	0.1508	1.01431e4	1002.57697	96.1806

*Compound **2c** (97.87%)*

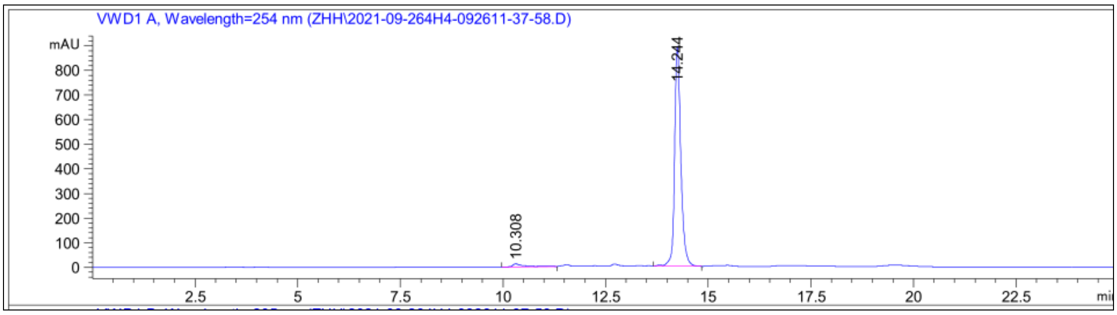

Peak #	RetTime [min]	Type	Width [min]	Area [mAU*s]	Height [mAU]	Area %
1	10.308	BV R	0.2387	204.70920	11.66859	2.1318
2	14.244	VB R	0.1564	9397.82422	892.25366	97.8682

*Compound **2d** (95.35%)*

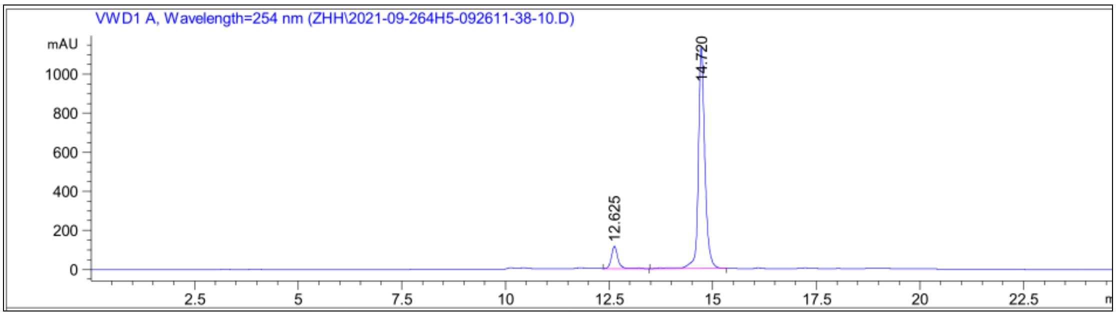

Peak #	RetTime [min]	Type	Width [min]	Area [mAU*s]	Height [mAU]	Area %
1	12.625	MM	0.1227	607.81915	82.54528	4.6466
2	14.720	VB R	0.1630	1.24731e4	1137.63208	95.3534

*Compound **2f** (97.38%)*

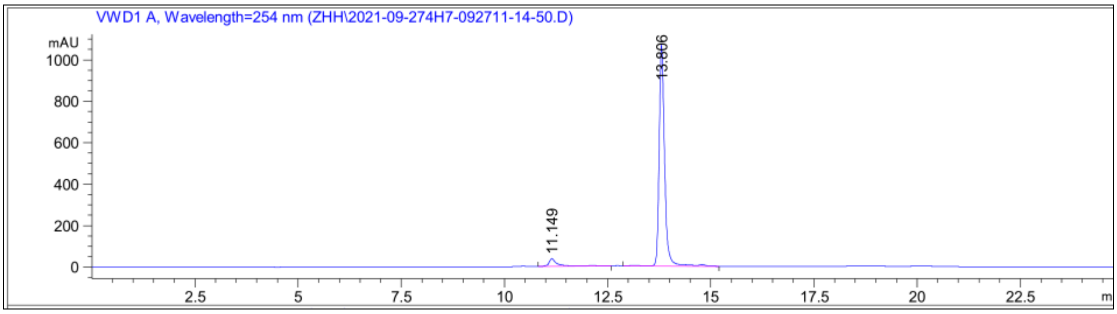

Peak #	RetTime [min]	Type	Width [min]	Area [mAU*s]	Height [mAU]	Area %
1	11.149	MM	0.1324	265.27377	33.40529	2.6171
2	13.806	VV R	0.1389	9871.05371	1067.05896	97.3829

*Compound **2h** (96.09%)*

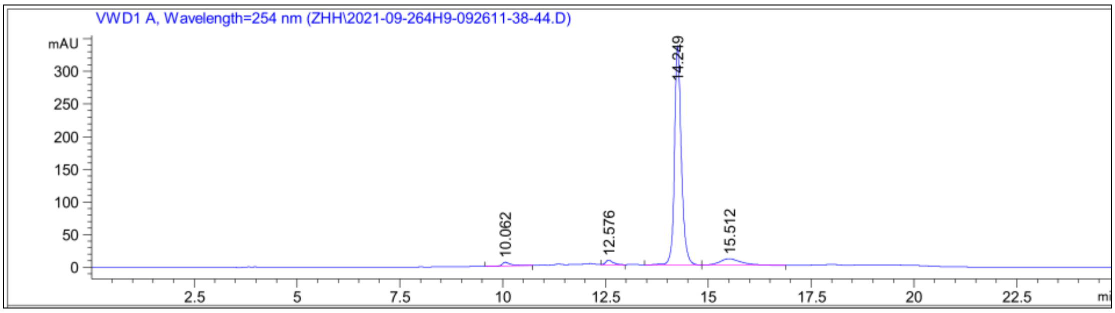

Peak #	RetTime [min]	Type	Width [min]	Area [mAU*s]	Height [mAU]	Area %
1	10.062	MM	0.1144	34.73880	5.06237	0.8603
2	12.576	MM	0.1205	39.93761	5.52498	0.9891
3	14.249	VV R	0.1751	3880.01563	334.99265	96.0890
4	15.512	MM	0.2606	83.24635	5.32469	2.0616

*Compound **2i** (95.16%)*

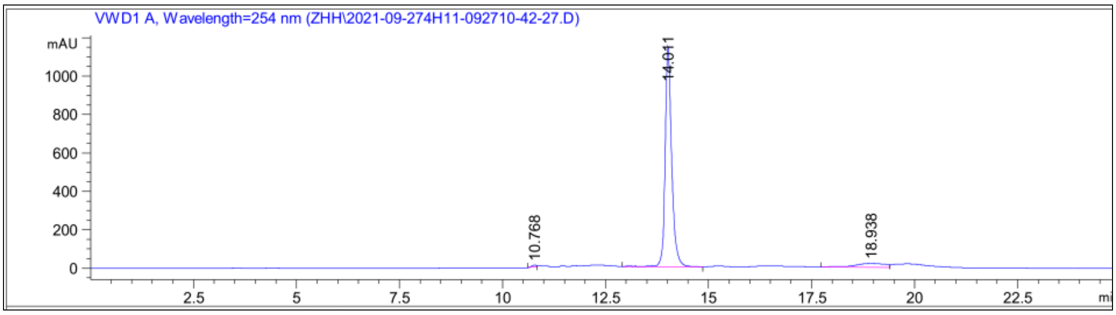

Peak #	RetTime [min]	Type	Width [min]	Area [mAU*s]	Height [mAU]	Area %
1	10.768	MM	0.2351	252.67273	17.90887	1.9090
2	14.011	VB R	0.1620	1.25960e4	1146.89172	95.1639
3	18.938	MM	0.5379	387.43594	12.00535	2.9271

*Compound **2j** (95.75%)*

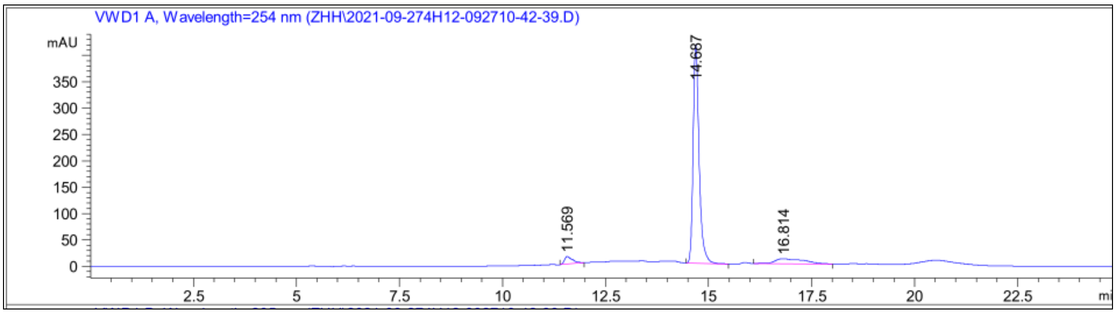

Peak #	RetTime [min]	Type	Width [min]	Area [mAU*s]	Height [mAU]	Area %
1	11.569	MM	0.1636	104.30507	10.62770	2.3837
2	14.687	BV R	0.1522	4189.71484	413.61844	95.7491
3	16.814	MM	0.2877	81.70151	4.73286	1.8672

*Compound **2l** (96.50%)*

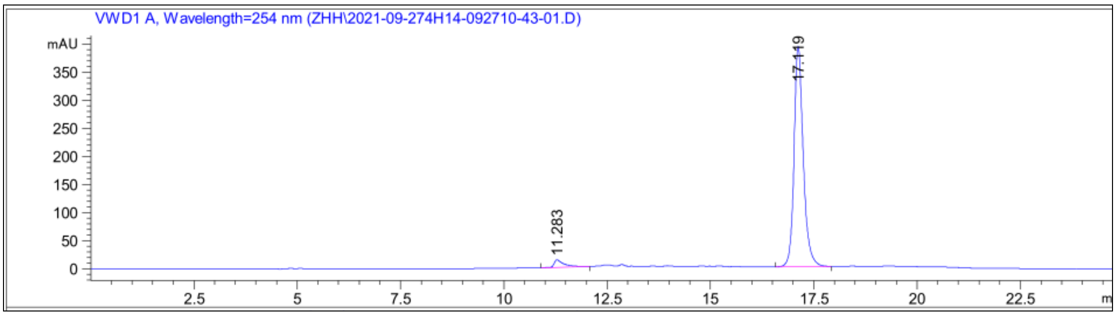

Peak #	RetTime [min]	Type	Width [min]	Area [mAU*s]	Height [mAU]	Area %
1	11.283	BV R	0.2345	219.64853	13.55718	3.4951
2	17.119	BB	0.1522	6064.74805	391.89551	96.5049

*Compound **2m** (98.08%)*

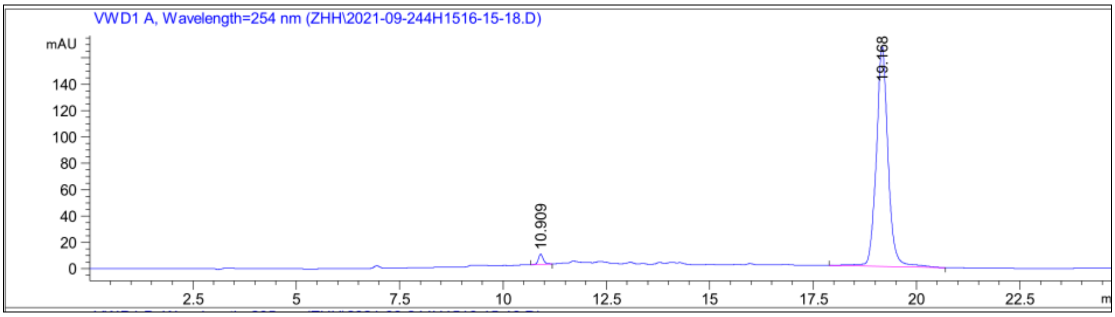

Peak #	RetTime [min]	Type	Width [min]	Area [mAU*s]	Height [mAU]	Area %
1	10.909	BB	0.1288	64.51392	7.77818	1.9161
2	19.168	BB	0.2929	3302.45190	166.83865	98.0839

*Compound **2n** (98.89%)*

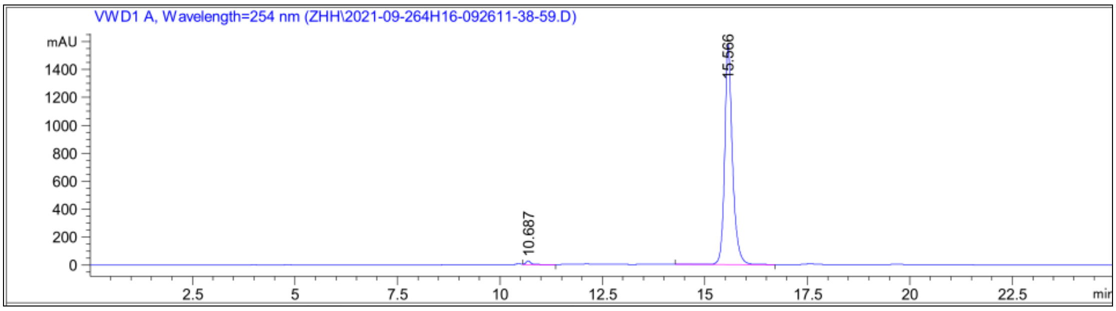

Peak #	RetTime [min]	Type	Width [min]	Area [mAU*s]	Height [mAU]	Area %
1	10.687	VV R	0.1319	234.16629	26.81316	1.1142
2	15.566	VB R	0.1937	2.07831e4	1575.46240	98.8858

*Compound **2o** (96.70%)*

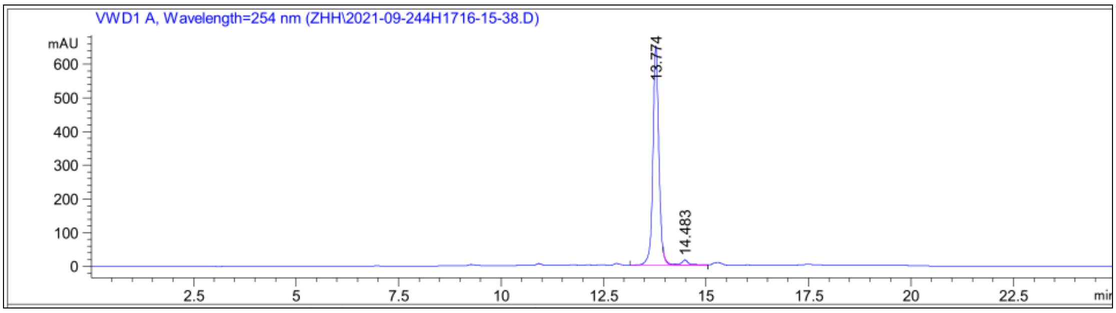

Peak #	RetTime [min]	Type	Width [min]	Area [mAU*s]	Height [mAU]	Area %
1	13.774	BV R	0.1523	6594.06055	650.35938	96.7037
2	14.483	VB E	0.2043	224.76872	15.22207	3.2963

Compound ***2p*** *(96.90%)*

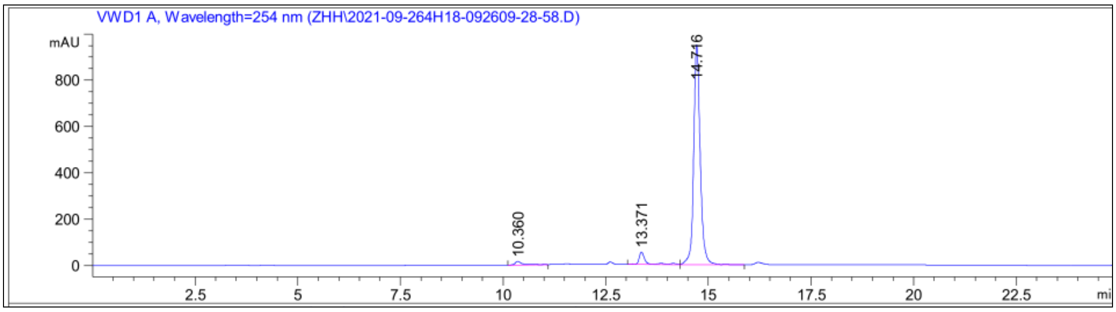

Peak #	RetTime [min]	Type	Width [min]	Area [mAU*s]	Height [mAU]	Area %
1	10.360	MM	0.0947	94.33377	16.60680	0.8769
2	13.371	MM	0.1025	238.92009	38.84423	2.2209
3	14.716	VV R	0.1641	1.04247e4	949.26740	96.9023

Compound ***2q*** *(95.27%)*

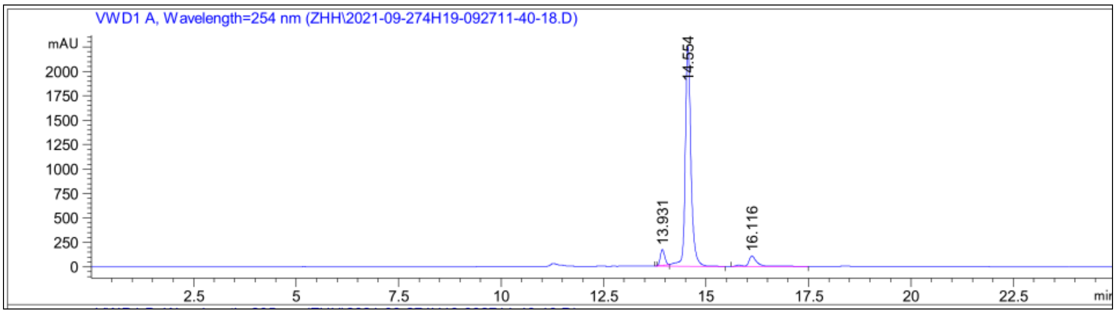

Peak #	RetTime [min]	Type	Width [min]	Area [mAU*s]	Height [mAU]	Area %
1	13.931	MM	0.0839	506.04095	100.47110	2.1401
2	14.554	MM	0.1658	2.25275e4	2264.38281	95.2712
3	16.116	MM	0.1241	612.12476	82.21546	2.5887

Compound ***2s*** *(96.07%)*

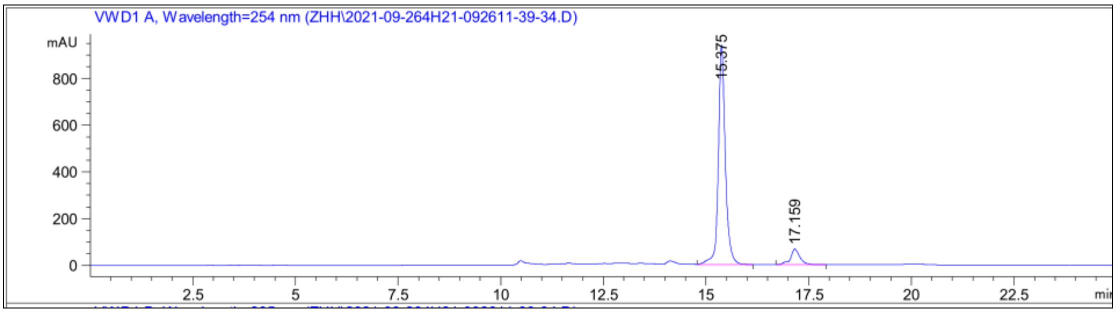

Peak #	RetTime [min]	Type	Width [min]	Area [mAU*s]	Height [mAU]	Area %
1	15.375	BB	0.1733	1.10653e4	940.56366	96.0703
2	17.159	MM	0.1574	452.62076	47.94029	3.9297

*Compound **2t** (96.25%)*

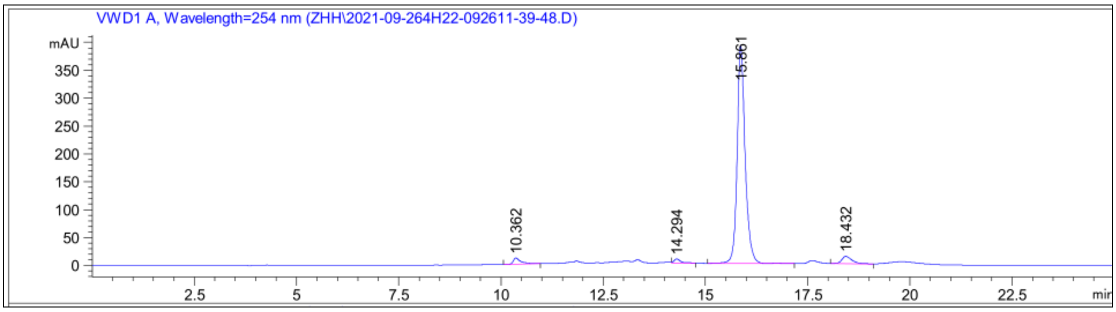

Peak #	RetTime [min]	Type	Width [min]	Area [mAU*s]	Height [mAU]	Area %
1	10.362	MM	0.1293	73.10896	9.42502	1.3246
2	14.301	MM	0.1101	35.05720	5.30745	0.6352
3	15.861	BV R	0.2009	5312.41699	390.04886	96.2511
4	18.432	MM	0.1655	98.75054	9.94713	1.7892

*Compound **3c** (95.56%)*

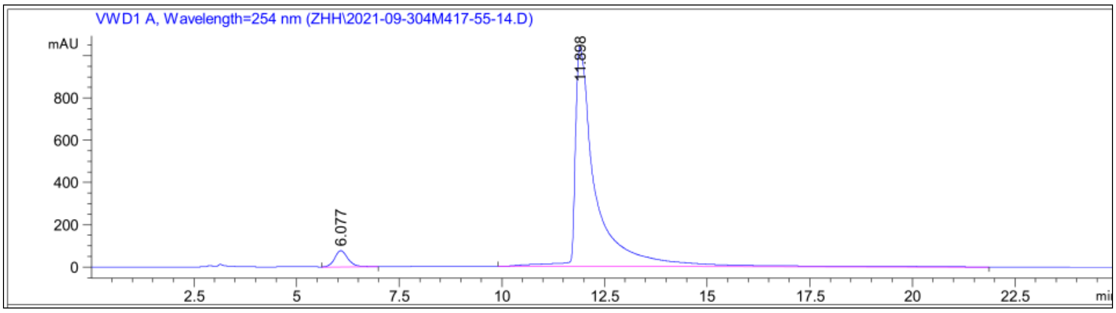

Peak #	RetTime [min]	Type	Width [min]	Area [mAU*s]	Height [mAU]	Area %
1	6.077	BB	0.3405	1709.30701	76.54395	4.4392
2	11.898	BB	0.4663	3.67957e4	1041.10461	95.5608

*Compound **3d** (98.76%)*

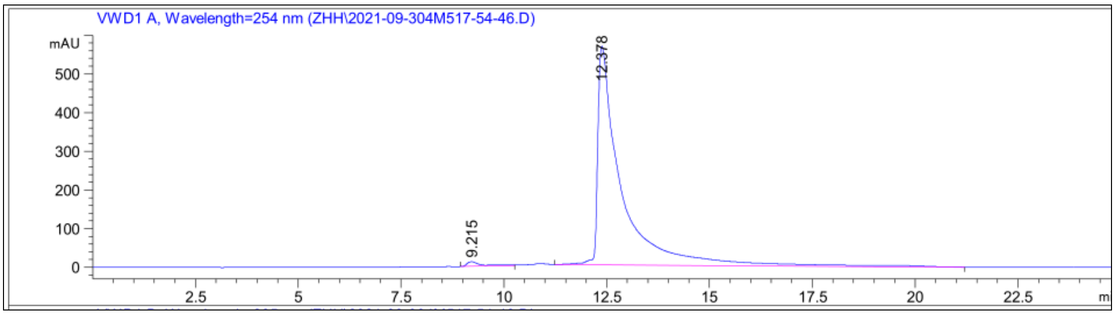

Peak #	RetTime [min]	Type	Width [min]	Area [mAU*s]	Height [mAU]	Area %
1	9.215	BV R	0.2957	284.24548	11.61274	1.2355

2	12.378	BB	0.5412	2.27219e4	564.98132	98.7645

*Compound **3f** (96.98%)*

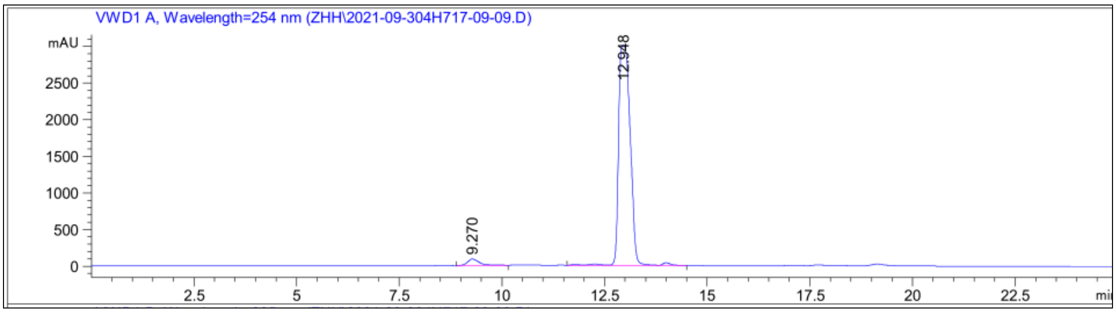

Peak #	RetTime [min]	Type	Width [min]	Area [mAU*s]	Height [mAU]	Area %
1	9.270	BB	0.3084	1840.30713	90.04299	3.0238
2	12.948	VV R	0.3031	5.90210e4	3003.79297	96.9762

*Compound **3k** (98.16%)*

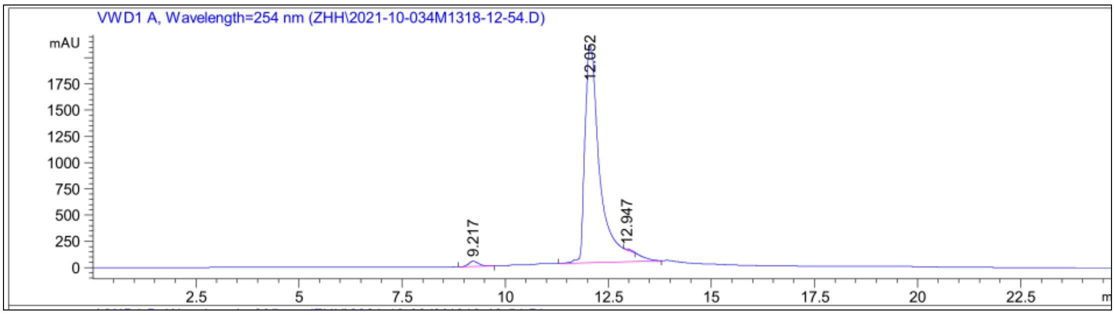

Peak #	RetTime [min]	Type	Width [min]	Area [mAU*s]	Height [mAU]	Area %
1	9.217	BV R	0.2396	858.72552	51.21434	1.5880
2	12.052	BV R	0.3873	5.30844e4	2069.25586	98.1642
3	12.947	VB E	0.1833	134.00050	9.93756	0.2478

*Compound **3l** (97.66%)*

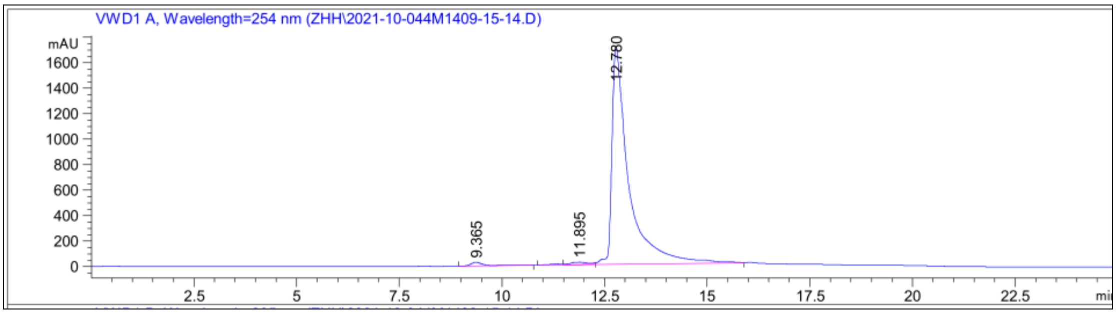

Peak #	RetTime [min]	Type	Width [min]	Area [mAU*s]	Height [mAU]	Area %
1	9.365	BV R	0.2910	645.30835	29.62616	1.2952

2	11.895	VV E	0.3724	520.21014	18.94187	1.0441
3	12.780	VV R	0.3831	4.86591e4	1707.44263	97.6608

*Compound **3m** (96.73%)*

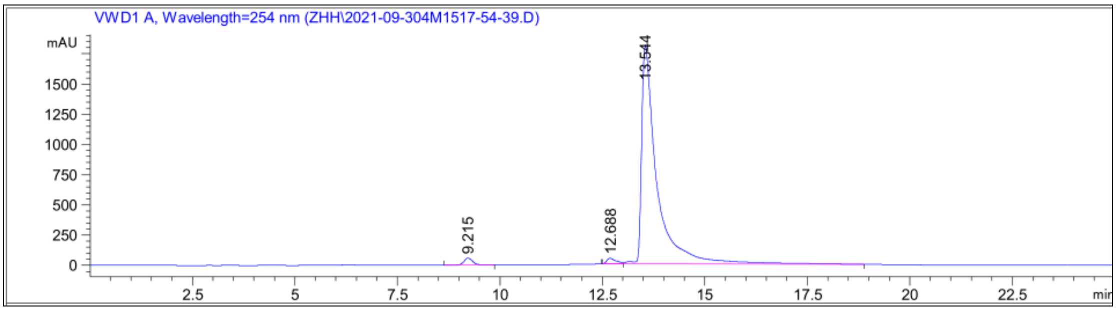

Peak #	RetTime [min]	Type	Width [min]	Area [mAU*s]	Height [mAU]	Area %
1	9.215	BB	0.2284	841.56879	57.61187	1.7114
2	12.688	BV E	0.2414	768.66034	46.85566	1.5632
3	13.544	VV R	0.3809	4.75629e4	1807.93433	96.7254

*Compound **3p** (96.85%)*

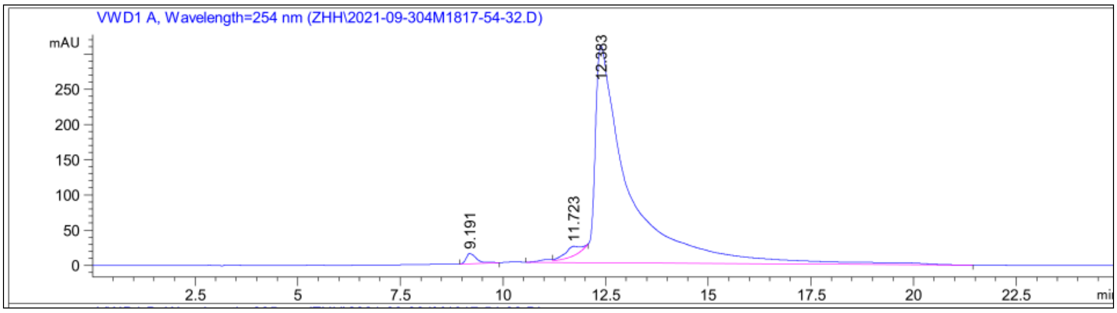

Peak #	RetTime [min]	Type	Width [min]	Area [mAU*s]	Height [mAU]	Area %
1	9.191	BV R	0.2771	250.19003	14.23428	1.3233
2	11.723	VV E	0.3591	346.15891	13.23492	1.8309
3	12.383	VB R	0.7671	1.83102e4	308.27759	96.8458

*Compound **3q** (97.58%)*

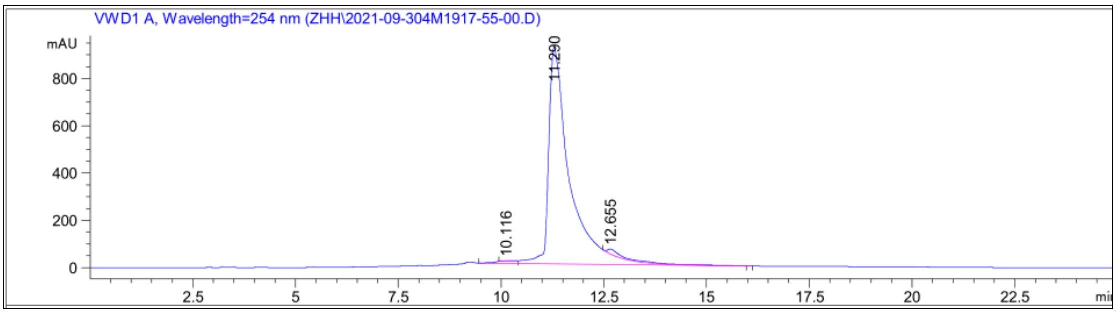

Peak #	RetTime [min]	Type	Width [min]	Area [mAU*s]	Height [mAU]	Area %

1	10.116	BV E	0.2502	29.00440	2.08663	0.0857
2	11.290	VV R	0.5232	3.30449e4	919.68774	97.5821
3	12.655	VB E	0.4915	789.79517	20.77492	2.3323

*Compound **3s** (95.21%)*

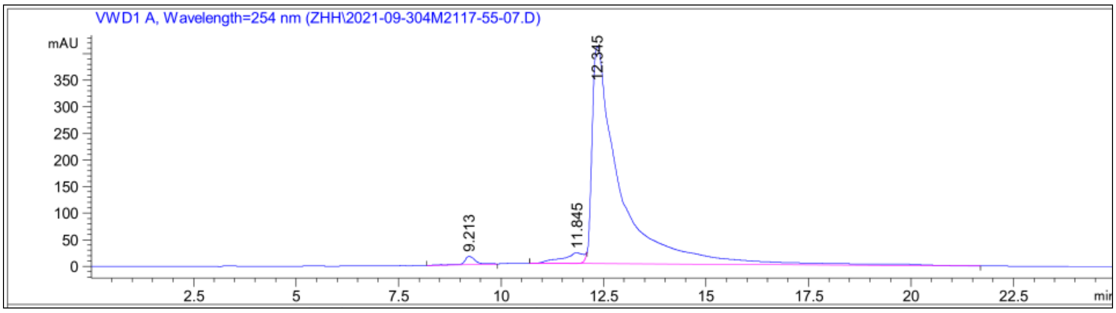

Peak #	RetTime [min]	Type	Width [min]	Area [mAU*s]	Height [mAU]	Area %
1	9.213	BV R	0.2795	280.43924	15.46843	1.3536
2	11.845	BV E	0.4700	712.02106	19.87464	3.4368
3	12.345	VB R	0.6499	1.97252e4	409.54944	95.2096

*Compound **3t** (97.42%)*

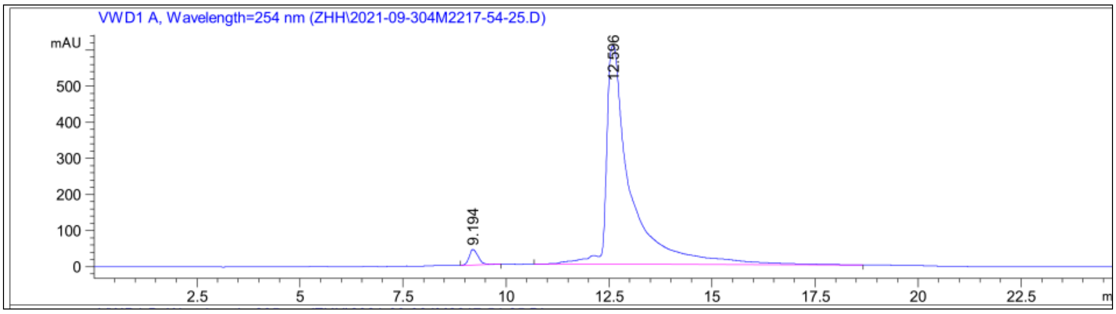

Peak #	RetTime [min]	Type	Width [min]	Area [mAU*s]	Height [mAU]	Area %
1	9.194	BV R	0.2420	654.82684	42.91409	2.5775
2	12.596	VB R	0.5482	2.47509e4	605.84290	97.4225

HRMS spectra of synthesized compounds

*Compound **2d***

**Sample Name**	Sample19	**Position**	P1-C1	**Instrument Name**	Instrument 1
**User Name**		**Inj Vol**	5	**InjPosition**	
**Sample Type**	Sample	**IRM Calibration Status**	Success	**Data Filename**	20211018-zhanghonghua-4H5.d
**ACQ Method**	0 750mz AB POS MSMS.m	**Comment**		**Acquired Time**	10/18/2021 5:56:39 PM (UTC+08:00)

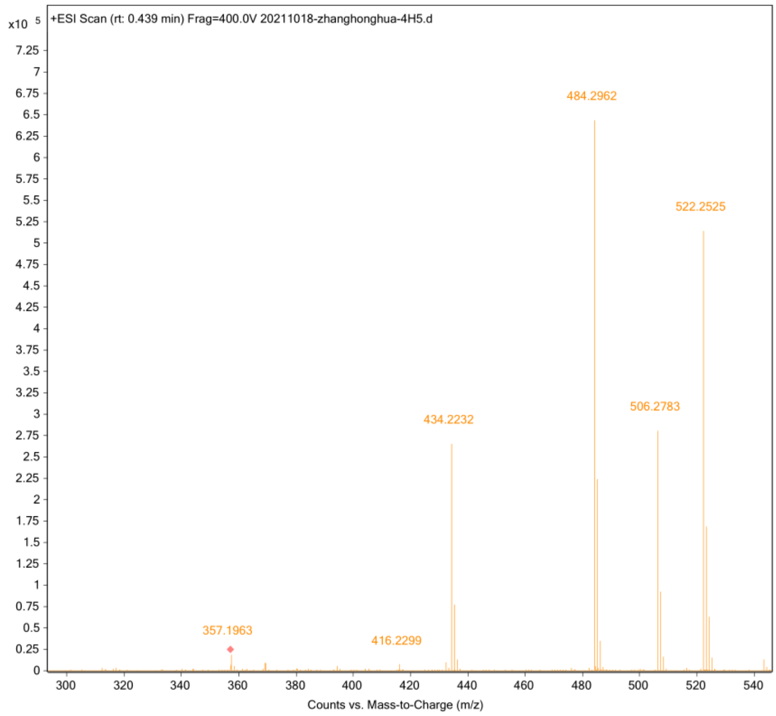

*Compound **2e***

**Sample Name**	Sample20	**Position**	P1-C2	**Instrument Name**	Instrument 1
**User Name**		**Inj Vol**	5	**InjPosition**	
**Sample Type**	Sample	**IRM Calibration Status**	Success	**Data Filename**	20211018-zhanghonghua-4H6.d
**ACQ Method**	0 750mz AB POS MSMS.m	**Comment**		**Acquired Time**	10/18/2021 5:58:13 PM (UTC+08:00)

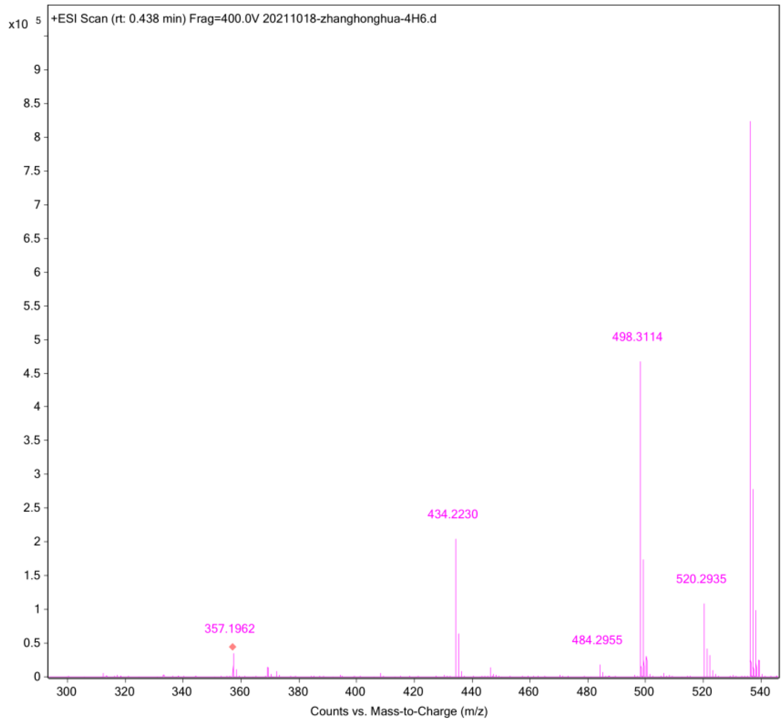

*Compound **2q***

**Sample Name**	Sample21	**Position**	P1-C3	**Instrument Name**	Instrument 1
**User Name**		**Inj Vol**	5	**InjPosition**	
**Sample Type**	Sample	**IRM Calibration Status**	Success	**Data Filename**	20211018-zhanghonghua-4H19.d
**ACQ Method**	0 750mz AB POS MSMS.m	**Comment**		**Acquired Time**	10/18/2021 5:59:47 PM (UTC+08:00)

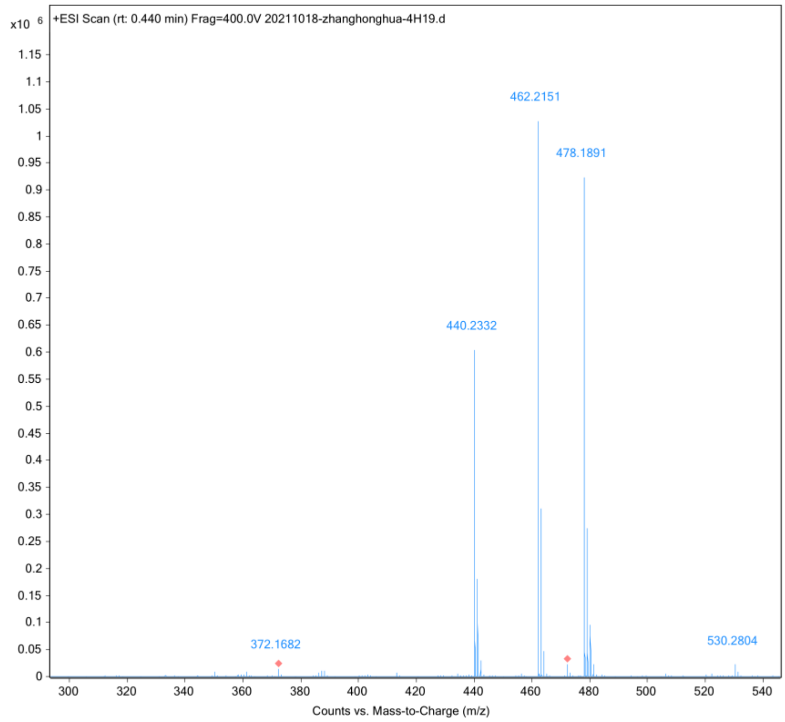

*Compound **3e***

**Sample Name**	Sample22	**Position**	P1-C4	**Instrument Name**	Instrument 1
**User Name**		**Inj Vol**	5	**InjPosition**	
**Sample Type**	Sample	**IRM Calibration Status**	Success	**Data Filename**	20211018-zhanghonghua-4M6.d
**ACQ Method**	0 750mz AB POS MSMS.m	**Comment**		**Acquired Time**	10/18/2021 6:01:21 PM (UTC+08:00)

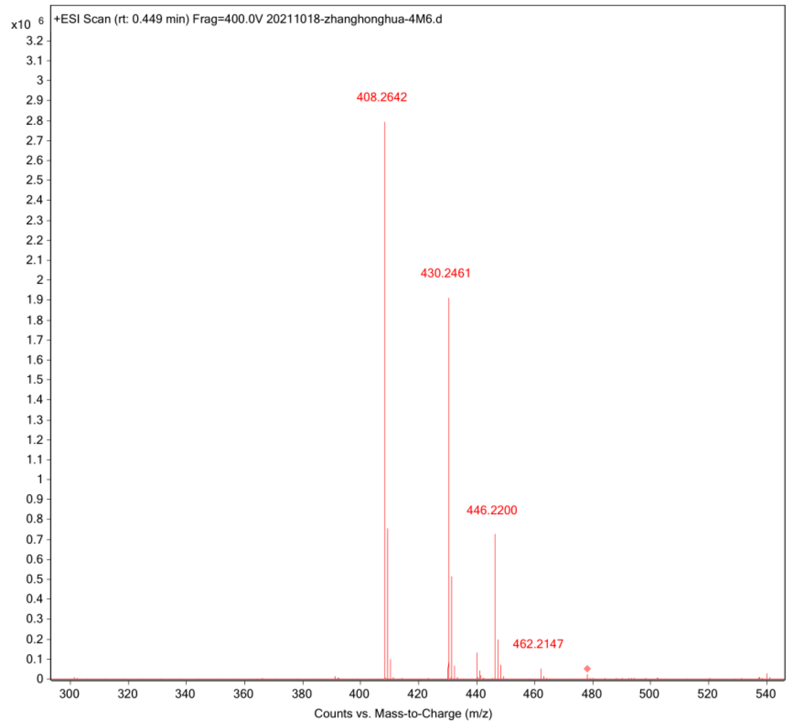

Compound ***3g***

**Sample Name**		**Position**	P1a2	**Instrument Name**	Instrument 1
**User Name**		**Inj Vol**	5	**InjPosition**	
**Sample Type**	Sample	**IRM Calibration Status**	Success	**Data Filename**	20210927-zhanghonghua-4M8-003.d
**ACQ Method**	0 750mz AB POS MSMS.m	**Comment**		**Acquired Time**	9/27/2021 10:56:41 AM (UTC+08:00)

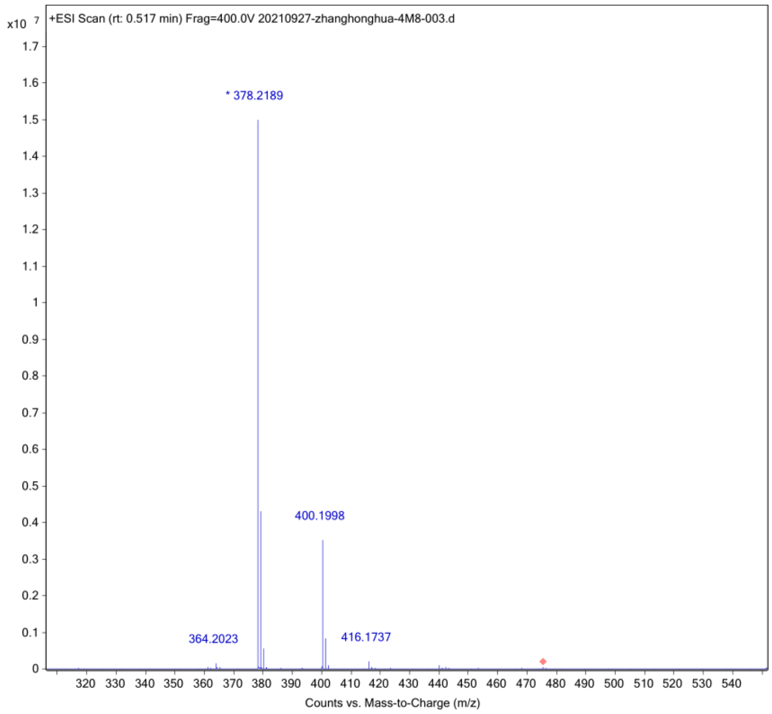

Compound ***3o***

**Sample Name**		**Position**	P1a3	**Instrument Name**	Instrument 1
**User Name**		**Inj Vol**	5	**InjPosition**	
**Sample Type**	Sample	**IRM Calibration Status**	Success	**Data Filename**	20210927-zhanghonghua-4M17-004.d
**ACQ Method**	0 750mz AB POS MSMS.m	**Comment**		**Acquired Time**	9/27/2021 10:58:26 AM (UTC+08:00)

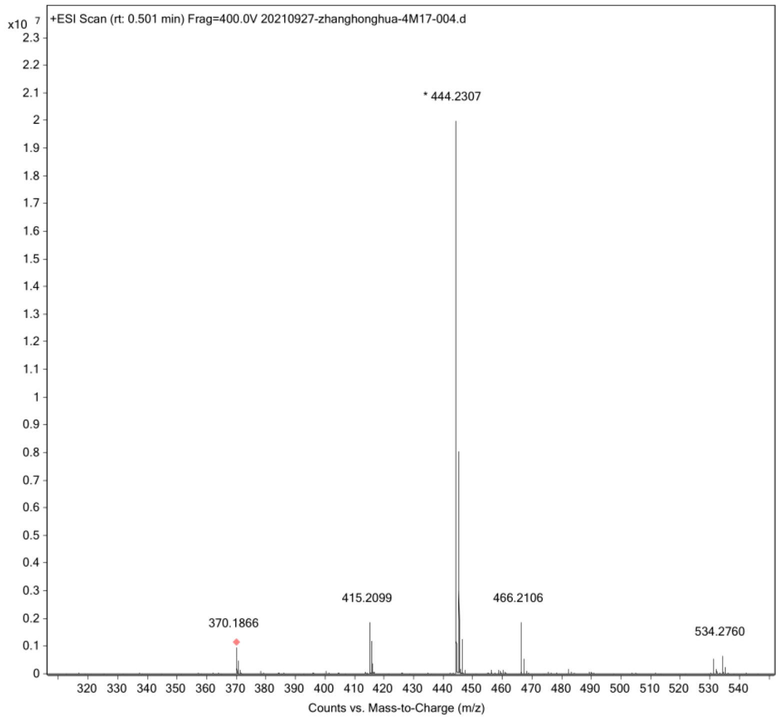

*Compound **3p***

**Sample Name**		**Position**	P1a4	**Instrument Name**	Instrument 1
**User Name**		**Inj Vol**	5	**InjPosition**	
**Sample Type**	Sample	**IRM Calibration Status**	Success	**Data Filename**	20210927-zhanghonghua-4M18-005.d
**ACQ Method**	0 750mz AB POS MSMS.m	**Comment**		**Acquired Time**	9/27/2021 11:00:18 AM (UTC+08:00)

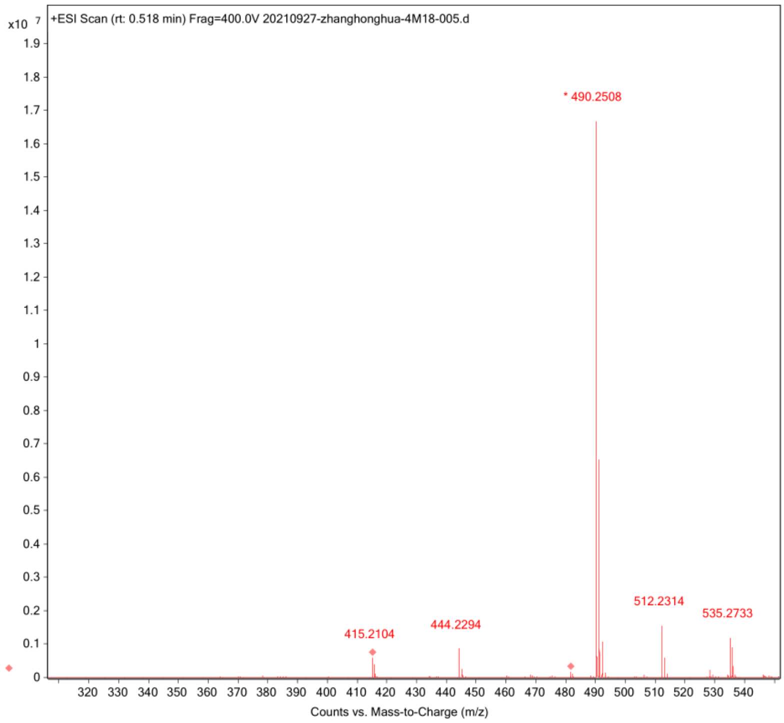

*Compound **3q***

**Sample Name**	Sample23	**Position**	P1-C5	**Instrument Name**	Instrument 1
**User Name**		**Inj Vol**	5	**InjPosition**	
**Sample Type**	Sample	**IRM Calibration Status**	Success	**Data Filename**	20211018-zhanghonghua-4M19.d
**ACQ Method**	0 750mz AB POS MSMS.m	**Comment**		**Acquired Time**	10/18/2021 6:02:54 PM (UTC+08:00)

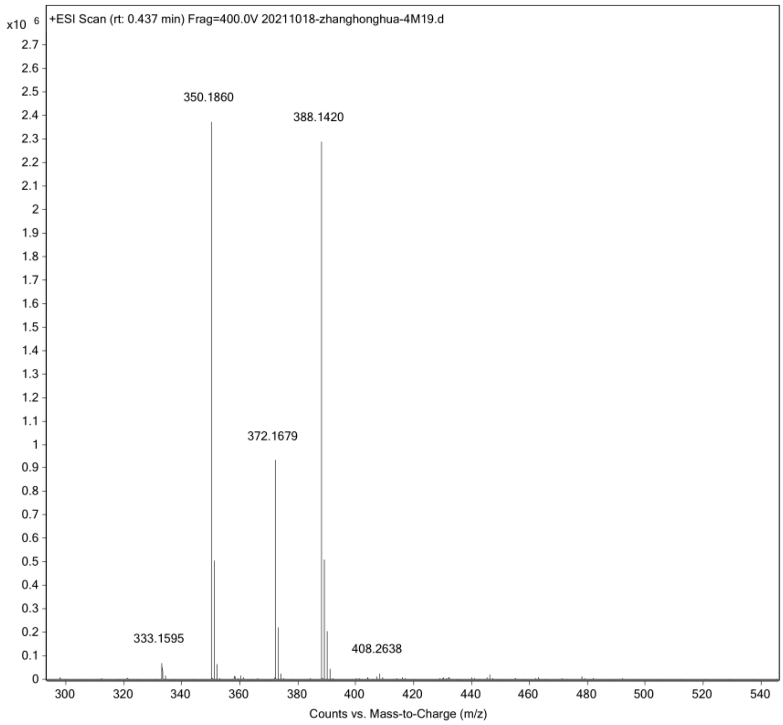

*Compound **3r***

**Sample Name**		**Position**	P1a1	**Instrument Name**	Instrument 1
**User Name**		**Inj Vol**	5	**InjPosition**	
**Sample Type**	Sample	**IRM Calibration Status**	Success	**Data Filename**	20210927-zhanghonghua-4M20-002.d
**ACQ Method**	0 750mz AB POS MSMS.m	**Comment**		**Acquired Time**	9/27/2021 10:54:47 AM (UTC+08:00)

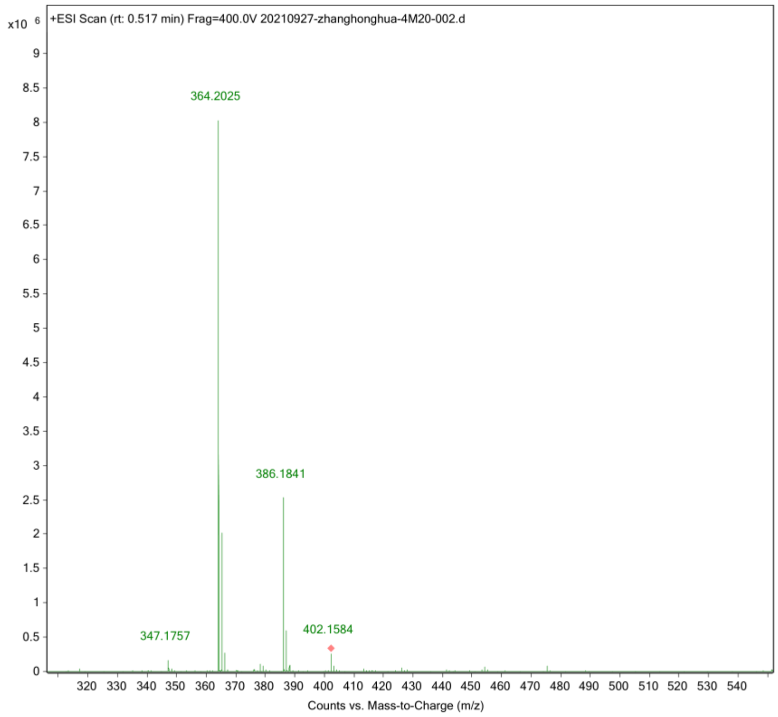

Molecular formula strings of synthesized compounds

Comp.	Smile String
**2a**	O=C(NCCC)OC1=CC=C(NC=C2CCN(CC3=CC=CC=C3)CC4=CC=CC=C4)C2=C1
**2b**	O=C(NCCCC)OC1=CC=C(NC=C2CCN(CC3=CC=CC=C3)CC4=CC=CC=C4)C2=C1
**2c**	O=C(NCCCCC)OC1=CC=C(NC=C2CCN(CC3=CC=CC=C3)CC4=CC=CC=C4)C2=C1
**2d**	O=C(NCCCCCC)OC1=CC=C(NC=C2CCN(CC3=CC=CC=C3)CC4=CC=CC=C4)C2=C1
**2e**	O=C(NCCCCCCC)OC1=CC=C(NC=C2CCN(CC3=CC=CC=C3)CC4=CC=CC=C4)C2=C1
**2f**	O=C(NC1CC1)OC2=CC=C(NC=C3CCN(CC4=CC=CC=C4)CC5=CC=CC=C5)C3=C2
**2g**	O=C(NC1CCCC1)OC2=CC=C(NC=C3CCN(CC4=CC=CC=C4)CC5=CC=CC=C5)C3=C2
**2h**	O=C(NC1CCCCC1)OC2=CC=C(NC=C3CCN(CC4=CC=CC=C4)CC5=CC=CC=C5)C3=C2
**2i**	O=C(NC1=CC=CC=C1)OC2=CC=C(NC=C3CCN(CC4=CC=CC=C4)CC5=CC=CC=C5)C3=C2
**2j**	O=C(N(C)C)OC1=CC=C(NC=C2CCN(CC3=CC=CC=C3)CC4=CC=CC=C4)C2=C1
**2k**	O=C(N(CC)CC)OC1=CC=C(NC=C2CCN(CC3=CC=CC=C3)CC4=CC=CC=C4)C2=C1
**2l**	O=C(N(CCC)CCC)OC1=CC=C(NC=C2CCN(CC3=CC=CC=C3)CC4=CC=CC=C4)C2=C1
**2m**	O=C(N(CCCC)CCCC)OC1=CC=C(NC=C2CCN(CC3=CC=CC=C3)CC4=CC=CC=C4)C2=C1
**2n**	O=C(N(C1=CC=CC=C1)C2=CC=CC=C2)OC3=CC=C(NC=C4CCN(CC5=CC=CC=C5)CC6=CC=CC=C6)C4=C3
**2o**	O=C(N(C)OC)OC1=CC=C(NC=C2CCN(CC3=CC=CC=C3)CC4=CC=CC=C4)C2=C1
**2p**	O=C(N(C)C1=CC=CC=C1)OC2=CC=C(NC=C3CCN(CC4=CC=CC=C4)CC5=CC=CC=C5)C3=C2
**2q**	O=C(N1CCC1)OC2=CC=C(NC=C3CCN(CC4=CC=CC=C4)CC5=CC=CC=C5)C3=C2

**2r**	O=C(N1CCCC1)OC2=CC=C(NC=C3CCN(CC4=CC=CC=C4)CC5=CC=CC=C5)C3=C2
**2s**	O=C(N1CCCCC1)OC2=CC=C(NC=C3CCN(CC4=CC=CC=C4)CC5=CC=CC=C5)C3=C2
**2t**	O=C(N1CCCCCC1)OC2=CC=C(NC=C3CCN(CC4=CC=CC=C4)CC5=CC=CC=C5)C3=C2
**2u**	O=C(N1CCOCC1)OC2=CC=C(NC=C3CCN(CC4=CC=CC=C4)CC5=CC=CC=C5)C3=C2
**3a**	O=C(NCCC)OC1=CC=C(NC=C2CCNCC3=CC=CC=C3)C2=C1
**3b**	O=C(NCCCC)OC1=CC=C(NC=C2CCNCC3=CC=CC=C3)C2=C1
**3c**	O=C(NCCCCC)OC1=CC=C(NC=C2CCNCC3=CC=CC=C3)C2=C1
**3d**	O=C(NCCCCCC)OC1=CC=C(NC=C2CCNCC3=CC=CC=C3)C2=C1
**3e**	O=C(NCCCCCCC)OC1=CC=C(NC=C2CCNCC3=CC=CC=C3)C2=C1
**3f**	O=C(NC1CC1)OC2=CC=C(NC=C3CCNCC4=CC=CC=C4)C3=C2
**3g**	O=C(NC1CCCC1)OC2=CC=C(NC=C3CCNCC4=CC=CC=C4)C3=C2
**3h**	O=C(NC1CCCCC1)OC2=CC=C(NC=C3CCNCC4=CC=CC=C4)C3=C2
**3i**	O=C(NC1=CC=CC=C1)OC2=CC=C(NC=C3CCNCC4=CC=CC=C4)C3=C2
**3j**	O=C(N(C)C)OC1=CC=C(NC=C2CCNCC3=CC=CC=C3)C2=C1
**3k**	O=C(N(CC)CC)OC1=CC=C(NC=C2CCNCC3=CC=CC=C3)C2=C1
**3l**	O=C(N(CCC)CCC)OC1=CC=C(NC=C2CCNCC3=CC=CC=C3)C2=C1
**3m**	O=C(N(CCCC)CCCC)OC1=CC=C(NC=C2CCNCC3=CC=CC=C3)C2=C1
**3n**	O=C(N(C1=CC=CC=C1)C2=CC=CC=C2)OC3=CC=C(NC=C4CCNCC5=CC=CC=C5)C4=C3
**3o**	O=C(N(C)OC)OC1=CC=C(NC=C2CCNCC3=CC=CC=C3)C2=C1
**3p**	O=C(N(C)C1=CC=CC=C1)OC2=CC=C(NC=C3CCNCC4=CC=CC=C4)C3=C2
**3q**	O=C(N1CCC1)OC2=CC=C(NC=C3CCNCC4=CC=CC=C4)C3=C2
**3r**	O=C(N1CCCC1)OC2=CC=C(NC=C3CCNCC4=CC=CC=C4)C3=C2
**3s**	O=C(N1CCCCC1)OC2=CC=C(NC=C3CCNCC4=CC=CC=C4)C3=C2
**3t**	O=C(N1CCCCCC1)OC2=CC=C(NC=C3CCNCC4=CC=CC=C4)C3=C2
**3u**	O=C(N1CCOCC1)OC2=CC=C(NC=C3CCNCC4=CC=CC=C4)C3=C2
**4c**	NCCC1=CNC2=CC=C(OC(NCCCCC)=O)C=C21
**4d**	NCCC1=CNC2=CC=C(OC(NCCCCCC)=O)C=C21
**4e**	NCCC1=CNC2=CC=C(OC(NCCCCCCC)=O)C=C21
